# LC-MS/DIA-based strategy for comprehensive flavonoid profiling: an *Ocotea* spp. applicability case†

**DOI:** 10.1039/d4ra01384k

**Published:** 2024-04-02

**Authors:** Matheus Fernandes Alves, Albert Katchborian-Neto, Paula Carolina Pires Bueno, Fausto Carnevale-Neto, Rosana Casoti, Miller Santos Ferreira, Michael Murgu, Ana Claudia Chagas de Paula, Danielle Ferreira Dias, Marisi Gomes Soares, Daniela Aparecida Chagas-Paula

**Affiliations:** a Institute of Chemistry, Federal University of Alfenas-MG 37130-001 Alfenas Minas Gerais Brazil daniela.chagas@unifal-mg.edu.br; b Leibniz Institute of Vegetable and Ornamental Crops (IGZ) Theodor-Echtermeyer-Weg 1 14979 Großbeeren Germany; c Northwest Metabolomics Research Center, Department of Anesthesiology and Pain Medicine, University of Washington 850 Republican Street Seattle Washington 98109 USA; d Antibiotics Department, Federal University of Pernambuco 50670-901 Recife Pernambuco Brazil; e Waters Corporation Alameda Tocantins 125, Alphaville 06455-020 São Paulo Brazil; f Faculty of Pharmacy, Federal University of Juiz de Fora 36036-900 Juiz de Fora Minas Gerais Brazil

## Abstract

We introduce a liquid chromatography – mass spectrometry with data-independent acquisition (LC-MS/DIA)-based strategy, specifically tailored to achieve comprehensive and reliable glycosylated flavonoid profiling. This approach facilitates in-depth and simultaneous exploration of all detected precursors and fragments during data processing, employing the widely-used open-source MZmine 3 software. It was applied to a dataset of six *Ocotea* plant species. This framework suggested 49 flavonoids potentially newly described for these plant species, alongside 45 known features within the genus. Flavonols kaempferol and quercetin, both exhibiting *O*-glycosylation patterns, were particularly prevalent. Gas-phase fragmentation reactions further supported these findings. For the first time, the apigenin flavone backbone was also annotated in most of the examined *Ocotea* species. Apigenin derivatives were found mainly in the *C*-glycoside form, with *O. porosa* displaying the highest flavone : flavonol ratio. The approach also allowed an unprecedented detection of kaempferol and quercetin in *O. porosa* species, and it has underscored the untapped potential of LC-MS/DIA data for broad and reliable flavonoid profiling. Our study annotated more than 50 flavonoid backbones in each species, surpassing the current literature.

## Abstract 2

A user-friendly LC-MS data-independent acquisition-based strategy using open software for access to the flavonoid content of complex mixtures.

## Graphical abstract

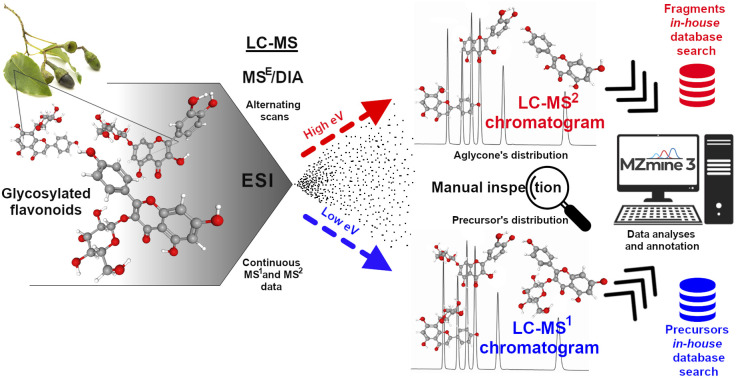

## 1 Introduction

In the dynamic field of natural product (NP) research, the quest for high coverage and accurate metabolite profiling is essential, particularly because of the presence of closely related compounds such as glycosylated flavonoids. These metabolites often present challenges in the automatic annotation process, thus there is a significant and current requirement for enhanced analytical characterization methods.^1–5^ Flavonoids, in general, are natural compounds widespread in plants, which are generally perceived as complex datasets in metabolomics studies. Plant species from the *Ocotea* genus are one of the richest in chemical diversity within the family of Lauraceae plants, while still considered a botanical challenge for precise identification and distinction.^6–8^ Lauraceae plants were highly recognized for their economic value in the past century, whereas several have demonstrated relevant medicinal potential, which is supported by traditional uses.^9,10^ Biological activities including anti-inflammatory, antimicrobial, and cytotoxic were reported for a range of *Ocotea* spp., which were attributed mainly to the presence of alkaloids, flavonoids, and lignoids.^10^ Recently, several benzylisoquinoline and aporphine alkaloids from 60 different *Ocotea* spp. were evidenced as potential biomarkers of anti-inflammatory activity.^11^ Moreover, flavonoids were found to be widespread in these species using advanced data-independent acquisition (DIA) molecular networking approaches.^12^

Recent phytochemical studies carried out by our group revealed the anti-inflammatory potential of *O. diospyrifolia* and *O. odorifera*, leading to the isolation of several NPs, including a novel aporphine alkaloid from the former, named diospirifoline.^13,14^ However, a gap in the complete chemical characterization of these plants persists, impeding comprehensive chemophenetic analyses.^15^ Chemophenetics is a recently proposed term to study the distribution and arrangement of NPs in a taxon, which is a crucial tool in the fields of chemosystematics and chemotaxonomy of plants.^16^ It enables the identification of biomarker distribution among species, which is invaluable, particularly for a genus such as *Ocotea*, in which morphological variability impacts species delimitation.^15,17^

**Fig. 1:**
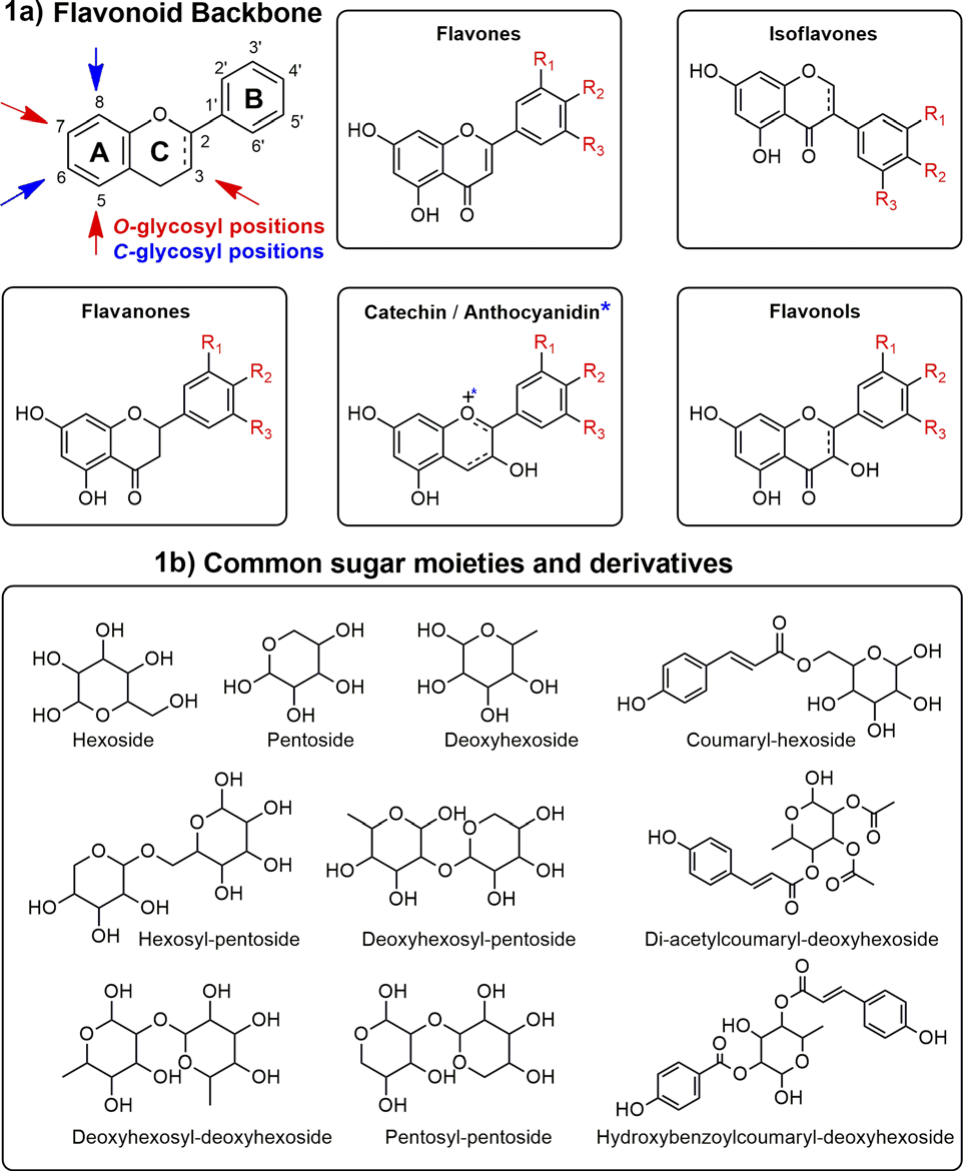
Main glycosylation types, aglycones, and sugars in flavonoids. This figure illustrates the diverse subclasses of flavonoids (1a) and the structural variations due to glycosylation (1b), highlighting the complexity and variety within the glycosylated flavonoid family.

In this context, most studies concerning *Ocotea* plants mainly focused on alkaloids,^11,18,19^ and lignoids,^20–23^ as this genus is recognized as a great producer of these NP classes.^15,24^ However, flavonoids were often underexploited, despite their renowned and extensive range of biological activities and health benefits. As part of the polyphenol family, flavonoids are characterized by their distinct 15-carbon skeleton, consisting of two phenyl rings (A and B) and a heterocyclic ring (C). This basic structure allows for the generation of a variety of subclasses, including flavones, flavonols, flavanones, flavan-3-ols, anthocyanidins, and isoflavones, each differing in the level of hydroxylation and other substitutions in their respective aromatic rings (Fig. 1a).^25,26^ In the context of glycosylated flavonoids, the presence of sugar moieties attached to the flavonoid backbone structure (Fig. 1b) often enhances their solubility, stability, and bioavailability, while still significantly altering their biological activities. However, the glycosylation in different backbone flavonoid positions also adds complexity to the profiling analyses and appropriate metabolite identification. The traditional analytical techniques often fall short in sensitivity, specificity, and throughput, underscoring the need for more robust and efficient methodologies.^3,27^ Modern analytical tools, combined with comprehensive annotation strategies can aid in addressing these drawbacks, also preventing redundant isolation and identification of already known metabolites.^28,29^

Liquid chromatography coupled with mass spectrometry (LC-MS)-based untargeted metabolomics represents a modern approach in NP research, with great potential for field advancement by enhancing metabolite coverage.^30,31^ This approach typically employs data-dependent acquisition (DDA) and data-independent acquisition (DIA) techniques, which are pivotal for acquiring both precursor (MS^1^) and fragment (MS^2^) ion data.^32,33^ DDA selects precursor ions based on their measured MS^1^ scan abundance, to acquire their corresponding MS^2^ spectra.^34^ In contrast, DIA indiscriminately fragments all detectable precursor ions within a larger mass range.^35^ Although DIA's methods, spectral libraries, and software are better established in the proteomics field,^36^ DIA development and applications in metabolomics are starting to rise.^37–39^ The classic DIA methods are based on alternation between low and high-energy channels, to acquire both MS^1^ and MS^2^ scans, respectively.^40,41^ The terminology varies across MS platforms. For example, in Waters quadrupole-time of flight (QTOF) instruments it is referred to as MS^*E*^, while in Thermo Fisher Orbitrap™ instruments it is termed all-ion fragmentation (AIF).^41^

Modern mass spectrometers have been able to perform sophisticated DIA experiments with varied defined isolation windows in sequence. The data generated for them has fewer interferences from co-eluting species and it is, consequently, easier for the deconvolution step. The sequential window acquisition of all theoretical mass spectra (SWATH™), and the SONAR™, in AB SCIEX and Waters QTOF instruments, respectively, are examples of these modern DIA experiments.^42,43^ Thermo Orbitrap™ instruments includes the variable data-independent acquisition (vDIA),^44^ and most recently, the narrow-window data-independent acquisition (nDIA) in Orbitrap™ with the asymmetric track lossless (Astral™) analyzer.^45^

In addition, ion mobility spectrometry (IMS) is an ancillary technique to high-resolution mass spectrometry (HRMS) that has been demonstrated to improve the quality of DIA data and has been implemented in NP research.^41,46^ This technique is based on gas-phase electrophoretic separation of ions based on their size and conformations, resulting in an additional dimension to retention time and mass-to-charge ratio (*m/z*): the collision cross-section.^47^

DDA is considered to exhibit superior MS^2^ spectral quality, however often fails to cover low-abundance metabolites, leaving a significant number of metabolic features without MS^2^ spectra for metabolite annotation.^39,41,48^ This limitation is primarily due to the time allocation for each scan type during the acquisition process. In such top n DDA methodologies, approximately 95% of the instrument's time is devoted to MS^2^ acquisition. Conversely, in AIF or MS^*E*^/DIA methods, there is an approximately equal distribution of time between MS^1^ and MS^2^ scans.^35^ This balanced approach results in superior coverage of both metabolite and fragment spectra. Consequently, AIF or MS^*E*^/DIA generates much more complex spectral data, providing a more comprehensive profile of metabolites.^49,50^

Our group recently developed an integrative workflow for processing MS^*E*^/DIA data and conducting molecular networking. This workflow was applied to a dataset of 60 *Ocotea* plant species, resulting in the annotation of several NP's.^12^ Although advancements in automated data handling in metabolomics, manual inspection and critical analysis of LC-MS data remain an indispensable step for achieving reliable results.^51,52^ Herein, we present an innovative DIA-based strategy for straightforward flavonoid profiling of metabolomics plant datasets. It is a user-friendly way to manually explore the full DIA data. Once most previous studies focus on MS^1^ data for analysis, and MS^2^ just for further compound annotation, our approach uses the MS^2^ data directly to analyze the flavonoid aglycone distribution throughout the samples.

With this strategy, we could annotate a higher number of flavonoids compared to the same species included in the previous work.^12^ The strategy for flavonoid profiling employs the new MZmine 3 version software, one of the most popular for MS data processing and downstream analysis. As an applicability case, leaf extracts of six plant species of the *Ocotea* genus (*O. diospyrifolia*, *O. guianensis*, *O. lancifolia*, *O. notata*, *O. odorifera*, and *O. porosa*), already known for flavonoid content in literature, were analyzed in an LC-MS system with a high-resolution QTOF analyzer in MS^*E*^/DIA configuration. This dataset not only demonstrated the versatility of the LC-MS/DIA strategy but also highlighted its robustness in profiling a diverse range of glycosylated flavonoids from the *Ocotea* genus.

Despite the acknowledged importance of *Ocotea* spp. as a source of bioactive compounds, comprehensive metabolomic studies focusing on flavonoids in this genus are scarce, involving few studies related to metabolic profiling,^12,53^ extraction procedures optimization,^54^ biological activity,^55–57^ and chemophenetic analysis.^58^ Classical phytochemistry approaches showed interesting biological activities of *Ocotea* sp. flavonoid fractions, as antioxidant and antibacterial,^59^ antiherpertic,^60^ fungicide,^61^ and antimycobacterial.^62^ However, these approaches generally allow characterization of the isolated majoritarian compounds, resulting in an incomplete overview of the flavonoid content. Our research addresses this gap by employing an analytical framework that combines the high resolution and sensitivity of LC-MS with the coverage of DIA.

The primary goal of this study, beyond the relevance of chemical characterization of the *Ocotea* species, and corroboration for future chemosystematic studies within the genus, is to underscore the utility and effectiveness of the LC-MS/DIA method in NP research, particularly in accelerating and enhancing the confidence and coverage of targeted glycosylated flavonoid profiling. We introduce a strategy option for manually exploring MS^2^ DIA data (detailed in the video), addressing the need for more robust methods in data analysis, as DIA gains space in NP metabolomics studies.

## 2 Experimental

### 2.1 Chemicals

LC-MS acetonitrile and formic acid were purchased from Sigma Aldrich (St Louis, MO, USA). Water was obtained using an 18 MΩ cm Millipore Milli-Q™ water purification system (Millipore, Bedford, MA, USA). High-performance liquid chromatography (HPLC) grade solvents; including hexane, methanol, and ethanol, were acquired from Sigma Aldrich (St Louis, MO, USA).

### 2.2 Sample collection

For the ultra-performance liquid chromatography (UPLC) coupled to HRMS/DIA data analyses, a small amount of plant material (1–3 leaves) from *O. diospyrifolia* (Meisn.) Mez, *O. guianensis* Aubl., *O. lancifolia* (Schott) Mez, *O. notata* (Nees & Mart.) Mez, *O. odorifera* Vell. Rohwer, and *O. porosa* (Nees & Mart.) Barroso were provided by the Brazilian Herbarium Leopoldo Krieger (CESJ, Federal University of Juiz de Fora – UFJF, Minas Gerais). The plants were deposited with the voucher specimens #CESJ 62011, #CESJ 44460, #CESJ 45567, #CESJ 62057, #CESJ 31144, and #CESJ 51738, respectively, and they can be consulted at https://specieslink.net/col/CESJ/. The study of these plants was registered on the National System for Governance of Genetic Heritage and Associated Traditional Knowledge under the registration number #A5A8F67, and can be consulted at https://sisgen.gov.br/paginas/pubpesqatividade.aspx.

### 2.3 Sample preparation

The plant material was ground using pistil and liquid nitrogen. In 2 mL Eppendorf tubes, 20 mg of the ground material was extracted with 2 mL of ethanol : Milli-Q™ water solution in a 7 : 3 (V/V) ratio with the aid of an ultrasound bath for 15 minutes at 35 °C (170 W, 50 kHz, L100 Schuster, China). The extracts were then centrifuged at 22 °C and 112 rcf (G-force) to collect the supernatant. The samples were subjected to clean-up by partitioning with HPLC-grade hexane (2 × 200 μL) to remove non-polar substances, followed by filtration through polytetrafluoroethylene (PTFE) syringe filters (pore size 0.22 μm), and dried using an Eppendorf Speed-Vac Concentrator Plus 5305 (Hamburg, DEU) for 3 h at 40 °C. The samples were prepared at a concentration of 1 mg mL^−1^ in water : acetonitrile, 1 : 1, (V/V), and kept in a freezer (−20 °C) until analyses.^63^

### 2.4 UPLC-HRMS/DIA data acquisition

Data acquisition was performed using a UPLC system coupled to an electrospray (ESI) source and a Xevo™ G2-XS QTOF instrument (Waters Corp., Milford, MA, USA). Quality control measures of pooled samples, injection order, and solvent blanks were done as reported on our previous publication with several other *Ocotea* species.^12^ Chromatographic separation was achieved using a C18 column (100 × 2.1 mm, 1.8 μm particle size diameter; ACQUITY UPLC™ HSS T3), and a mobile phase gradient at a flow rate of 0.5 mL min^−1^, composed of acetonitrile (B) and water (A). Both phases are acidified with 0.1% of formic acid. The chromatographic gradient began with an initial composition of 1% of B, followed by a transition to 15% of B in 0.1 min. Further changes in solvent composition occurred at 7.5 min (80% of B), 8.5 min (99% of B), and 8.6 min (1% of B), which was held until 10 min. The total runtime was 10 min, with an injected sample volume of 5 μL. Oven and shelf temperatures were set at 40 °C and 10 °C, respectively. The mass spectrometer was operated in MS^*E*^ acquisition mode with alternating high and low-energy scans, for positive and negative ionization modes. The ionization energy was set at 3 eV for low-energy scans and 25–40 eV for high-energy scans. ESI-MS at a resolution up to 40 000 with a full mass scan range set from 50 to 1000 *m/z* for functions 1 and 2 was applied. Instrument parameters, including cone voltage (40 V), capillary voltage (3.0 kV), cone gas flow (30 L h^−1^), auxiliary gas flow rate (10 L h^−1^), desolvation temperature (300 °C), source temperature (120 °C), and desolvation gas flow (600 L h^−1^), were optimized. High-purity nitrogen was employed for desolvation, collision, and cone gas. To ensure accuracy and reproducibility, a solution of leucine-encephalin was used as a lock mass with *m/z* 554.2622 (ESI^−^) and *m/z* 556.2768 (ESI^+^) for identification. MS data were continuously collected, and lock spray calibration was performed every 10 seconds.

### 2.5 Data processing

The obtained data in .raw format were converted to the standard format .mzML using the Waters2mzML 1.2.0, available on GitHub (https://github.com/AnP311/Waters2mzML).

Converted .mzML data were imported into MZmine v. 3.9.0 for data processing (mass detection, chromatogram building, deconvolution, isotope elimination, alignment, and gap filling). The following steps and main parameters were performed for MS^1^ level processing: mass detection (Mass detector: Centroid, noise level: 3 × 10^2^), chromatogram builder using ADAP algorithm (Filters: MS level filter, MS^1^; minimum consecutive scans, 5; minimum intensity for consecutive scans, 8 × 10^2^; minimum absolute height, 8 × 10^3^; *m/z* tolerance, 0.005 *m/z* or 10 ppm), deconvolution using the local minimum resolver algorithm (dimension: retention time; chromatographic threshold, 0.85; minimum search range for retention time, 0.035; minimum absolute height, 8 × 10^3^; minimum peak top/edge ratio, 1.8; peak duration range, 0–2 min; minimum scans, 5), isotope elimination using the ^13^C isotope filter (*m/z* tolerance, 0.003 *m/z* or 5 ppm; retention time tolerance, 0.05 min; monotonic shape, yes; maximum charge, 1; representative isotope, most intense). Alignment using the Join aligner algorithm (*m/z* tolerance, 0.007 *m/z* or 12 ppm; *m/z* weight, 3; retention time tolerance, 0.045 min; retention time weight, 2). Additionally, gap filling (intensity tolerance, 0.2; *m/z* tolerance, 0.005 *m/z* or 7 ppm; retention time tolerance, 0.04 min; minimum scans, 4) was performed to obtain the final feature lists (*m/z*, retention time, and intensity). Subsequently, for MS^2^ processing, the same steps and parameters were performed using the MS level filter set to MS^2^ in the ADAP chromatogram builder step, and adjusting the minimum absolute height to 4 × 10^3^ in this step and also in resolving. Parameters (ESI 1 and 2†) and batch mode files from MZmine (ESI 3†) are available as ESI† at Zenodo's link described in data availability section; https://doi.org/10.5281/zenodo.10810967.

### 2.6 Databases construction

For compliance with this DIA strategy for flavonoid profiling, it is crucial to curate chemical databases for the subsequent automated annotation step. Thus, custom in-house databases, named *Ocotea_flavDB* (MS^1^), *Br_flavDB* (MS^1^), and *FlavAglyDB* (MS^2^) (available to download at ESI 4–6† at Zenodo's link https://doi.org/10.5281/zenodo.10810967) were previously prepared using SMILES as chemical structural data. To automate and speed up database construction, an .mol2 file based workflow was built on the Konstanz Information Miner (KNIME) platform (https://www.knime.com/) version 4.6.5 (University of Konstanz, Zurich, CHE). The workflow (ESI 7 – Fig. S1†) is online and available to download and utilize (https://hub.knime.com/-/spaces/-/∼ffDFj4alnx3YUYUt/).

*Ocotea_flavDB* consisted of 54 carefully curated flavonoid chemical structures previously isolated from plant species of the *Ocotea* genus. For that, we have utilized original published NP research articles in literature, and the Nuclei of Bioassays, Ecophysiology and Biosynthesis of Natural Products Database (NuBBE_DB_) (https://nubbe.iq.unesp.br/portal/nubbe-search.html) online database. NuBBE_DB_ currently stores more than 2200 NPs from Brazilian plant species and allows users to download and extract useful data information, which has been manually curated to ensure reliability. Thus, the manual construction of specific plant species, genera, and family databases is more feasible and trustworthy.^64,65^

In addition, we have also constructed the *Br_flavDB*, encompassing 288 flavonoids isolated from Brazilian plant species, which are currently available on NuBBE_DB_ (accessed January of 2024). These are MS^1^ databases and consist of comma-separated values (.csv) files containing the columns of the precursor flavonoid name, the high-resolution neutral mass, the molecular formula, the retention time (equal to the total time of the chromatographic method), and other information.

Another database, the MS^2^ database *FlavAgly*DB which also was built, comprised aglycone fragments of the main well-known flavonoids: kaempferol and isomers (datiscetin and luteolin), quercetin, catechin/epicatechin, apigenin, taxifolin, narigenin, and myricetin. These flavonoids were selected based on previous *Ocotea* biosynthetic chemical knowledge and can be adapted to any flavonoid aglycone, following the general fragments detailed in the ESI (ESI 8 at Zenodo's link https://doi.org/10.5281/zenodo.10810967).† The fragments were proposed based on the main gas-phase cleavage patterns of *O*- and *C*-glycosylated flavonoids in the literature.^66–69^ For the first type, heterolytic and homolytic cleavages were considered. For *C*-glycoconjugates, heterolytic cleavage resulting in modified aglycones with –C_3_H_6_O_2_ and –C_2_H_3_O residues derived from sugar moiety were proposed. Although some patterns occur in flavonoid substitution (*e.g.* flavonols *O*-substituted, and flavones *C*-substituted), we propose *O-* and *C-* cleavage types to all flavonoids of *FlavAglyDB.* These fragmentation reactions are shown in our results and discussion section. The MS^2^ database also consists of a .csv file, containing the columns of the aglycone fragments, the *m/z*, and the retention time (equal to the total time of the chromatographic method). In the case of *FlavAgly*DB, the *m/z* values are referred t to the [M − H]^−^ ions.

### 2.7 Flavonoid profiling

For the annotation step, the local compound database search module of MZmine 3 was employed for both MS^1^ and MS^2^ feature lists. Only data acquired in the negative ionization mode was used for glycosylated flavonoid profiling. Whereas, positive mode data was explored for manual complementary aglycone analysis. The following parameters were set for both custom in-house DBs: *m/z* tolerance at 0.005 *m/z* or 5 ppm; retention time tolerance, equal to the total time of the chromatographic method (10 minutes); use of adducts (only for MS^1^ databases), MS mode negative, maximum charge equal 2, maximum molecules/cluster equal 2, and [M − H]^−^, [M + Cl]^−^, [M + Br]^−^ and [M + FA]^−^ adducts. The last option was set only for the MS^1^ databases because they contain the monoisotopic neutral masses of the compounds. Very important to point out, that for this MZmine module to be functional, the exact name of each column of the .csv database file has to be indicated on the parameter setting (see SV1†).

By these means, annotation was initially conducted by matching the monoisotopic mass with the entries of the databases, achieving the flavonoid candidates on MS^1^-level spectra, and aglycones annotation on MS^2^-level spectra. The intense annotated aglycones (MS^2^ features) without correspondent MS^1^ flavonoid annotation, were further manually inspected for their corresponding MS^1^ feature data, to search for neutral losses. This involved manually searching for precursor ions that matched in retention time and exhibited similar peak shapes. This method of scan-level correlation significantly streamlined the flavonoid annotation process.

Additionally, the *FlavonoidSearch* tool v1.2.0 (https://sakura-kagaku.com/komics/software/FlavonoidSearch/) was employed for isomeric distinctions between flavonols such as kaempferol, datiscetin, and luteolin. This tool encompasses a comprehensive database of probable mass fragments for known flavonoids and a computational tool for database searching. This ensures enhanced accuracy and a deeper understanding of flavonoid diversity. *FlavonoidSearch* operates by using mass spectra of metabolite peaks in positive ionization mode as queries, thus facilitating the automatic identification of flavonoids.^70^

### 2.8 Step-by-step of the strategy

For the application of the proposed strategy, MZmine 3 is applied for comprehensive data visualization, interpretation, and independent processing of MS^1^ and MS^2^ data, which can be replicated and applied for different data processing settings. This methodology, detailed here, is adaptable for any MS^*E*^ or AIF LC-MS analysis, although herein was specifically for targeted analysis of glycosylated flavonoids. The following steps are shown in the protocol video attachment (Supplementary video 1†).

Step 1 starts with the generation of independent base peak chromatograms for both MS^1^ and MS^2^ levels.

Step 2 consists of a chromatogram comparison. Manually comparing MS^1^ and MS^2^ chromatograms allows researchers to directly compare high-intensity precursors with their fragments.

Step 3 is the overview of blank data, to identify non-significant peaks. Steps 1–3 are based on raw data and are depicted in the supplemental video.† This process is important for data first view and to further ensure accuracy for peak annotation.

Step 4 is the generation of feature lists. Following the initial data processing and parameters set up, which in our case is outlined in Section 2.5. Steps for feature list generation (mass detection, chromatogram building, resolving, alignment, gap filling, and annotation) can be done individually or in a batch. A batch mode file, including steps for feature list generation, is ready to adjust the parameters according to your sample acquisition and available on ESI (Supplementary video 1).† Two types of aligned feature lists are created: one for precursors (MS^1^) and another for fragments (MS^2^). These lists were refined by incorporating candidate flavonoids and aglycone hits from our in-house databases, corresponding to Sections 2.6 and 2.7.

Step 5 consists of the manual inspection of these features. It is conducted by feature lists and raw data analysis, focusing on retention time and peak shapes to ensure that fragments and their precursors exhibited co-elution with similar detection ratios. For instance, when examining fragment features at a retention time associated with a candidate precursor, it is anticipated that, for most cases, the MS^2^ features would display lower intensity than the MS^1^ precursor and share similar peak shapes.

Biosynthetic investigation. That is not a mandatory step and although it suits the hits. It can be performed to ensure confidence in level 3 annotations as per the Metabolomics Standards Initiative (MSI).^71^ To this, the Kyoto Encyclopedia of Genes and Genomes (KEGG) is a suitable online tool that can aid in mapping the flavonoid metabolic pathways.^72^

Critical structural analysis. The proposed structures from level 3 annotations guide the development of fragmentation hypotheses, aiding in the identification of candidate fragments. This approach allows researchers to explore and hypothesize about the structural components of NPs in respective studies. The strategy also enables the visualization of the distribution of the potential diagnostic ions of aglycone patterns observed across different samples in the dataset. Particularly, the manual search for diagnostic fragments is a feature that can be crucial in studies focused on complex NP profiling. In addition, new tools can be also integrated, such as the *FlavonoidSearch*, which employs structure- and fragmentation-related rules for flavonoid annotation, Section 2.7.

### 2.9 Further data analysis, figures, and graphs

Data analysis and visualization were also conducted using Python libraries. Pandas was utilized for data cleaning and manipulation. NumPy was used to support numerical calculations. Seaborn to perform and illustrate heatmap correlations, and Matplotlib for the creation of the graphs, including Venn diagrams using the Matplotlib-Venn extension. The Python libraries were accessed using the Google Colab online cloud service (https://colab.research.google.com/). All graphs were exported in .png format at 500 dpi resolution. Chemical structures were manipulated using the ChemDraw Ultra software version 12.0 (PerkinElmer Inc., Waltham, MA, USA). The high-resolution Figures were designed and processed using the open GIMP software version 2.10.34 (https://www.gimp.org/). The video was edited in CapCut applicative (https://www.capcut.com).

## 3 Results and discussion

Data processing is one of the hardest and most important steps to achieve a reliable annotation process. However, there is a significant challenge in DIA data processing, in particular, the deconvolution of MS^2^ spectra and the reassociation of precursor ions with their corresponding fragments.^49,73^ While vendor-specific software typically offers robust algorithms for DIA processing, *e.g.* UNIFI from Waters, the contribution of Tsugawa *et al.* is particularly noteworthy. They developed MS-DIAL software, an open-source alternative that has enriched the field with the MS^2^Dec algorithm, adept at successfully deconvoluting the DIA data algorithm.^73^ The advent of MS-DIAL marked the beginning of a new era of open DIA tools and software development in the metabolomics field.^74–76^ Examples of recent software for DIA data processing are DecoID and MetaboAnnotatoR.^77,78^ Besides, MZmine 3,^79^ the most popular open tool for MS data processing, despite a major development in the implementation of sophisticated tools, currently still lacks robust automated modules for LC-MS/DIA data processing and export.

More recent alternatives to explore DIA data potential are based on the integration of DDA data acquired from the same samples, named DIAMetAnalyser,^38^ and data-dependent-assisted data-independent acquisition (DaDIA),^80^ as well as the combination of different collision energies in a single analytical run.^48^ In addition, constructing in-house MS^2^ spectral databases from chemical compounds and integrating them into data processing are also effective strategies for refining DIA analysis.^81,82^ However, these strategies depend on the availability of LC-MS systems and authentic chemical standards. Additionally, for the context of the above-mentioned DaDIA integrated strategies, for most DIA data available in public repositories, *e.g.* Metabolights, MassIVE, and MetabolomeXchange, the appropriate correspondent DDA data is not always available. Under these circumstances, for metabolomic studies, the classical approach to MS-DIAL or vendor software is still sought. Alternatively, as our results demonstrate, MZmine 3 can provide efficient manual handling and inspection of DIA data, as the current version offers optimal data visualization modules.^79^ Yet, the challenges in MZmine 3 remain in accurately relinking fragments to their respective precursors, crucial for downstream analysis and data export to platforms such as the Global Natural Product Social Molecular Networking (GNPS).^83–85^

Thus, to enhance our analytical capabilities, the primary aim was to refine the processing and management of MS^2^ data. This advancement facilitates the streamlined manual annotation of flavonoids by enabling the direct mapping of chromatogram points associated with the aglycones of interest. Although MS-DIAL automates data processing, reconstructing MS^2^ spectra and linking them to their MS^1^ counterparts, it falls short in offering a holistic view of fragment data due to its inability to generate comprehensive feature lists. In contrast, MZmine 3 stands out for its versatility, supporting the incorporation of MS^2^ filters and allowing for data processing similar to conventional MS^1^ methodologies. This approach, seemingly unexplored in the scientific community, holds significant potential for advancing analytical capacity for enhanced flavonoid profiling.

In this context, the use of LC-MS/DIA, coupled with the advanced features of MZmine 3, has enabled a comprehensive and detailed exploration of our datasets. This DIA-based approach has uncovered a broad spectrum of glycosylated flavonoid compounds in the *Ocotea* species, providing crucial information regarding the presence of different aglycone and glycan fragments.^3,86^ The software stands out for its effective data processing, intuitive and integrative learning curve about processing steps, and particularly for its user-friendly and advanced visualization capabilities. These features render MZmine 3 a valuable tool in metabolomics, offering robust solutions for navigating through LC-MS data complexities. Critical to our methodology were the meticulous phases of manual inspection and analysis. They were not just vital for accuracy, but also for ensuring the reliability of our metabolite annotations. The sophisticated combination of these methods was key in diving deep into the complex layers of these intricate NP extracts. In this way, our approach effectively showcased the immense potential of DIA in uncovering the hidden chemical diversity within the *Ocotea* genus. This includes annotating high and low-abundance flavonoids and often potentially non-reported ones. The results of our investigation strongly advocate for the merits of integrated automated and manual methodological approaches.

**Fig. 2:**
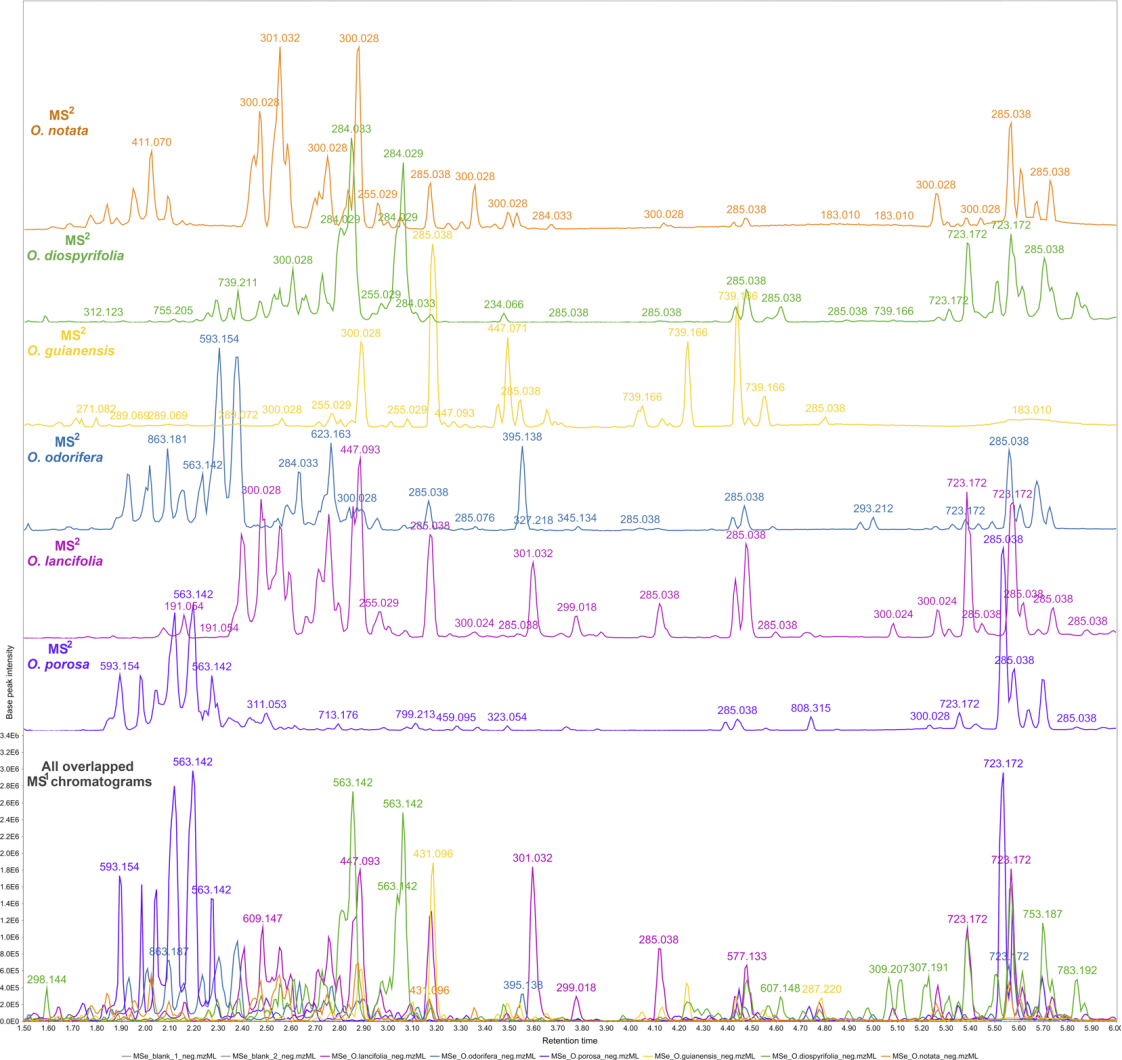
Overlapped and aligned LC-HRMS/DIA (MS^1^ and MS^2^) base peak intensity (BPI) chromatograms of *Ocotea* spp. leaf extracts in negative ionization mode in a 1.5–6.0 min retention time range.

The reported hits in this study are based on the feature monoisotopic mass matching with the respective in-house databases. The generated MS^1^ and MS^2^ chromatograms (ESI 7 – Fig. S2–S13†) displayed distinct peaks at the retention time range of 1.5–6 min, indicating the presence of glycosylated flavonoids at MS^1^ and their respective potential fragmented aglycones at MS^2^ spectra. By applying the proposed strategy for DIA data processing, this can be further evidenced through overlaying MS^1^ and MS^2^ chromatograms (Fig. 2). That made it possible to examine continuously each one of the chromatograms regarding the distributions of these flavonoid ions, thus, highlighting several high-intensity precursors corresponding to fragment ions with similar peak shapes at the same retention times. The possibility of MS^2^ data processing and continuous visualisation is a particularity of AIF or MS^*E*^/DIA data, where the same instrument time is spent to acquire both MS^1^ and MS^2^ data.^35^ In the MZmine 3 workflow, the algorithms are usually applied to MS^1^ processing to further attribute MS^2^ spectra to processed features (precursors).^79^ By applying these same algorithms to the DIA-MS^2^ level spectra, we have achieved an MS^2^ feature table, which is a dataset of all fragments represented by resolved features. We could analyze the fragments from a holistic point of view, as they were uninterruptedly distributed over the DIA data in the same way that the precursors were.

The MS^1^ processing resulted in 1603 aligned features, with 189 hits from *Ocotea_flavDB*. According to the MSI, they represent annotations at level 3 of confidence.^71^ These annotations, even though level 3, gained reliability once they originated from a specific database containing related-to-genus NPs, thus accounting only for flavonoids previously isolated in *Ocotea* spp. Considering biosynthetic aspects, where specific genes and consequently enzymes are shared among families or genera,^26^ this strategy is useful to obtain more realistic annotations for the studied species, avoiding meaningless annotations.^52^

In addition, we achieved 193 hits from *Br_flavDB*, which also contributed to level 3 confidence annotations. These hits include several known flavonoids not previously reported in *Ocotea* spp., thus expanding the scope of flavonoid profiling as they originate from plant species present in the Brazilian flora. The details of each of those annotations are shown in the ESI (ESI 7 – Tables S1 and S2).†

**Fig. 3:**
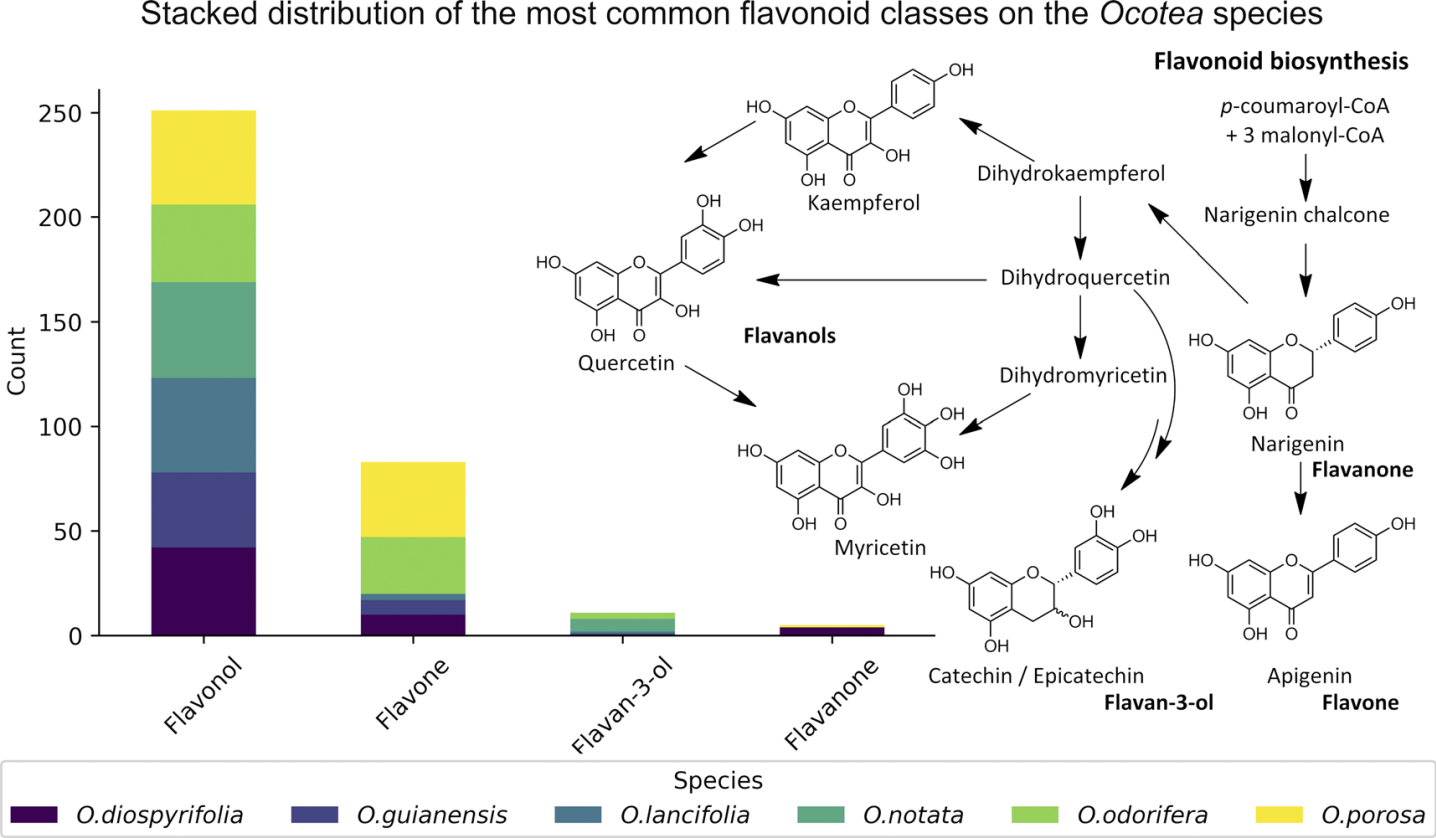
Stack bar graphs of flavonoid types. This figure displays the distribution of main flavonoid backbones among different *Ocotea* species, as annotated by MS^2^ processing. The stacked bars represent the various flavonoid subclasses and their respective biosynthetic pathways on the right side.

The MS^2^ processing aligned all *Ocotea* samples, resulting in 2021 features with 216 hits from *FlavAglyDB*. These features represent the fragment chromatogram peaks extracted from the continuous data, as we have done for the MS^1^ features. This approach is possible due to the versatility of MZmine 3 filters to step-by-step processing, where it is possible to perform the common mass detection, chromatogram building, resolving (deconvolution), deisotoping, and alignment steps also to the MS^2^ data. This allowed us to explore all information about the fragments distributed over the chromatograms. Such an approach facilitates the manual annotation of the processing features, as it gives a clear idea of the most intense fragments at the same retention time. Furthermore, some fragments are considered diagnostic ions of certain compound classes, and thus their presence can reveal important information about the respective chemical structure.^87,88^ For example, in the case of flavonoids, aglycone fragments directly indicate the NP class, and ring substitution patterns, which can aid in discriminating among possible isomers and subclasses.^89,90^ This demonstrates the effectiveness of MZmine 3 versatile filters in step-by-step processing, facilitating manual annotation of features and revealing crucial information for targeted profiling. On the other hand, the MS^2^ hits, related to the putative identified aglycones, provide an upfront view of the flavonoid main backbone distribution among these species (Fig. 3). All this information can be crucial for identifying dominant flavonoid subclasses and understanding related biosynthetic pathways.

Additionally, we navigated through the complex isomeric landscape of kaempferol, datiscetin, and luteolin, which were all present in our in-house databases. Leveraging the advanced capabilities of the *FlavonoidSearch* tool, which utilizes the Jaccard index for spectra similarity scoring, thus focusing on fragmentation patterns comparison.^70^ This strategic approach, utilizing data garnered from positive ionization, was pivotal in inspecting distinct features at *m/z* 287.0550 across all *Ocotea* species under examination. This method allowed refined discrimination, enabling us to conclusively ascertain the presence of either kaempferol or datiscetin over luteolin, guided by the most compelling scores (ESI 7 – Fig. S14†). Further delving into the flavonoid profile, our decision to annotate kaempferol was informed by its prevalent role as a fundamental flavonoid backbone within the *Ocotea* genus, standing alongside other significant flavonoids that include the quercetin and the catechin/epicatechin.^15^ Therefore, the proposed strategy enables a comprehensive search for the aglycone fragments and could be complemented with other currently available tools.

**Fig. 4:**
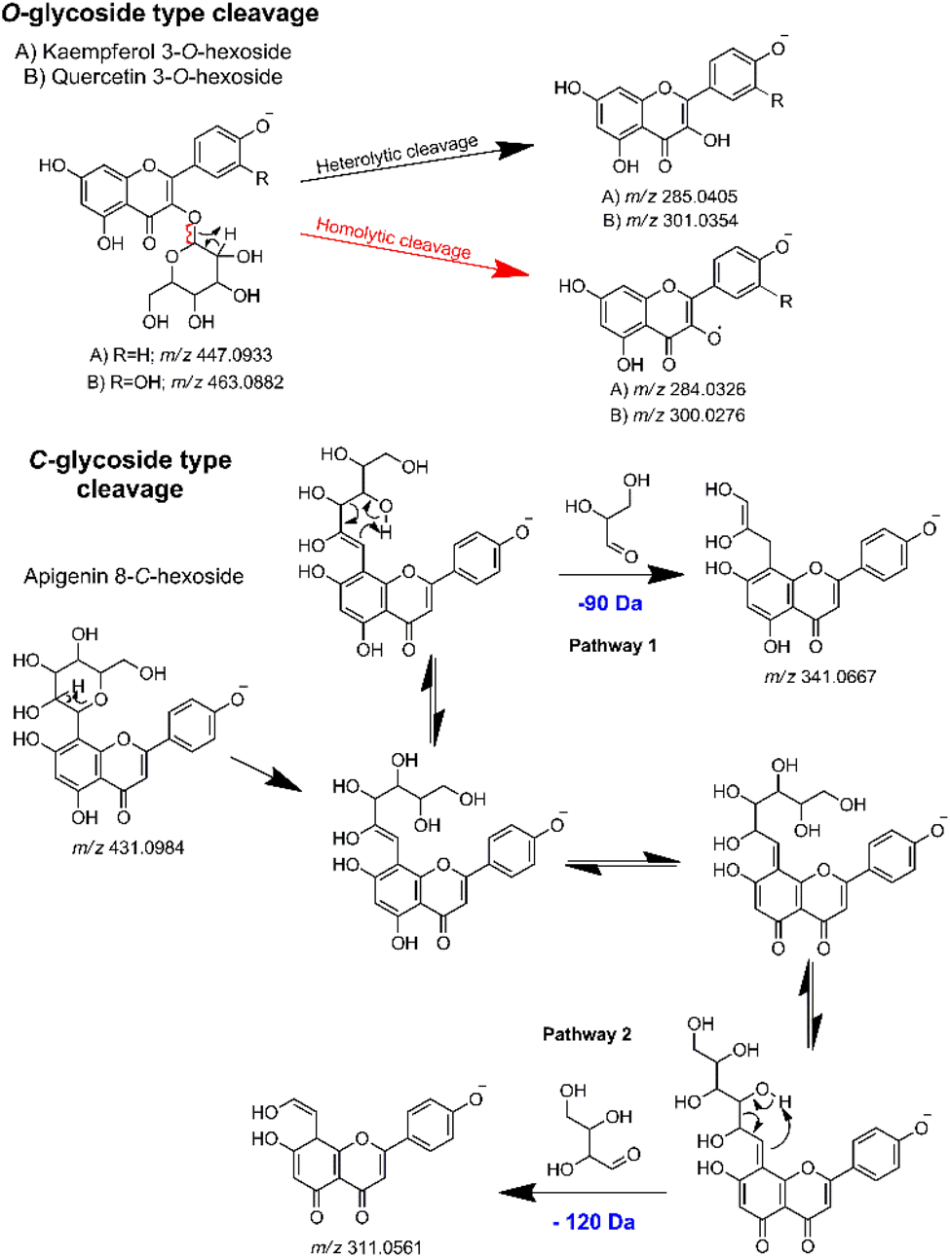
Key gas-phase fragmentation pathways for *O-* and *C*-type glycosides, highlighting the main glycoside cleavages.

Thus, in the MS^2^ feature table, which was acquired in the negative mode, the presence of several glycosylated derivatives of kaempferol (286.0477 Da) and quercetin (302.0427 Da) were evidenced by the *m/z* values 285.0386 (−6.67 ppm) and 301.0337 (−5.65 ppm), along with the 284.0321 (−1.76 ppm) and 300.0272 (−1.33 ppm), corresponding respectively to the heterolytic and homolytic cleavage of *O*-glycosylated flavonoids.^91^ As per their structure, *O*-flavonoid glycosides are capable of undergoing both types of cleavage, a phenomenon well-established in ESI ionization tandem MS for this class of NPs.^67,92,93^ Additionally, apigenin *C*-glycosides were identified by the modified aglycones, exemplified by diagnostic ions at *m/z*'s 311.0555 (−1.93 ppm), 341.0661 (−1.76 ppm), 353.0673 (1.69 ppm) and 383.0777 (1.04 ppm).^68,69^ Then, by subtracting the aglycone *m/z* value from the respective MS^1^ feature, with the same retention time and peak shape, it was possible to establish the neutral loss related to the aglycone moiety of each hit compound. The gas-phase fragmentation pathways involved in glycoside cleavage were proposed for the main *O-* and *C-*type glycosides observed (Fig. 4).

Detailed information regarding the aglycones and sugars derived from heterolytic and homolytic *O*-cleavage are tabulated in the ESI (ESI 7 – Table S3; and ESI 8 at Zenodo's link https://doi.org/10.5281/zenodo.10810967).† The complete MS^2^ aglycone annotation lists are also included in the ESI (ESI 7 – Table S4).† Furthermore, determining the specific site at which sugar attaches to a flavonoid backbone without relying on highly controlled experiments and authentic standards, is not a simple task. Therefore, we have adopted a more reliable approach to annotate and report these NPs (Table 1).

**Table 1:** Representative examples of *Ocotea* flavonoids in LC-MS/DIA conducted in negative ionization mode, based on our custom in-house databases

RT (min)	MS^1^*m/z*	MS^1^ precursor's annotation	Error (ppm)	MS^2^*m/z*	MS^2^ aglycone's annotation	Error (ppm)	Neutral loss (Da)	Candidate bond type and sugar derivative moiety
2.18	563.1420	Schaftoside or isoschaftoside (apigenin di-*C*-glycosides)	2.43	353.0673	Apigenin-di-etenol [M − H]^−^	1.76	210.075 (90 Da + 120 Da)	Di-*C*-hexoside or *C*-hexoside-*C*-pentoside
2.55	463.0869	Hyperin (quercetin 3-*O*-d-galactoside); isoquercitrin (quercetin 3-*O*-d-glucoside); quercimeritrin (quercetin 7-*O*-d-glucoside); 7-methoxyquercetin-3-*O*-xylopyranose; Quercetin-3-*O*-allopyranoside; myricetrin (myricetin 3-*O*-rhamnoside)	−2.81	301.0319	Quercetin [M − H]^−^	−1.17	162.055	*O*-Hexose
2.75	433.0764	Reynoutrin (quercetin 3-*O*-xyloside); guajaverin (quercetin-3-*O*-arabinopyranoside)	−2.77	300.0266	Quercetin [M − H]˙^−^	−3.17	133.049	*O*-Pentose
2.85	563.1426	Schaftoside or isoschaftoside (apigenin di-*C*-glycosides)	3.50	284.0321	Kaempferol [M − H]˙^−^	−1.90	279.111	*O*-Deoxyhexosyl-pentose
2.87	447.0927	Astragalin (kaempferol 3-*O*-glucoside); quercitrin (quercetin 3-*O*-rhamnoside); orientin (luteolin 8-*C*-glucoside) or isoorientin (luteolin 6-*C*-glucoside)	−1.34	300.0272	Quercetin [M − H]˙^−^	−1.17	147.066	*O*-Deoxyhexose
4.43	577.1346	Procyanidin B1; procyanidin B3; proanthocyanidin; kaempferol-3-*O*-(4′′-*p*-coumaroyl)-rhamnoside	−0.87	285.0383	Kaempferol [M − H]^−^	−7.58	292.097	*O*-Coumaroyl-deoxyhexose
5.57	723.1727	Kaempferol 3-(2′′,4′′-di-*p*-coumaroylrhamnoside)	1.11	285.0383	Kaempferol [M − H]^−^	−7.58	438.134	*O*-Di-coumaroyl-deoxyhexose

The reporting of glycosylated flavonoid compounds, as detailed in Table 1, is arguably more precise, particularly given the complexities associated with predicting sugar attachment sites. Under highly standardized and outlined conditions, and using mass spectrometry fragmentation rules, intensity ratios of flavonoid fragments can indicate the substitution position.^3^ However, simply automated spectral similarity approaches, often used in literature for annotating glycosylated flavonoids and specifying both the sugar type and its position (*e.g.* Astragalin = Kaempferol 3-*O*-β-d-glucose), may not always be accurate. Factors like collision type (*e.g.* CID, collision-induced dissociation, and HCD, higher energy collisional dissociation) and variations in collision energies can significantly influence the fragment distribution within MS^2^ spectra, potentially leading to misinterpretations.^94,95^

Given that public MS^2^ repositories, such as GNPS, pursue a large diversity of spectra acquired in different instruments under different conditions, it is crucial to manually inspect the automated hits concerning the instrument and collision energy. For instance, we compared MS^2^ spectra of astragalin from GNPS libraries, acquired under different conditions (two spectra acquired using an QTOF analyzer in negative mode, and two acquired using an Orbitrap analyzer in positive mode) (ESI 7 – Fig. S15†). Despite not specifying the collision energies of each data, the results showed quantitative and qualitative variance among the fragments. The qualitative variance was especially for Orbitrap MS data, demonstrating the potential for misinterpretation in the automated annotation of glycosylated flavonoids, once the sugar moiety position cannot be precisely assured based only on gas phase fragmentation reactions.

Furthermore, the lack of specified collision energies and standardization across the MS data repositories remains a challenge in metabolite annotation. Considering these findings, we advocate for reporting these NPs by indicating the aglycone, the glycosidic bond type, and the sugar type (*e.g.*, Kaempferol *O*-hexoside) – information that is readily accessible through standard LC-MS methods. This approach ensures a more reliable and consistent annotation of glycosylated flavonoids, circumventing the uncertainties associated with automated spectral similarity techniques.

The annotation of compounds *via* MS^2^ spectral matching is typically classified as level 2 of confidence according to the MSI guidelines, denoting putatively annotated compounds (*e.g.* without chemical reference standards, based upon physicochemical properties and/or spectral similarity with spectral libraries).^71^ Herein, our annotations are reported with level 3 of confidence, indicating putatively characterized compound classes, however including precise flavonoid aglycone and sugar types. This is due to matching monoisotopic masses with phylogenetic-related databases could further yield more reliable level 3 annotations, minimizing the risk of unrelated and false positive hits.

In summary, this semi-automated approach for MS data analysis allows detailed DIA-based manual inspection. This was designed to overcome some of the primary barriers in confident metabolite annotation within plant metabolomics studies. This method provides an alternative approach in response to the current limitations, which include for instance the scarcity of sample-related MS^2^ spectral databases and recurrent annotation of unrelated hits. We underscore the critical role of the analyst's expertise in conjunction with the known semi-automated processes, enabling the extraction of maximal relevant information from the samples under the same analysis. Fully automated and not manually inspected analyses often contain errors that are overlooked in peer reviews.^52^ Therefore, the dual approach of manual inspection and automated processes ensures a more rigorous and accurate interpretation of MS data.

### 3.1 *Ocotea* spp. applicability case: literature comparison

Data on flavonoid profiles in *Ocotea* species remain scarce in existing literature, with only 3.4% of *Ocotea* species reported as flavonoid producers in the latest review from 2020.^15^ Despite that, biosynthesis investigation studies can provide valuable insights into plant evolution, linking botanical diversification to genetic alterations, which are associated with the evolutionary and adaptive processes of the *Ocotea*. In recognised *Ocotea* spp., flavonoids predominantly include glycosylated derivatives of catechin, epicatechin, quercetin, and kaempferol, with 45.5% being *O*-glycosylated. For the six *Ocotea* spp. present in this study, it was indicated an even larger proportion of the *O-*glycosylated derivatives of 60.1%. That suggests that these species may belong to a basal stage in Lauraceae's evolutionary lineage. Moreover, according to the literature, it is the genus's flavone-to-flavonol ratio of 0.05 that supports its ancestral role in the Lauraceae, as proposed by previous botanical and phylogenetic studies.^15,96^ Although not representative of the genus (*n* = 6), the flavonoid profiling in this research has revealed new aspects of this tendency, where the overall flavone-to-flavonol ratio of the studied species was 0.48 (60 hits from flavone, and 125 hits from flavonol). This ratio was not balanced among the species (*O. odorifera* = 0.76, *O. diospyrifolia* = 0.24, *O. guianensis* = 0.19, *O. lancifolia* = 0.04, *O.notata* = 0.0, and *O. porosa* = 0.80), suggesting that perhaps *O. odorifera* and *O. porosa* are not as evolutionarily close as the others in the Lauraceae family, when compared to previous research in the literature.^15^

The significant presence of flavones, which has led to a higher flavone-to-flavonol ratio, could suggest a more evolved phylogenetic position. This deviation in flavonoid composition across the species points towards a more complex evolutionary pathway within the *Ocotea* genus and could be indicative of the presence of diverse evolutionary paths. Even though, the less annotated *O*-alkylated flavonoids in *Ocotea* spp. in literature, and also in our results, might further support this antiquity proposal of the genus.^15^ However, as evolutionary analyses become more robust with larger sets of species, the precision of characterization analyses can also improve with advancements in analytical technology. Therefore, as these areas develop, the number of identified *Ocotea* flavonoid producers is likely to increase, offering a more comprehensive understanding of their evolutionary journey. Still, as an overview, the results suggest that flavonoid profiles might play a role in tracing the evolutionary position of *Ocotea* spp. within the Lauraceae family.

In this study, we significantly expanded the detection and annotation of glycosylated flavonoids in six *Ocotea* species, surpassing previous metabolomics and phytochemical counts in the literature. More specifically, only a few flavonoids were reported for these species: *O. diospyrifolia* (only 2),^13^*O. guianensis* (6),^54^*O. lancifolia* (9),^61^*O. notata* (12),^55,60,62^*O. odorifera* (11),^57^ and *O. porosa* (only 3),^97^ and our study annotated more than 50 flavonoid backbones for each species (ESI 7 – Table S5†). Except for *O. notata*, apigenin fragment backbones have been identified in the five other *Ocotea* species. This finding aligns with previous reports of apigenin flavonoids in *O. odorifera*. However, for the remaining four species, this is the first report identifying the presence of a flavone backbone. Additionally, this study also represents the first report of flavonol kaempferol and quercetin in *O. porosa* species.

**Fig. 5:**
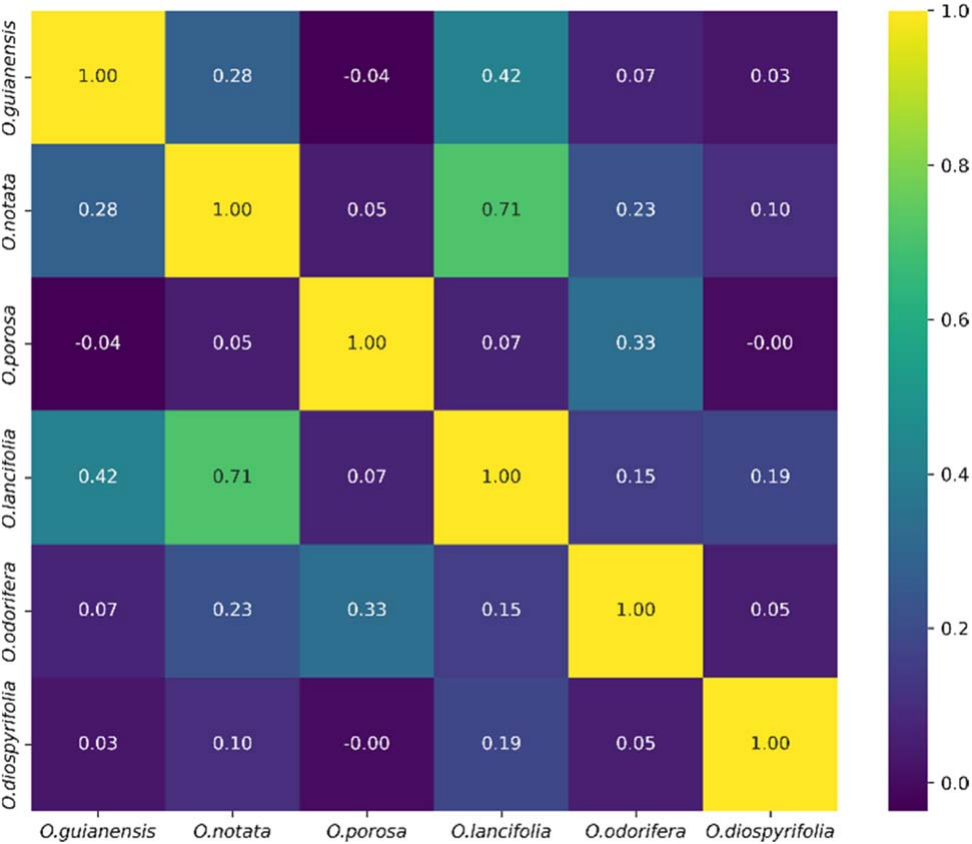
Heatmap correlation of feature profiles from the 6 *Ocotea* species.

**Fig. 6:**
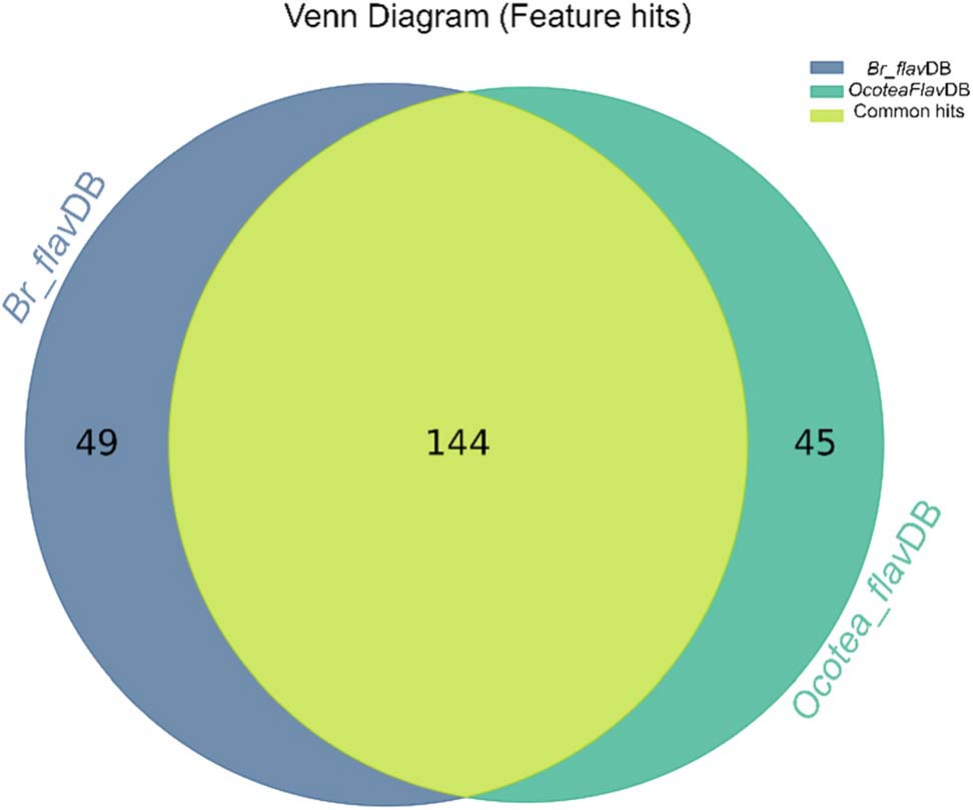
Venn Diagram of *Br_flavDB* and *Ocotea*_*flavDB* hits. This diagram illustrates the unique and shared feature hits between these two in-house databases.

The heatmap correlation analysis showed that *O. lancifolia* and *O. notata* have very similar feature profiles, whereas *O. porosa* and *O. guianensis* demonstrated to be quite distinct (Fig. 5). In addition, the comparative analysis between *Br_flavDB* and *Ocotea*_flavDB hits was concisely visualized through a Venn diagram, revealing the intersection and uniqueness of the feature hits across the datasets. The diagram delineated 49 *m/z* hits that were exclusive to *Br_flavDB*, illustrated by the distinct left circle. These hits suggest the presence of new flavonoids in these plants, not yet isolated for species from the *Ocotea* genus, although they are present in other plant genera from Brazilian flora. Conversely, *Ocotea_flavDB* exhibited exclusivity in 45 unique *m/z* hits, as represented by the right circle in the Venn diagram. This supports the fact that manual curation is essential for enhanced in-house database constructions, as *Ocotea_flavDB* contains online database entries but also highly curated literature data, including all published articles in the literature for the *Ocotea* genus. In addition, a significant overlap between the two databases hits, with 144 unique *m/z* is depicted in the intersecting zone (Fig. 6), which was expected to be the largest section, as the online NuBBE_DB_ data is present in both in-house databases.

**Fig. 7:**
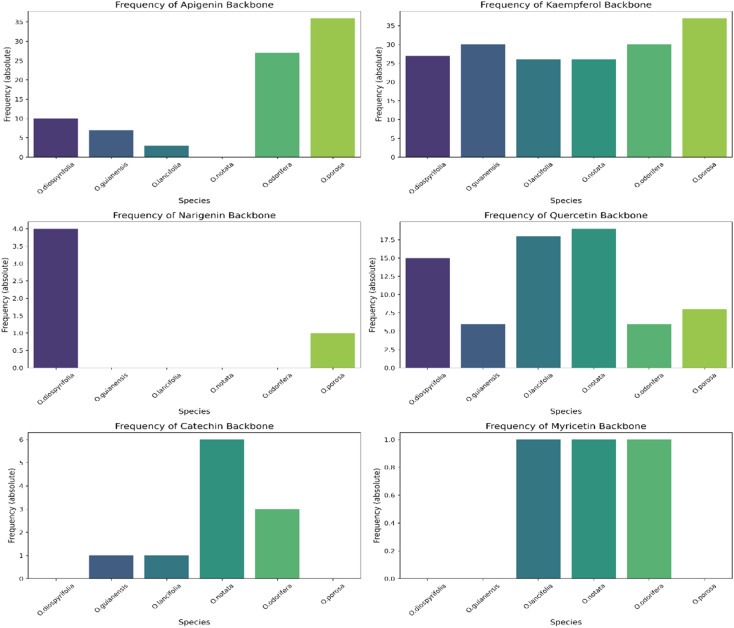
Distribution of flavonoid types across *Ocotea* species. This figure presents individual bar graphs showing the distribution of flavonoid types: flavone, flavonol, flavanonol, and flavan-3-ol, among each examined species within the *Ocotea* genus. The bars represent the absolute frequency distribution of each flavonoid type, illustrating their prevalence or absence among the species.

We annotated common flavonoid backbones such as apigenin, kaempferol, and quercetin across most species. Notably, myricetin was exclusive to *O. lancifolia*, *O. notata,* and *O. odorifera*, while narigenin was unique to *O. diospyrifolia* and *O. porosa*. We observed that the flavonol backbones, kaempferol, and quercetin, predominantly exhibited *O*-glycosylation patterns, while apigenin derivatives were found mainly as *C*-glycosides. Interestingly, myricetin backbones were detected for the first time in these mentioned species. However, we highlight that as myricetin backbone was not annotated in its pure aglycone form (*m/z* [M − H]^−^ = 317.0303), unlike kaempferol, quercetin, and apigenin. Also, it was only annotated as *C*-glycoside fragments. This is particularly intriguing because the genus *Ocotea* is not known in the literature as a producer of myricetin derivatives, in corroboration with our study, which only recorded a few myricetin MS^1^ hits among the species. In addition, the *C*-glycoside myricetin derivatives are not widespread in plants. Consequently, while this suggests the possibility of new flavonoids in these species, there is also a potential for false positives. Further in-depth chemical investigation is therefore essential to resolve this issue and potentially confirm the presence of these flavonoids. Regarding the primary flavonoid types: flavone, flavonol, and flavanonol were commonly found across most species. Flavan-3-ol is found in all except *O. diospyrifolia* and *O. porosa*. Thus, our comparative analysis (Fig. 7) has been designed to illustrate the distribution of these chemical components across different species, offering more explicit insights into the chemical diversity of backbones and flavonoid types within the *Ocotea* genus.

Despite the high complexity of LC-HRMS/DIA data, our strategy enabled a comprehensive yet straightforward analysis of *Ocotea* plant extracts, bypassing exhaustive isolation and characterization processes. DIA-MS, though less commonly used than DDA, is highly valuable for metabolomics studies, particularly for annotation-based metabolic profiling due to its extensive metabolite and fragment coverage.^35,39,48^ Our results were revealing, particularly in discerning the distinct aglycone patterns across different plant species, further enhanced by analyses of gas-phase fragmentation reactions. Moreover, with the development of specialized in-house databases, and the use of advanced computational tools, we have achieved reliable annotation and comprehensive flavonoid profiling. These tools can predict possible glycosylation patterns, aiding in the interpretation of MS flavonoid data. In summary, while traditional techniques face challenges in the analysis of glycosylated flavonoids, ongoing advancements in analytical methods and computational tools are continually improving the detection and annotation of these complex and widespread molecules in complex NP sources.

Therefore, rather than attempting precise annotations of complex glycosylated flavonoids, a more generalized method would involve identifying the aglycone, the type of glycosidic bond, and the sugar type (such as “Kaempferol-*O*-hexoside”). These elements are more easily reproduced using standard LC-MS techniques. Thus, it offers a pragmatic and reliable way to report glycosylated flavonoids in NP research. Adopting this strategy could enhance the accuracy and utility of profiling flavonoid data in the field of metabolomics.

## 4 Conclusion

This study not only speeds up the way for the annotation process but also significantly improves the accuracy and coverage for identifying the correct aglycones, sugar moieties, and low-abundance glycosylated flavonoid types. Despite certain challenges in MS^2^ spectra deconvolution in MZmine 3, our findings underscore the immense potential of LC-MS/DIA in revolutionizing metabolite profiling in NP research. By combining cutting-edge technological platforms with expert manual scrutiny, we have demonstrated a powerful strategy that can be readily implemented in NP research. This approach is crucial for advancing our understanding and annotation of complex and varied glycosylated flavonoids. As our applicability case has shown, the *Ocotea* plants might produce an extensive profile of glycosylated flavonoids, which could be potentially useful in chemosystematics studies due to their distinct patterns. Still, in the present study, the apigenin flavone backbone was reported for most of the investigated *Ocotea* species, an unprecedented finding in the literature. The flavonols kaempferol and quercetin were mainly found in the *O-*glycosides form, while flavone apigenin derivatives were in the *C*-glycosides form. Interestingly, *O. porosa* displayed the highest flavone : flavonol ratio of 0.8, and thus distinct from the normally expected ratio for the *Ocotea* plant species. Besides those interesting results, our study also sheds light on the best practices for reporting such compounds, prompting a revaluation of reporting standards of specific classes, such as glycosylated flavonoids. Ultimately, this integrated approach paves the way for more comprehensive and reliable metabolite annotation in complex NP sources using LC-MS/DIA data.

Future research should aim to extend this profiling approach to a wider range of species within the *Ocotea* genus and other complex plant matrices. Further development of more inclusive and detailed metabolite databases would also enhance the applicability of this methodology across different fields of NP research. Moreover, exploring the biological activities of the newly identified flavonoids could provide valuable insights into their potential health benefits and pharmacological applications. This study sets a new benchmark for flavonoid profiling in complex natural matrices, offering valuable methodologies and insights for researchers in the fields of metabolomics, analytical chemistry, and NP chemistry at large.

## Data availability

The *Ocotea* datasets of the current study were deposited to the Mass Spectrometry Interactive Virtual Environment (MassIVE) repository. The data that support the findings of this study are available under the registry code MSV000094030. Guidelines for files submitted to MassIVE for public access can be found online (https://massive.ucsd.edu/ProteoSAFe/static/massive.jsp). The MZmine processing parameters, and the in-house databases (.csv files), MZmine batch file (.xml), and the structures of aglycone fragments and sugar neutral losses (.cdx file) are available at Zenodo open repository (https://doi.org/10.5281/zenodo.10810967). Accompanying information (ESI 7 and video†) supplements this paper online. Additional files to download are available at Zenodo's link https://doi.org/10.5281/zenodo.10810967.

## Author contributions

A. K. N., A. C. C. P. L., D. A. C. P., M. F. A., and P. C. P. B. conceived the idea. A. K. N. and M. F. A. wrote the draft paper. M. M. performed the high-resolution DIA-MS^*E*^ experiments for data acquisition. M. F. A. performed MS data conversion and data processing. A. K. N. and M. S. F. constructed the KNIME workflows. A. K. N., D. A. C. P., F. C. N., M. F. A., P. C. P. B., R. C. contributed to data interpretation. A. K. N., D. F. D., F. C. N., M. F. A., M. G. S. aided in gas-phase fragmentation reactions elucidation. All authors participated in the discussion and revision of the manuscript.

## Conflicts of interest

There are no known conflicts to declare.

## Supplementary Material

RA-014-D4RA01384K-s001

RA-014-D4RA01384K-s002
