# Supplementary material for: LC-MS/DIA-based strategy for comprehensive flavonoid profiling: an Ocotea spp. applicability case
Source: RSC Adv. 2024 Apr 2;14(15):10481–98. doi: 10.1039/d4ra01384k (PMC10985591; doi:10.1039/d4ra01384k)
Supplement: RA-014-D4RA01384K-s001 [file RA-014-D4RA01384K-s001.pdf]

Electronic Supplementary Material (ESI) for RSC Advances.
This journal is © The Royal Society of Chemistry 2024

Supplementary material

LC-MS/DIA-based strategy for comprehensive flavonoid profiling: An *Ocotea* spp. applicability case

Matheus Fernandes Alves[a], Albert Katchborian Neto[a], Paula Carolina Pires Bueno[b], Fausto Carnevale-Neto[c], Rosana Casoti[d], Miller Santos Ferreira[a], Michael Murgu[e], Ana Claudia Chagas de Paula Ladvocat[f], Danielle Ferreira Dias[a], Marisi Gomes Soares[a] and Daniela Aparecida Chagas de Paula[a]*

[a]*Institute of Chemistry, Federal University of Alfenas-MG, 37130-001, Alfenas, Minas Gerais, Brazil*
[b]*Leibniz Institute of Vegetable and Ornamental Crops (IGZ), Theodor-Echtermeyer-Weg 1, 14979, Großbeeren, Germany*
[c]*Northwest Metabolomics Research Center, Department of Anesthesiology and Pain Medicine, University of Washington, 850 Republican Street, Seattle, Washington 98109, United States*
[d]*Antibiotics Department, Federal University of Pernambuco, 50670-901, Recife, Pernambuco, Brazil*
[e]*Waters Corporation, Alameda Tocantins 125, Alphaville, 06455-020, São Paulo, São Paulo, Brazil*
[f]*Faculty of Pharmacy, Federal University of Juiz de Fora, 36036-900, Juiz de Fora, Minas Gerais, Brazil*

Figures

Figure S1. KNIME workflow for database construction..2

Figure S2. UPLC-HRMS/DIA MS[1] BPI chromatogram from *O. diospyrifolia* leaf extract in negative mode.......2

Figure S3. UPLC-HRMS/DIA MS[2] BPI chromatogram from *O. diospyrifolia* leaf extract in negative mode.......2

Figure S4. UPLC-HRMS/DIA MS[1] BPI chromatogram from *O. porosa* leaf extract in negative mode..............3

Figure S5. UPLC-HRMS/DIA MS[2] BPI chromatogram from *O. porosa* leaf extract in negative mode..............3

Figure S6. UPLC-HRMS/DIA MS[1] BPI chromatogram from *O. lancifolia* leaf extract in negative mode...........4

Figure S7. UPLC-HRMS/DIA MS[2] BPI chromatogram from *O. lancifolia* leaf extract in negative mode...........4

Figure S8. UPLC-HRMS/DIA MS[1] BPI chromatogram from *O. odorifera* leaf extract in negative mode...........5

Figure S9. UPLC-HRMS/DIA MS[2] BPI chromatogram from *O. odorifera* leaf extract in negative mode...........5

Figure S10. UPLC-HRMS/DIA MS[1] BPI chromatogram from *O. notata* leaf extract in negative mode.............6

Figure S11. UPLC-HRMS/DIA MS[2] BPI chromatogram from *O. notata* leaf extract in negative mode.............6

Figure S12. UPLC-HRMS/DIA MS[1] BPI chromatogram from *O. guianensis* leaf extract in negative mode.......7

Figure S13. UPLC-HRMS/DIA MS[2] BPI chromatogram from *O. guianensis* leaf extract in negative mode.......7

Figure S14. *FlavonoidSearch* screen evidencing MS[2] scores for kaempferol and datiscetin............................8

Figure S15. Astragalin MS[2] spectra from GNPS..8

Tables

Table S1. Level 3 annotation based on MS[1] hits from *Ocotea_flavDB*...9

Table S2. Level 3 annotation based on MS[1] hits from *Br_flavDB*...35

Table S3. Common sugar types and neutral fragments 62

Table S4. Aglycone annotation based on MS[2] hits from *FlavAgly_DB* 65

Table S5. Annotation numbers for MS[1]- and MS[2]-spectra level of *Ocotea* species 77

Figures

Figure S1. KNIME workflow for database construction

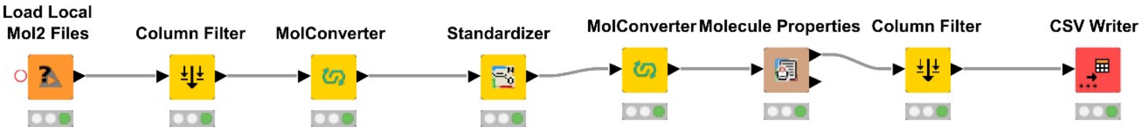

Figure S2. UPLC-HRMS/DIA MS[1] BPI chromatogram from *O. diospyrifolia* leaf extract in negative mode

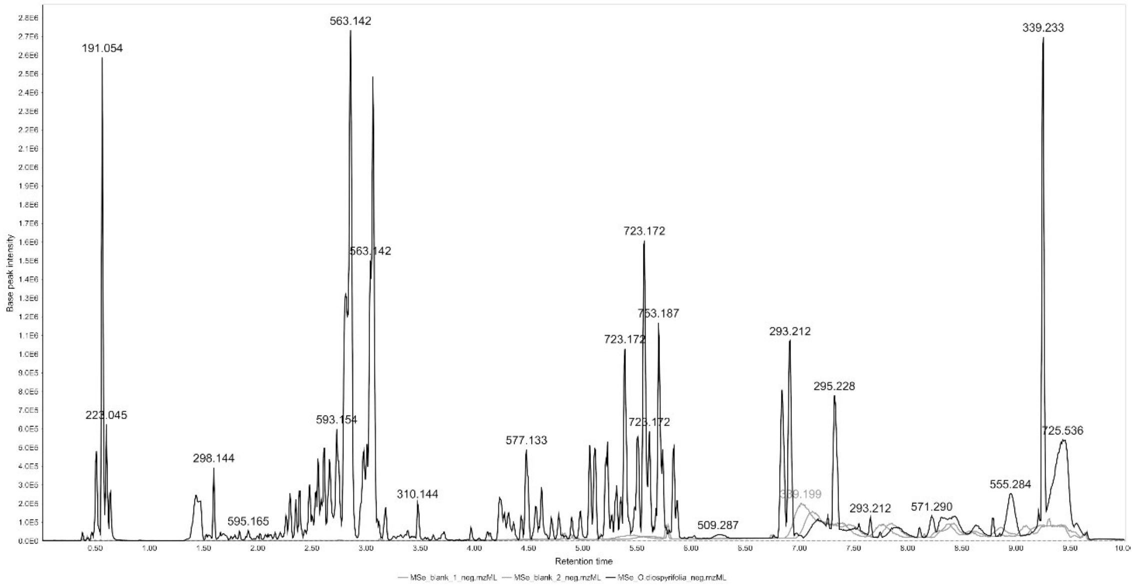

Figure S3. UPLC-HRMS/DIA MS[2] BPI chromatogram from *O. diospyrifolia* leaf extract in negative mode

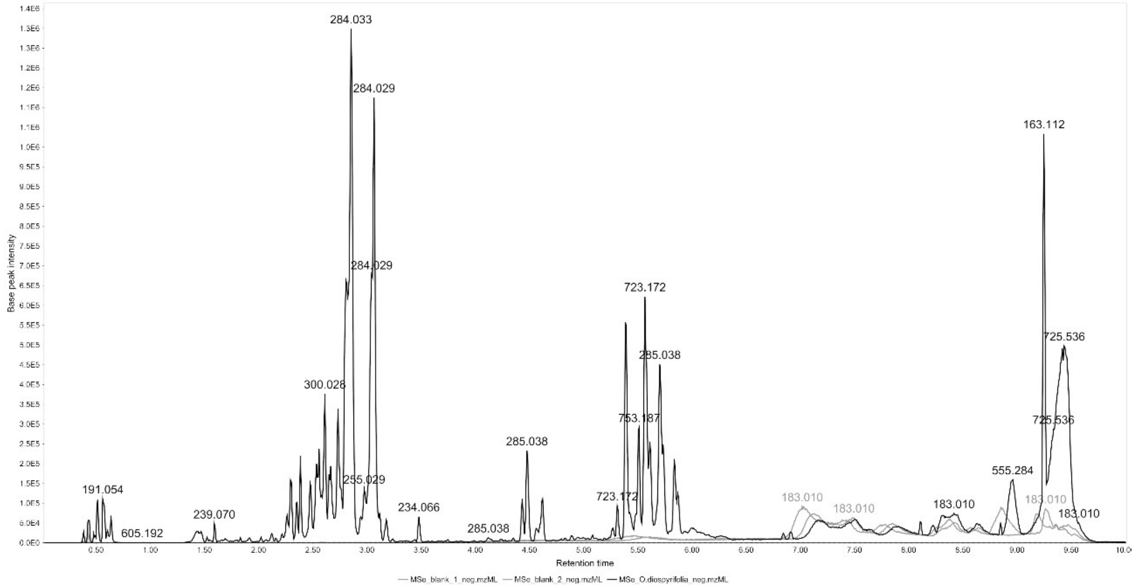

Figure S4. UPLC-HRMS/DIA MS[1] BPI chromatogram from *O. porosa* leaf extract in negative mode

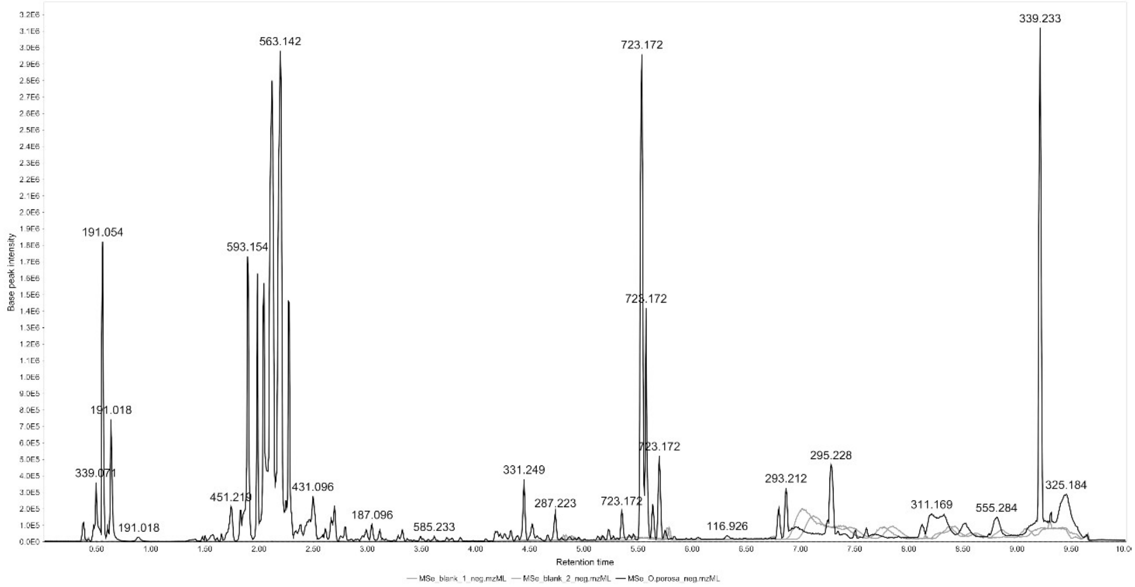

Figure S5. UPLC-HRMS/DIA MS[2] BPI chromatogram from *O. porosa* leaf extract in negative mode

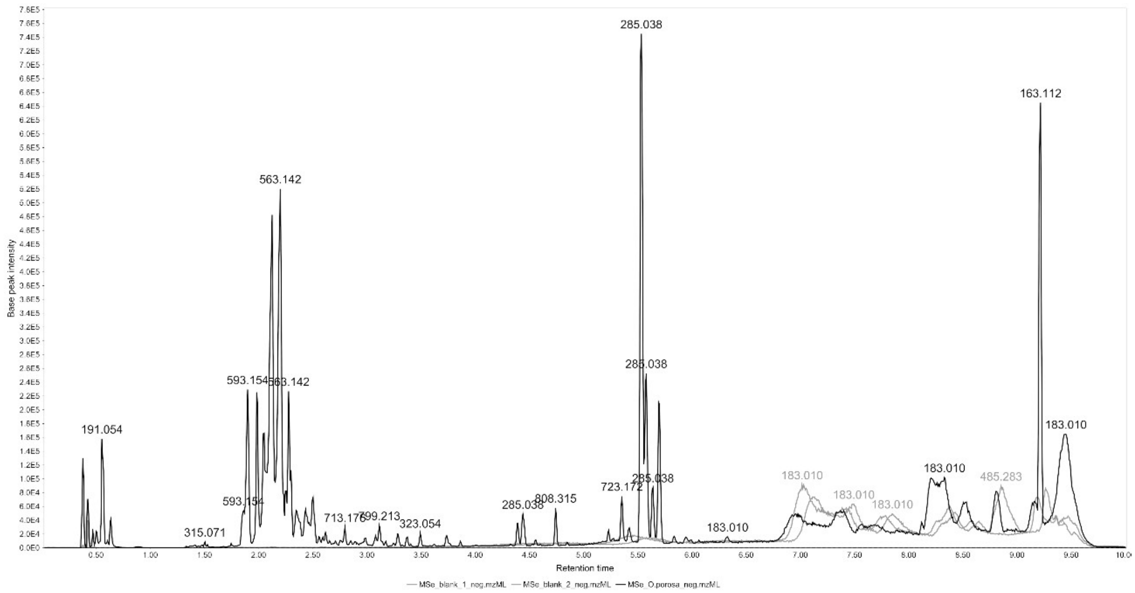

Figure S6. UPLC-HRMS/DIA MS[1] BPI chromatogram from *O. lancifolia* leaf extract in negative mode

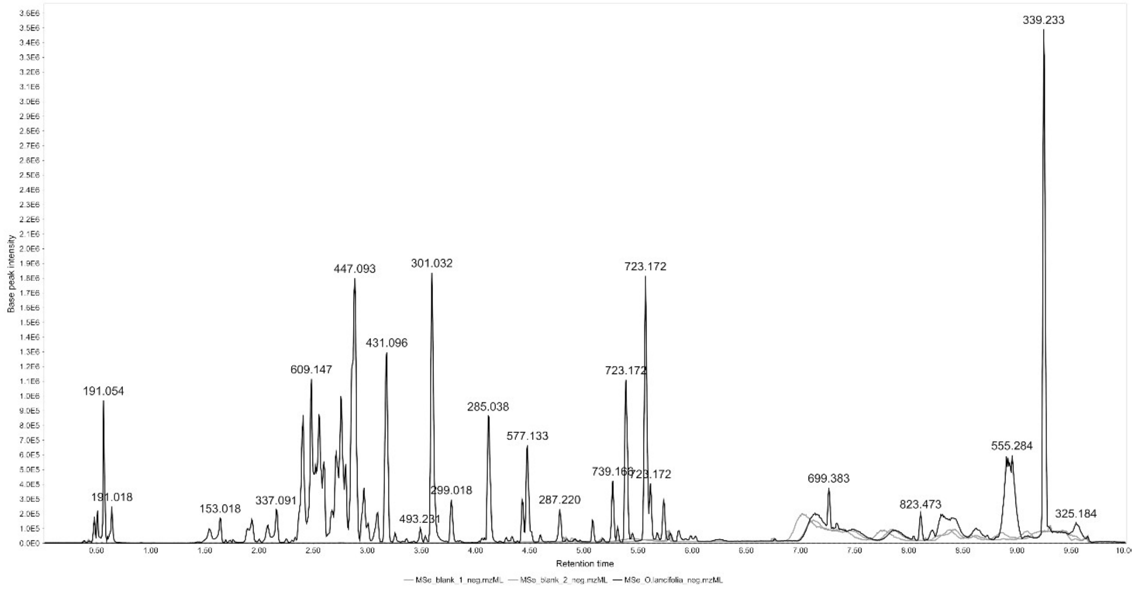

Figure S7. UPLC-HRMS/DIA MS[2] BPI chromatogram from *O. lancifolia* leaf extract in negative mode

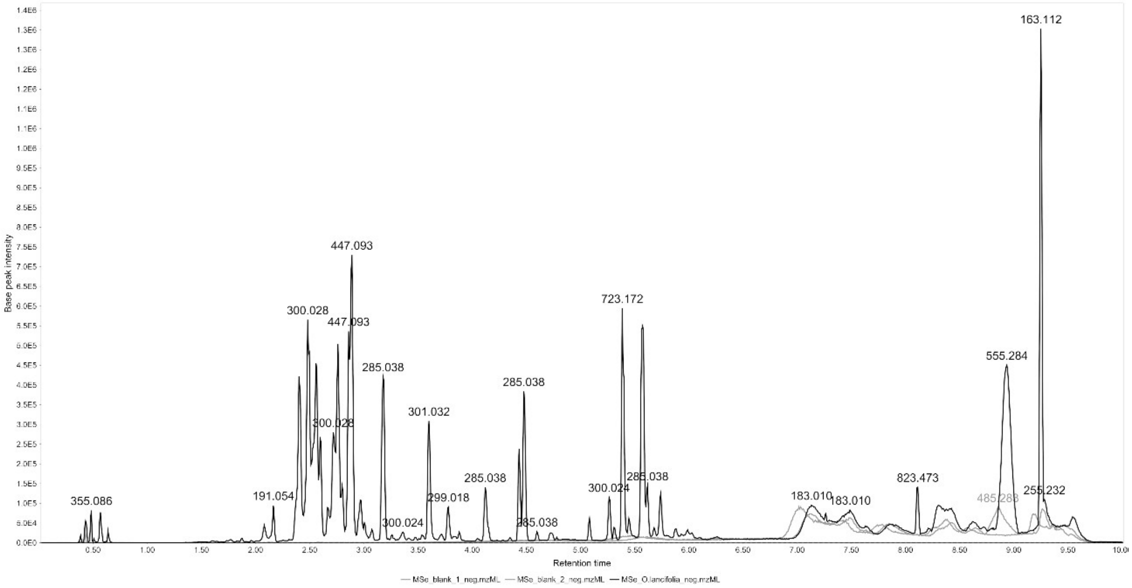

Figure S8. UPLC-HRMS/DIA MS[1] BPI chromatogram from *O. odorifera* leaf extract in negative mode

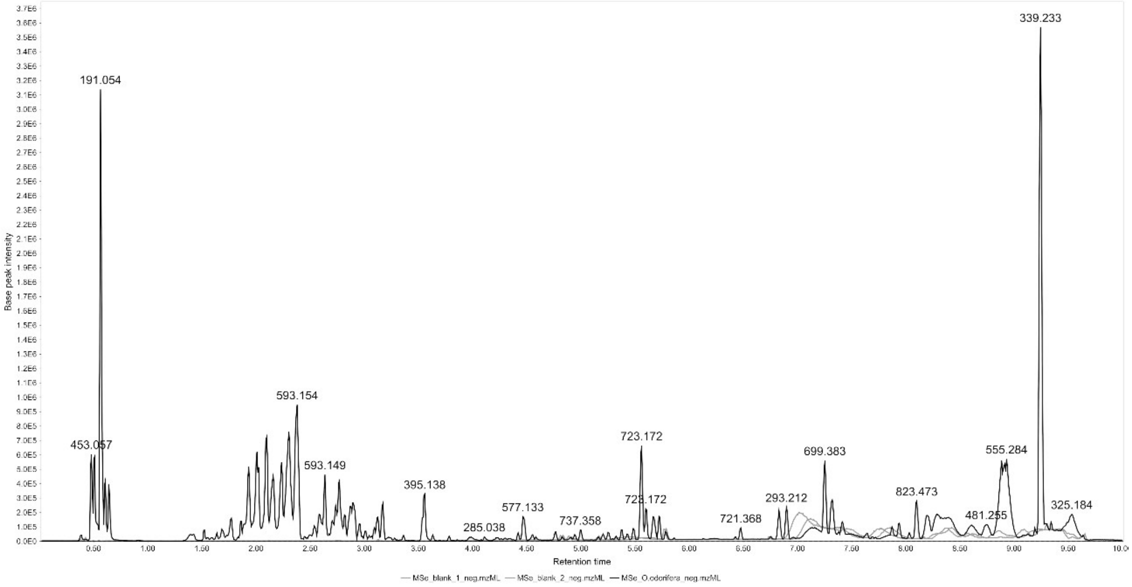

Figure S9. UPLC-HRMS/DIA MS[2] BPI chromatogram from *O. odorifera* leaf extract in negative mode

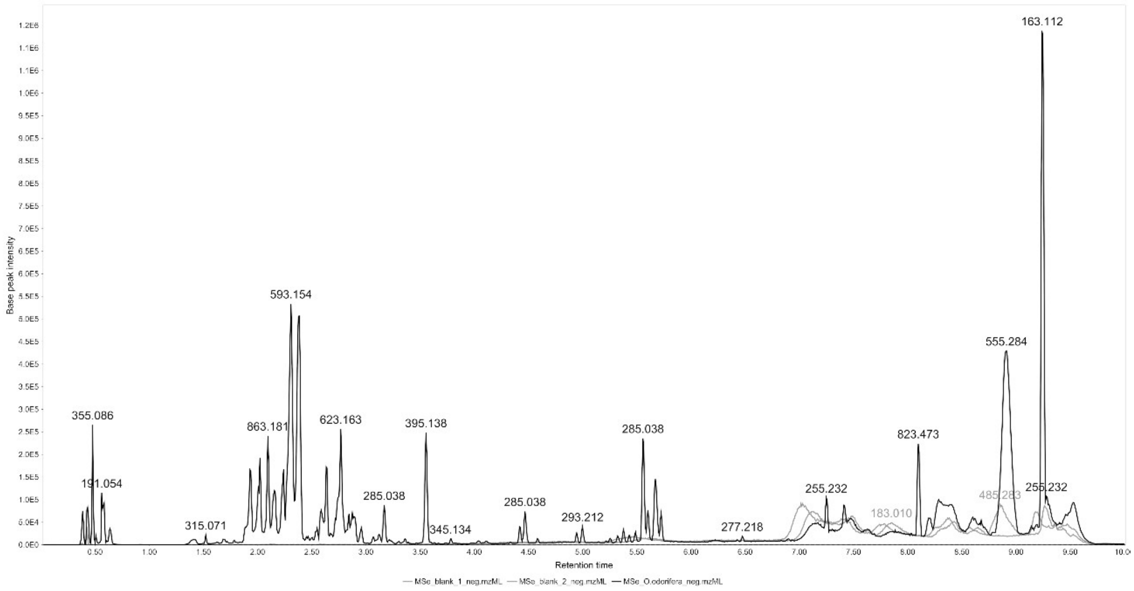

Figure S10. UPLC-HRMS/DIA MS[1] BPI chromatogram from *O. notata* leaf extract in negative mode

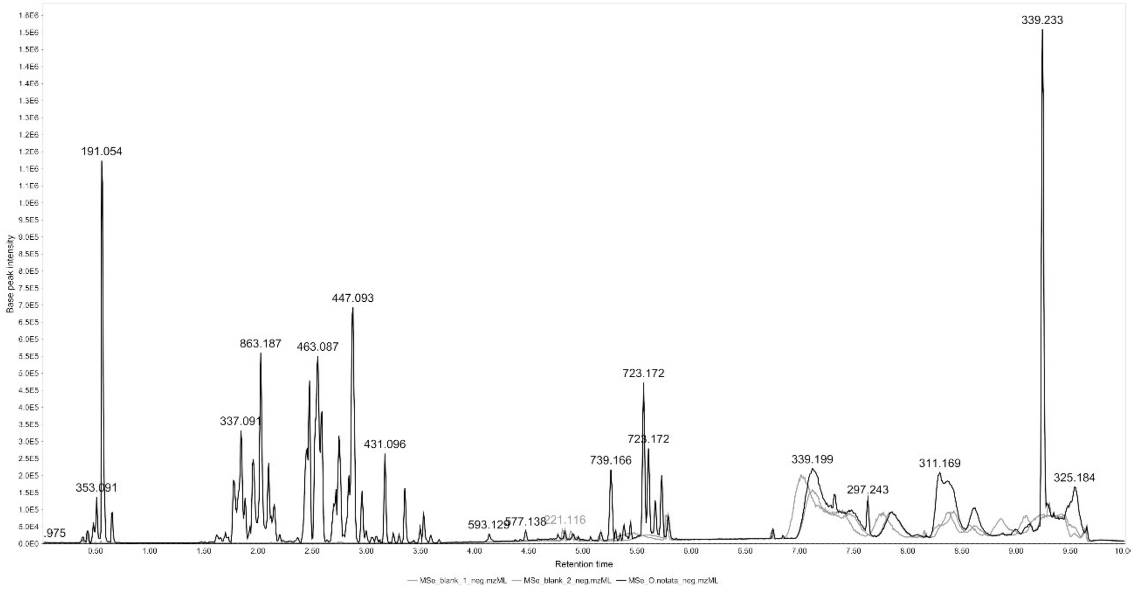

Figure S11. UPLC-HRMS/DIA MS[2] BPI chromatogram from *O. notata* leaf extract in negative mode

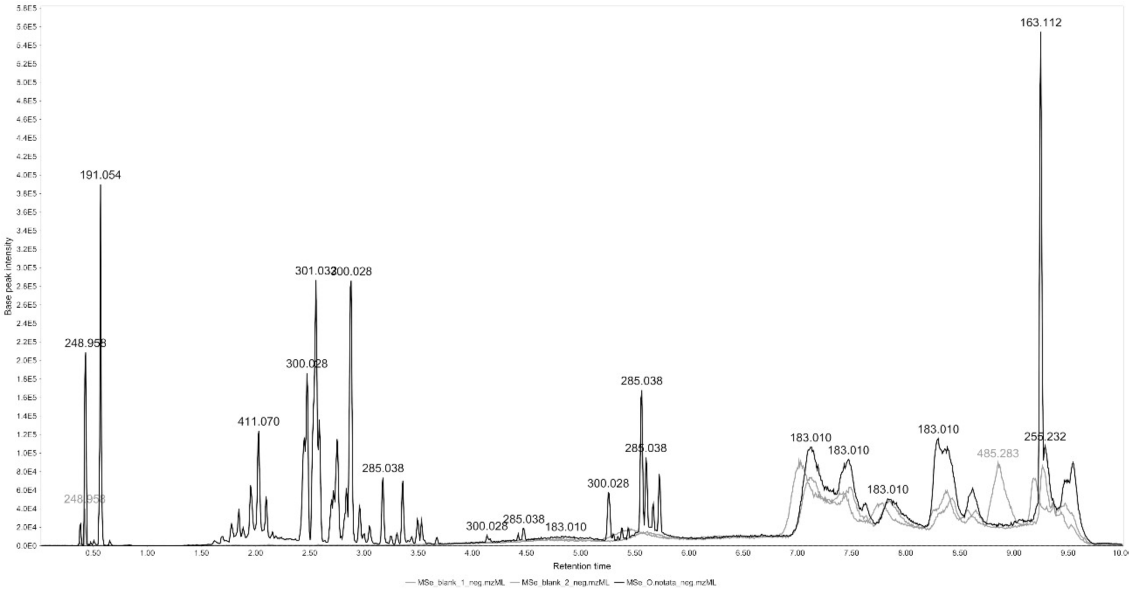

Figure S12. UPLC-HRMS/DIA MS^1 BPI chromatogram from *O. guianensis* leaf extract in negative mode

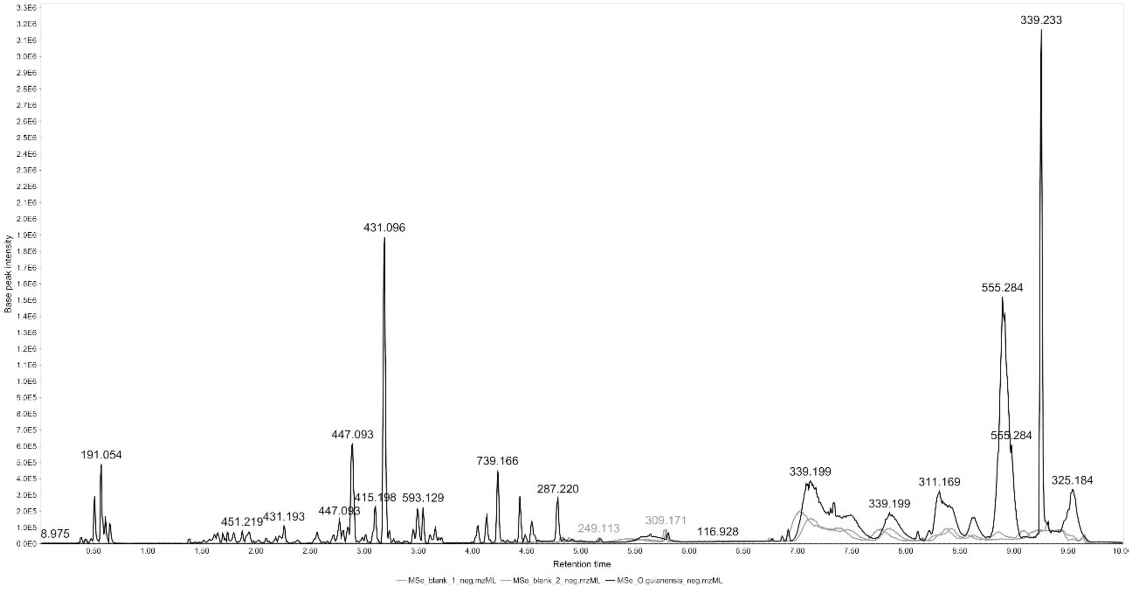

Figure S13. UPLC-HRMS/DIA MS^2 BPI chromatogram from *O. guianensis* leaf extract in negative mode

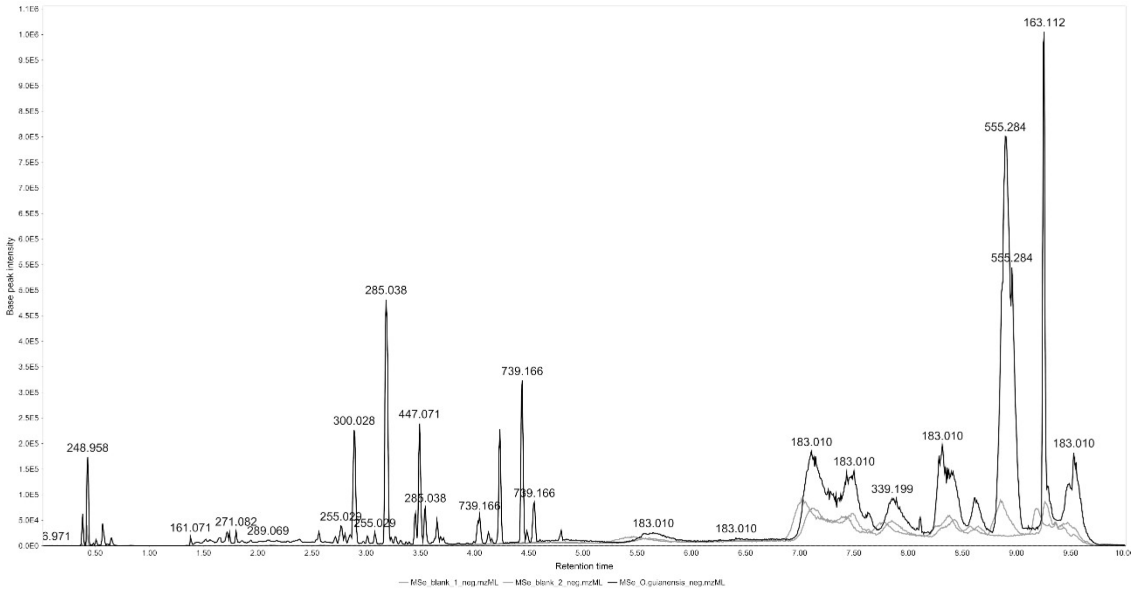

Figure S14. *FlavonoidSearch* screen evidencing MS^2 scores for kaempferol and datiscetin

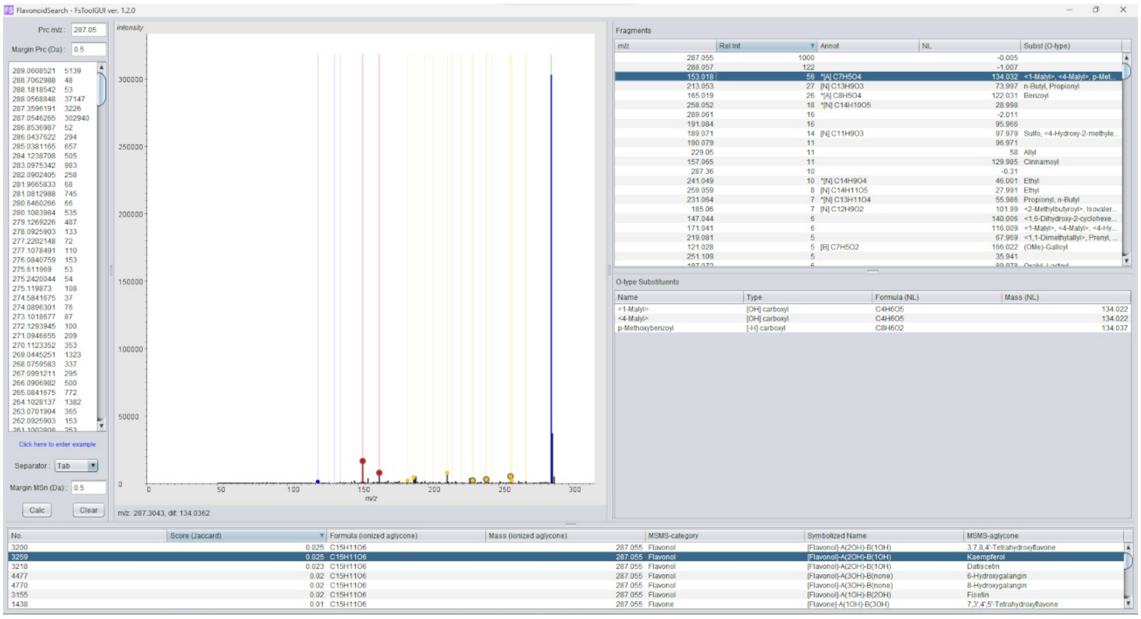

Figure S15. Astragalin MS^2 spectra from GNPS

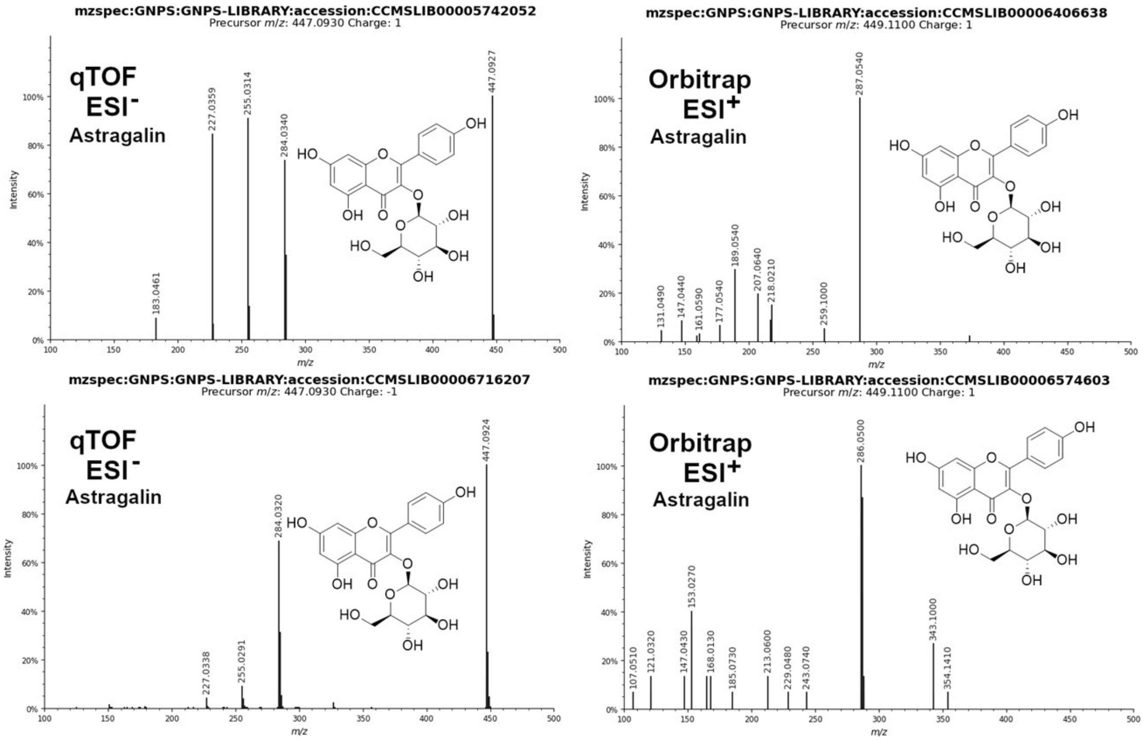

Tables

Table S1. Level 3 annotation based on MS^1 hits from *Ocotea_flavDB*

N°	*m/z*	RT (min)	*Ocotea_flavDB* annotations (Name: adduct: Mzmine score)	Error (ppm)	Molecular formula	MS^1 blank replicates Peak area		MS^1 *O.guianensis* Peak area	MS^1 *O.notata* Peak area	MS^1 *O.porosa* Peak area	MS^1 *O.lancifolia* Peak area	MS^1 *O.odorifera* Peak area	MS^1 *O.diospyrifolia* Peak area
1	577.1350	1.62	Procyanidin B1: [M-H]-: 0.564;Kaempferol 3-4''-*p*-coumarylrhamnoside: [M-H]-: 0.564	-0.3	C30H26O12	-	-	6.61E+02	8.29E+02	-	-	-	-
2	575.1205	1.63	(Epi)-Catechin dimer: [M-H]-: 0.488	1.7	C30H24O12	-	-	-	1.72E+02	-	-	4.42E+02	-
	575.1205	1.63	Dihydrokaempferol (Aromadendrol): [2M-H]-: 0.488	1.7	C15H12O6	-	-	-	1.72E+02	-	-	4.42E+02	-
3	577.1351	1.70	Procyanidin B1: [M-H]-: 0.582;Kaempferol 3-4''-*p*-coumarylrhamnoside: [M-H]-: 0.582	-0.1	C30H26O12	-	-	7.15E+02	1.02E+03	-	5.82E+00	5.35E+00	-
4	575.1199	1.71	(Epi)-Catechin dimer: [M-H]-: 0.553	0.6	C30H24O12	-	-	3.41E+01	2.03E+02	-	-	3.62E+02	-
	575.1199	1.71	Dihydrokaempferol (Aromadendrol): [2M-H]-: 0.553	0.6	C15H12O6	-	-	3.41E+01	2.03E+02	-	-	3.62E+02	-
5	289.0706	1.79	(+)-catechin: [M-H]-: 0.482;(-)-Epicatechin: [M-H]-: 0.482	-3.9	C15H14O6	-	-	1.42E+02	3.85E+02	-	1.23E+01	-	-
6	575.1200	1.81	(Epi)-Catechin dimer: [M-H]-: 0.541	0.9	C30H24O12	-	-	3.49E+01	4.08E+02	-	-	4.47E+01	-
	575.1200	1.81	Dihydrokaempferol (Aromadendrol): [2M-H]-	0.9	C15H12O6	-	-	3.49E+01	4.08E+02	-	-	4.47E+01	-

			: 0.541										
7	577.1351	1.82	Procyanidin B1: [M-H]-: 0.590;Kaempferol 3-4''-*p*-coumarylrhamnoside: [M-H]-: 0.590	0.0	C30H26O12	-	-	3.97E+02	2.47E+02	-	5.94E+01	2.35E+02	-
8	609.1470	1.82	Schaftoside (Apigenin 6-*C*-hexoside-8-*C*-pentoside): [M+FA]-: 0.499	1.5	C26H28O14	-	-	-	-	4.42E+02	-	-	-
9	463.0870	1.84	Quercetin-3-*O*-hexoside: [M-H]-: 0.471;Isoquercitrin (Quercetin-3-*O*-D-glucoside): [M-H]-: 0.471;Quercimeritrin (Quercetin 7-*O*-glucoside): [M-H]-: 0.471;Hyperoside (Quercetin 3-*O*-galactoside): [M-H]-: 0.471;7-methoxyquercetin-3-*O*-xylopyranose: [M-H]-: 0.471	-2.6	C21H20O12	-	-	-	-	4.59E+02	-	-	-
10	577.1345	1.85	Procyanidin B1: [M-H]-: 0.527;Kaempferol 3-4''-*p*-coumarylrhamnoside: [M-H]-: 0.527	-1.2	C30H26O12	-	-	1.03E+03	9.91E+02	-	-	1.61E+02	-
11	579.1382	1.86	Kaempferol-3-*O*-hexose-pentoside: [M-H]-: 0.333	4.6	C26H28O15	-	-	3.78E+01	6.01E+01	7.50E+02	1.41E+01	2.93E+01	-
12	289.0716	1.87	(+)-catechin: [M-H]-: 0.580;(-)-Epicatechin: [M-H]-: 0.580	-0.6	C15H14O6	-	-	2.32E+02	6.87E+02	-	4.52E+01	5.21E+00	-
13	575.1198	1.90	(Epi)-Catechin dimer: [M-H]-: 0.568	0.5	C30H24O12	-	-	2.40E+01	5.43E+02	-	-	6.80E+01	-
	575.1198	1.90	Dihydrokaempferol	0.5	C15H12O6	-	-	2.40E+01	5.43E+02	-	-	6.80E+01	-

			(Aromadendrol): [2M-H]-: 0.568										
14	593.1522	1.92	Vicenin-2 (Apigenin 6,8-di-*C*-glucoside): [M-H]-: 0.498	1.7	$C_{27}H_{30}O_{15}$	-	-	-	-	4.24E+04	6.18E+00	1.91E+04	1.84E+01
15	463.0874	1.92	Quercetin-3-*O*-hexoside: [M-H]-: 0.514;Isoquercitrin (Quercetin-3-*O*-D-glucoside): [M-H]-: 0.514;Quercimeritrin (Quercetin 7-*O*-glucoside): [M-H]-: 0.514;Hyperoside (Quercetin 3-*O*-galactoside): [M-H]-: 0.514;7-methoxyquercetin-3-*O*-xylopyranose: [M-H]-: 0.514	-1.7	$C_{21}H_{20}O_{12}$	-	-	-	-	3.33E+02	-	-	-
16	577.1346	1.93	Procyanidin B1: [M-H]-: 0.546;Kaempferol 3-4''-*p*-coumarylrhamnoside: [M-H]-: 0.546	-0.9	$C_{30}H_{26}O_{12}$	-	-	1.06E+03	1.43E+03	-	5.56E+01	1.97E+02	-
17	863.1852	1.94	((epi)-catechin-A-(epi)catechin-(epi)catechin): [M-H]-: 0.366	2.7	$C_{45}H_{36}O_{18}$	-	-	1.66E+02	8.76E+03	-	3.36E+01	-	5.38E+00
18	579.1370	1.96	Kaempferol-3-*O*-hexose-pentoside: [M-H]-: 0.460	2.5	$C_{26}H_{28}O_{15}$	-	-	4.80E+01	-	1.34E+03	1.65E+01	1.37E+02	5.16E+00
19	315.0525	1.96	Apigenin: [M+FA]-: 0.446	4.7	$C_{15}H_{10}O_5$	-	-	-	-	-	-	1.95E+02	-
20	563.1421	1.98	Schaftoside (Apigenin 6-*C*-hexoside-8-*C*-pentoside): [M-H]-: 0.455	2.5	$C_{26}H_{28}O_{14}$	-	-	-	-	5.07E+02	-	3.35E+02	-

21	623.1633	1.99	Apigenin 7-*O*-rutinoside: [M+FA]-: 0.453;Vitexin-2``-*O*-rhamnoside: [M+FA]-: 0.453	2.4	C27H30O14	-	-	-	-	3.26E+02	-	-	-
22	593.1527	2.02	Vicenin-2 (Apigenin 6,8-di-*C*-glucoside): [M-H]-: 0.453	2.5	C27H30O15	-	-	-	-	2.22E+04	2.79E+01	1.08E+04	3.10E+01
23	595.1655	2.02	7,4-dimethoxy-3-hydroxyflavone: [2M-H]-: 0.145	7.6	C17H14O5	-	-	-	-	-	-	-	8.92E+02
24	863.1849	2.02	((epi)-catechin-A-(epi)catechin-(epi)catechin): [M-H]-: 0.407	2.3	C45H36O18	-	-	2.19E+02	1.54E+04	-	6.94E+02	2.69E+04	1.66E+01
25	595.1578	2.03	7,4-dimethoxy-3-hydroxyflavone: [2M-H]-: 0.284	-5.4	C17H14O5	-	-	-	-	1.40E+01	-	5.46E+02	-
26	609.1470	2.03	Schaftoside (Apigenin 6-*C*-hexoside-8-*C*-pentoside): [M+FA]-: 0.511	1.5	C26H28O14	-	-	-	-	7.35E+02	6.19E+00	8.72E+02	-
27	563.1420	2.04	Schaftoside (Apigenin 6-*C*-hexoside-8-*C*-pentoside): [M-H]-: 0.462	2.5	C26H28O14	-	-	-	-	4.59E+04	-	3.02E+02	-
28	575.1197	2.05	(Epi)-Catechin dimer: [M-H]-: 0.582	0.4	C30H24O12	-	-	1.12E+02	3.83E+02	-	7.21E+00	3.71E+02	-
	575.1197	2.05	Dihydrokaempferol (Aromadendrol): [2M-H]-: 0.582	0.4	C15H12O6	-	-	1.12E+02	3.83E+02	-	7.21E+00	3.71E+02	-
29	579.1358	2.06	Kaempferol-3-*O*-hexose-pentoside: [M-H]-: 0.589	0.4	C26H28O15	-	-	-	-	7.50E+02	-	1.03E+02	-
30	623.1633	2.07	Apigenin 7-*O*-rutinoside: [M+FA]-: 0.458;Vitexin-2``-*O*-rhamnoside: [M+FA]-: 0.458	2.4	C27H30O14	-	-	-	-	2.38E+02	-	-	-

31	289.0711	2.09	(+)-catechin: [M-H]-: 0.543;(-)-Epicatechin: [M-H]-: 0.543	-2.2	$C_{15}H_{14}O_6$	-	-	7.15E+02	1.75E+03	-	1.26E+02	1.20E+02	-
32	863.1845	2.10	((epi)-catechin-A-(epi)catechin-(epi)catechin): [M-H]-: 0.447	1.9	$C_{45}H_{36}O_{18}$	-	-	1.92E+02	5.91E+03	-	5.49E+02	2.47E+04	1.50E+01
33	593.1525	2.11	Vicenin-2 (Apigenin 6,8-di-*C*-glucoside): [M-H]-: 0.479	2.2	$C_{27}H_{30}O_{15}$	-	-	1.02E+01	-	1.01E+03	-	2.34E+02	-
34	563.1420	2.12	Schaftoside (Apigenin 6-*C*-hexoside-8-*C*-pentoside): [M-H]-: 0.465	2.5	$C_{26}H_{28}O_{14}$	-	-	-	-	1.11E+05	-	-	-
35	609.1470	2.12	Schaftoside (Apigenin 6-*C*-hexoside-8-*C*-pentoside): [M+FA]-: 0.516	1.5	$C_{26}H_{28}O_{14}$	-	-	-	-	8.62E+02	8.33E+00	2.41E+03	4.94E+00
36	575.1198	2.13	(Epi)-Catechin dimer: [M-H]-: 0.576	0.6	$C_{30}H_{24}O_{12}$	-	-	5.26E+01	2.16E+02	-	-	2.67E+02	-
	575.1198	2.13	Dihydrokaempferol (Aromadendrol): [2M-H]-: 0.576	0.6	$C_{15}H_{12}O_6$	-	-	5.26E+01	2.16E+02	-	-	2.67E+02	-
37	447.0930	2.15	Astragalin (Kaempferol-3-*O*-hexoside): [M-H]-: 0.587;Quercitrin (Quercetin 3-*O*-rhamnoside): [M-H]-: 0.577	-0.6	$C_{21}H_{20}O_{11}$	-	-	-	-	2.16E+03	-	-	-
38	593.1512	2.16	Vicenin-2 (Apigenin 6,8-di-*C*-glucoside): [M-H]-: 0.608	0.0	$C_{27}H_{30}O_{15}$	-	-	1.17E+01	-	5.24E+02	5.13E+01	4.42E+02	5.52E+01
39	563.1420	2.18	Schaftoside (Apigenin 6-*C*-hexoside-8-*C*-pentoside): [M-H]-: 0.468	2.5	$C_{26}H_{28}O_{14}$	-	-	-	-	1.16E+05	-	2.42E+04	-

40	577.1342	2.20	Procyanidin B1: [M-H]-: 0.522;Kaempferol 3-4''-*p*-coumarylrhamnoside: [M-H]-: 0.522	-1.6	C30H26O12	-	-	2.04E+02	2.99E+02	-	-	1.02E+01	-
41	609.1470	2.21	Schaftoside (Apigenin 6-*C*-hexoside-8-*C*-pentoside): [M+FA]-: 0.523	1.4	C26H28O14	-	-	-	-	3.62E+02	-	1.28E+03	-
42	483.0699	2.22	Astragalin (Kaempferol-3-*O*-hexoside): [M+Cl]-: 0.610;Quercitrin (Quercetin 3-*O*-rhamnoside): [M+Cl]-: 0.600	-0.1	C21H20O11	-	-	-	-	3.58E+02	-	-	-
43	447.0930	2.23	Astragalin (Kaempferol-3-*O*-hexoside): [M-H]-: 0.591;Quercitrin (Quercetin 3-*O*-rhamnoside): [M-H]-: 0.581	-0.6	C21H20O11	-	-	-	-	6.09E+03	-	-	-
44	895.1988	2.23	Quercitrin (Quercetin 3-*O*-rhamnoside): [2M-H]-: 0.126	5.5	C21H20O11	-	-	-	-	1.56E+02	-	-	-
45	863.1852	2.23	((epi)-catechin-A-(epi)catechin-(epi)catechin): [M-H]-: 0.388	2.6	C45H36O18	-	-	7.09E+01	9.32E+02	-	6.11E+00	2.34E+02	-
46	563.1420	2.25	Schaftoside (Apigenin 6-*C*-hexoside-8-*C*-pentoside): [M-H]-: 0.472	2.5	C26H28O14	-	-	-	-	3.31E+04	-	1.91E+04	-

47	463.0886	2.25	Quercetin-3-*O*-hexoside: [M-H]-: 0.573;Isoquercitrin (Quercetin-3-*O*-D-glucoside): [M-H]-: 0.573;Quercimeritrin (Quercetin 7-*O*-glucoside): [M-H]-: 0.573;Hyperoside (Quercetin 3-*O*-galactoside): [M-H]-: 0.573;7-methoxyquercetin-3-*O*-xylopyranose: [M-H]-: 0.573	0.9	C21H20O12	-	-	-	-	2.31E+02	5.82E+01	-	-
	463.0886	2.25	Kaempferol-3-*O*-pentoside: [M+FA]-: 0.581	0.9	C20H18O10	-	-	-	-	2.31E+02	5.82E+01	-	-
48	477.1041	2.26	Vitexin (Apigenin 8-*C*-glucoside): [M+FA]-: 0.579;Isovitexin (Apigenin 6-*C*-glucoside): [M+FA]-: 0.579;Afzelin (Kaempferol 3-*O*-L-rhamnoside): [M+FA]-: 0.579	0.6	C21H20O10	-	-	-	-	2.81E+02	-	-	-
49	593.1538	2.26	Vicenin-2 (Apigenin 6,8-di-*C*-glucoside): [M-H]-: 0.358	4.3	C27H30O15	-	-	-	-	2.82E+03	-	-	-
50	609.1470	2.27	Schaftoside (Apigenin 6-*C*-hexoside-8-*C*-pentoside): [M+FA]-: 0.527	1.4	C26H28O14	-	-	-	-	1.25E+01	1.49E+01	1.25E+01	1.19E+03
51	447.0930	2.28	Astragalin (Kaempferol-3-*O*-hexoside): [M-H]-: 0.594;Quercitrin (Quercetin 3-*O*-rhamnoside): [M-H]-:	-0.6	C21H20O11	-	-	-	-	5.25E+03	-	4.50E+01	-

			0.584										
52	483.0708	2.29	Quercitrin (Quercetin 3-*O*-rhamnoside): [M+Cl]-: 0.530;Astragalin (Kaempferol-3-*O*-hexoside): [M+Cl]-: 0.520	1.8	$C_{21}H_{20}O_{11}$	-	-	-	-	4.06E+02	-	-	-
53	493.0990	2.30	Quercitrin (Quercetin 3-*O*-rhamnoside): [M+FA]-: 0.594;Astragalin (Kaempferol-3-*O*-hexoside): [M+FA]-: 0.584	0.5	$C_{21}H_{20}O_{11}$	-	-	-	-	3.26E+02	9.73E+00	-	-
54	639.1569	2.31	Vicenin-2 (Apigenin 6,8-di-*C*-glucoside): [M+FA]-: 0.593	0.4	$C_{27}H_{30}O_{15}$	-	-	-	-	-	5.52E+00	6.94E+03	2.99E+01
55	593.1511	2.32	Vicenin-2 (Apigenin 6,8-di-*C*-glucoside): [M-H]-: 0.605	-0.1	$C_{27}H_{30}O_{15}$	-	-	-	-	2.79E+03	-	3.91E+04	-
56	629.1296	2.33	Vicenin-2 (Apigenin 6,8-di-*C*-glucoside): [M+Cl]-: 0.451	2.7	$C_{27}H_{30}O_{15}$	-	-	1.44E+01	-	2.49E+01	-	4.36E+02	-
57	563.1420	2.33	Schaftoside (Apigenin 6-*C*-hexoside-8-*C*-pentoside): [M-H]-: 0.476	2.5	$C_{26}H_{28}O_{14}$	-	-	-	-	2.37E+03	-	2.66E+03	-
58	477.1046	2.33	Vitexin (Apigenin 8-*C*-glucoside): [M+FA]-: 0.536;Isovitexin (Apigenin 6-*C*-glucoside): [M+FA]-: 0.536;Afzelin (Kaempferol 3-*O*-L-rhamnoside): [M+FA]-: 0.536	1.6	$C_{21}H_{20}O_{10}$	-	-	-	-	7.58E+02	-	-	-
59	595.1656	2.35	7,4-dimethoxy-3-hydroxyflavone: [2M-H]-	7.7	$C_{17}H_{14}O_{5}$	-	-	-	-	-	-	-	4.76E+03

			: 0.153										
60	639.1582	2.36	Vicenin-2 (Apigenin 6,8-di-*C*-glucoside): [M+FA]-: 0.464	2.5	C27H30O15	-	-	-	-	2.99E+02	-	7.07E+03	9.85E+00
61	575.1205	2.36	(Epi)-Catechin dimer: [M-H]-: 0.523	1.7	C30H24O12	-	-	6.95E+02	1.93E+02	-	4.04E+01	7.04E+01	-
	575.1205	2.36	Dihydrokaempferol (Aromadendrol): [2M-H]-: 0.523	1.7	C15H12O6	-	-	6.95E+02	1.93E+02	-	4.04E+01	7.04E+01	-
62	609.1470	2.37	Schaftoside (Apigenin 6-*C*-hexoside-8-*C*-pentoside): [M+FA]-: 0.534	1.4	C26H28O14	-	-	-	-	4.17E+01	-	4.68E+02	1.56E+03
63	595.1568	2.38	7,4-dimethoxy-3-hydroxyflavone: [2M-H]-: 0.207	-7.0	C17H14O5	-	-	-	-	9.87E+00	-	2.19E+03	-
64	629.1293	2.38	Vicenin-2 (Apigenin 6,8-di-*C*-glucoside): [M+Cl]-: 0.484	2.2	C27H30O15	-	-	-	-	-	-	4.35E+02	-
65	593.1524	2.38	Vicenin-2 (Apigenin 6,8-di-*C*-glucoside): [M-H]-: 0.504	2.0	C27H30O15	-	-	-	-	-	2.44E+01	3.77E+04	8.01E+01
66	577.1578	2.40	Apigenin 7-*O*-rutinoside: [M-H]-: 0.469;Vitexin-2``-*O*-rhamnoside: [M-H]-: 0.469	2.7	C27H30O14	-	-	-	-	4.53E+03	-	1.46E+02	-
67	609.1470	2.40	Schaftoside (Apigenin 6-*C*-hexoside-8-*C*-pentoside): [M+FA]-: 0.535	1.4	C26H28O14	-	-	-	-	6.41E+01	3.28E+04	8.15E+02	1.70E+03
68	477.1046	2.42	Vitexin (Apigenin 8-*C*-glucoside): [M+FA]-: 0.545;Isovitexin (Apigenin 6-*C*-glucoside): [M+FA]-: 0.545;Afzelin (Kaempferol 3-*O*-L-rhamnoside): [M+FA]-:	1.5	C21H20O10	-	-	-	-	5.91E+02	-	-	-

			0.545										
69	563.1421	2.42	Schaftoside (Apigenin 6-*C*-hexoside-8-*C*-pentoside): [M-H]-: 0.474	2.6	C26H28O14	-	-	-	-	3.53E+02	-	1.39E+03	-
70	623.1631	2.45	Apigenin 7-*O*-rutinoside: [M+FA]-: 0.488;Vitexin-2``-*O*-rhamnoside: [M+FA]-: 0.488	2.2	C27H30O14	-	-	-	-	5.99E+02	5.26E+00	-	9.78E+00
71	577.1569	2.46	Apigenin 7-*O*-rutinoside: [M-H]-: 0.561;Vitexin-2``-*O*-rhamnoside: [M-H]-: 0.561	1.2	C27H30O14	-	-	-	-	5.05E+03	6.23E+00	2.70E+02	8.67E+00
72	595.1632	2.46	7,4-dimethoxy-3-hydroxyflavone: [2M-H]-: 0.394	3.8	C17H14O5	-	-	-	-	2.47E+01	-	-	1.16E+03
73	563.1420	2.47	Schaftoside (Apigenin 6-*C*-hexoside-8-*C*-pentoside): [M-H]-: 0.486	2.4	C26H28O14	-	-	-	-	3.03E+01	-	5.56E+02	-
74	593.1519	2.47	Vicenin-2 (Apigenin 6,8-di-*C*-glucoside): [M-H]-: 0.554	1.2	C27H30O15	-	-	-	1.45E+01	9.76E+01	8.73E+00	2.09E+02	9.28E+03
75	577.1560	2.48	Apigenin 7-*O*-rutinoside: [M-H]-: 0.593;Vitexin-2``-*O*-rhamnoside: [M-H]-: 0.593	-0.4	C27H30O14	-	-	-	-	4.97E+02	-	1.77E+02	1.52E+01
76	609.1470	2.48	Schaftoside (Apigenin 6-*C*-hexoside-8-*C*-pentoside): [M+FA]-: 0.536	1.4	C26H28O14	-	-	2.79E+01	1.98E+04	-	3.34E+04	1.29E+03	2.19E+03
77	623.1632	2.51	Apigenin 7-*O*-rutinoside: [M+FA]-: 0.485;Vitexin-	2.3	C27H30O14	-	-	-	-	4.12E+02	-	9.54E+00	-

			2``-*O*-rhamnoside: [M+FA]-: 0.485										
78	431.0960	2.52	Vitexin (Apigenin 8-*C*-glucoside): [M-H]-: 0.394;Isovitexin (Apigenin 6-*C*-glucoside): [M-H]-: 0.394;Afzelin (Kaempferol 3-*O*-L-rhamnoside): [M-H]-: 0.394	-5.5	C21H20O10	-	-	-	-	1.42E+04	-	4.65E+03	-
79	579.1371	2.53	Kaempferol-3-*O*-hexose-pentoside: [M-H]-: 0.479	2.7	C26H28O15	-	-	-	-	-	-	3.64E+01	8.47E+03
80	595.1637	2.53	7,4-dimethoxy-3-hydroxyflavone: [2M-H]-: 0.350	4.6	C17H14O5	-	-	-	-	2.32E+01	-	-	1.49E+03
81	463.0869	2.54	Quercetin-3-*O*-hexoside: [M-H]-: 0.493;Isoquercitrin (Quercetin-3-*O*-D-glucoside): [M-H]-: 0.493;Quercimeritrin (Quercetin 7-*O*-glucoside): [M-H]-: 0.493;Hyperoside (Quercetin 3-*O*-galactoside): [M-H]-: 0.493;7-methoxyquercetin-3-*O*-xylopyranose: [M-H]-: 0.493	-2.8	C21H20O12	-	-	2.63E+03	3.25E+04	4.93E+02	7.37E+04	4.20E+03	1.55E+03
82	577.1579	2.54	Apigenin 7-*O*-rutinoside: [M-H]-: 0.465;Vitexin-2``-*O*-rhamnoside: [M-H]-: 0.465	2.9	C27H30O14	-	-	-	-	3.62E+02	-	7.79E+01	-
83	477.0662	2.55	Miquelianin (Quercetin 3-*O*-glucuronide): [M-H]-	-2.6	C21H18O13	-	-	-	1.61E+04	-	2.99E+01	1.50E+02	-

			: 0.507										
84	301.0327	2.55	Quercetin: [M-H]-: 0.358	-8.8	$C_{15}H_{10}O_7$	-	-	4.09E+01	1.54E+03	-	5.60E+02	5.73E+01	1.93E+01
85	927.1850	2.56	Quercetin-3-*O*-hexoside: [2M-H]-: 0.500;Isoquercitrin (Quercetin-3-*O*-D-glucoside): [2M-H]-: 0.500;Quercimeritrin (Quercetin 7-*O*-glucoside): [2M-H]-: 0.500;Hyperoside (Quercetin 3-*O*-galactoside): [2M-H]-: 0.500;7-methoxyquercetin-3-*O*-xylopyranose: [2M-H]-: 0.500	1.4	$C_{21}H_{20}O_{12}$	-	-	8.64E+00	1.21E+03	-	7.68E+03	1.72E+01	-
86	461.1095	2.56	Pollenitin 3-*O*-glycoside: [M-H]-: 0.568	1.3	$C_{22}H_{22}O_{11}$	-	-	-	-	6.52E+02	-	-	-
87	955.1418	2.56	Miquelianin (Quercetin 3-*O*-glucuronide): [2M-H]-: 0.600	-0.4	$C_{21}H_{18}O_{13}$	-	-	-	3.84E+02	-	-	-	-
88	451.1034	2.57	Cinchonain Ib: [M-H]-: 0.628	0.0	$C_{24}H_{20}O_9$	-	-	8.55E+02	2.12E+01	-	4.73E+01	-	-
89	593.1514	2.58	Vicenin-2 (Apigenin 6,8-di-*C*-glucoside): [M-H]-: 0.607	0.4	$C_{27}H_{30}O_{15}$	-	-	4.53E+01	-	9.98E+02	1.64E+01	7.26E+03	1.04E+04
90	463.0867	2.59	Quercetin-3-*O*-hexoside: [M-H]-: 0.477;Isoquercitrin (Quercetin-3-*O*-D-glucoside): [M-H]-: 0.477;Quercimeritrin (Quercetin 7-*O*-glucoside): [M-H]-:	-3.2	$C_{21}H_{20}O_{12}$	-	-	1.18E+03	1.12E+04	-	1.46E+04	6.20E+02	3.07E+03

			0.477;Hyperoside (Quercetin 3-*O*-galactoside): [M-H]-: 0.477;7-methoxyquercetin-3-*O*-xylopyranose: [M-H]-: 0.477										
91	447.0930	2.60	Astragalin (Kaempferol-3-*O*-hexoside): [M-H]-: 0.603;Quercitrin (Quercetin 3-*O*-rhamnoside): [M-H]-: 0.593	-0.7	C21H20O11	-	-	-	7.42E+00	6.39E+02	5.82E+01	-	-
92	575.1193	2.61	(Epi)-Catechin dimer: [M-H]-: 0.612	-0.3	C30H24O12	-	-	4.46E+02	1.99E+02	-	4.36E+02	1.14E+03	-
	575.1193	2.61	Dihydrokaempferol (Aromadendrol): [2M-H]-: 0.612	-0.3	C15H12O6	-	-	4.46E+02	1.99E+02	-	4.36E+02	1.14E+03	-
93	461.1081	2.62	Pollenitin 3-*O*-glycoside: [M-H]-: 0.548	-1.8	C22H22O11	-	-	-	-	6.17E+02	-	-	-
94	907.1965	2.62	9,10-Dihydro-10-(4-hydroxyphenyl)-pyrano[2,3-h]epicatechin-8-one: [2M+Cl]-: 0.183	-5.0	C24H20O8	-	-	-	-	2.44E+02	-	-	-
95	579.1376	2.63	Kaempferol-3-*O*-hexose-pentoside: [M-H]-: 0.430	3.6	C26H28O15	-	-	-	-	-	7.75E+01	2.37E+01	1.61E+04
96	579.1376	2.65	Kaempferol-3-*O*-hexose-pentoside: [M-H]-: 0.431	3.6	C26H28O15	-	-	-	-	-	8.68E+01	2.37E+01	8.56E+03
97	593.1517	2.65	Vicenin-2 (Apigenin 6,8-di-*C*-glucoside): [M-H]-: 0.585	0.9	C27H30O15	-	-	7.53E+01	3.65E+02	2.61E+02	7.06E+03	2.02E+04	1.42E+04
98	595.1578	2.65	7,4-dimethoxy-3-hydroxyflavone: [2M-H]-	-5.4	C17H14O5	-	-	-	2.16E+01	-	1.90E+02	8.80E+02	4.88E+02

			: 0.318										
99	461.1082	2.66	Pollenitin 3-*O*-glycoside: [M-H]-: 0.564	-1.5	C22H22O11	-	-	-	-	8.58E+02	-	-	-
100	609.1481	2.70	Schaftoside (Apigenin 6-*C*-hexoside-8-*C*-pentoside): [M+FA]-: 0.435	3.3	C26H28O14	-	-	-	5.49E+00	-	1.38E+02	-	2.53E+02
101	433.0762	2.71	Quercetin 3-*O*-pentoside: [M-H]-: 0.518;Reynoutrin (Quercetin 3-*O*-beta-D-xylopyranoside): [M-H]-: 0.498	-3.2	C20H18O11	-	-	1.64E+03	3.46E+03	4.48E+02	2.63E+04	-	6.89E+03
102	595.1567	2.72	7,4-dimethoxy-3-hydroxyflavone: [2M-H]-: 0.214	-7.2	C17H14O5	-	-	-	1.94E+02	-	4.20E+02	1.51E+02	1.01E+03
103	593.1514	2.72	Vicenin-2 (Apigenin 6,8-di-*C*-glucoside): [M-H]-: 0.614	0.4	C27H30O15	-	-	1.18E+01	4.18E+03	-	8.52E+03	2.92E+03	1.69E+04
104	579.1356	2.75	Kaempferol-3-*O*-hexose-pentoside: [M-H]-: 0.636	0.1	C26H28O15	-	-	-	7.29E+00	-	1.32E+02	-	1.10E+04
105	867.1640	2.75	Reynoutrin (Quercetin 3-*O*-beta-D-xylopyranoside): [2M-H]-: 0.494;Quercetin 3-*O*-pentoside: [2M-H]-: 0.454	1.6	C20H18O11	-	-	-	2.11E+02	-	8.05E+03	1.65E+01	3.21E+01
106	447.0930	2.76	Astragalin (Kaempferol-3-*O*-hexoside): [M-H]-: 0.617;Quercitrin (Quercetin 3-*O*-rhamnoside): [M-H]-: 0.607	-0.6	C21H20O11	-	-	4.08E+03	6.26E+03	-	7.39E+03	3.25E+03	-
107	433.0764	2.76	Quercetin 3-*O*-pentoside: [M-H]-: 0.532;Reynoutrin	-2.9	C20H18O11	-	-	2.91E+03	9.78E+03	-	7.65E+04	2.79E+03	3.57E+03

			(Quercetin 3-*O*-beta-D-xylopyranoside): [M-H]-: 0.512										
108	881.1813	2.76	Kaempferol-3-*O*-pentoside: [2M+FA]-: 0.345	3.6	C20H18O10	-	-	6.71E+00	1.53E+02	-	8.39E+02	9.38E+00	-
109	623.1631	2.77	Apigenin 7-*O*-rutinoside: [M+FA]-: 0.504;Vitexin-2``-*O*-rhamnoside: [M+FA]-: 0.504	2.2	C27H30O14	-	-	-	7.69E+01	-	-	1.97E+04	1.99E+01
110	575.1195	2.79	(Epi)-Catechin dimer: [M-H]-: 0.637	0.1	C30H24O12	-	-	1.76E+02	5.16E+01	-	2.65E+02	4.21E+02	-
	575.1195	2.79	Dihydrokaempferol (Aromadendrol): [2M-H]-: 0.637	0.1	C15H12O6	-	-	1.76E+02	5.16E+01	-	2.65E+02	4.21E+02	-
111	593.1522	2.80	Vicenin-2 (Apigenin 6,8-di-*C*-glucoside): [M-H]-: 0.542	1.7	C27H30O15	-	-	-	-	2.09E+01	4.93E+01	-	1.09E+03
112	579.1363	2.82	Kaempferol-3-*O*-hexose-pentoside: [M-H]-: 0.576	1.3	C26H28O15	-	-	8.51E+00	-	-	3.48E+02	2.80E+01	1.10E+04
113	593.1524	2.83	Vicenin-2 (Apigenin 6,8-di-*C*-glucoside): [M-H]-: 0.520	2.1	C27H30O15	-	-	-	-	9.55E+00	5.58E+01	8.10E+02	1.69E+03
114	269.0443	2.84	Apigenin: [M-H]-: 0.523	-4.5	C15H10O5	-	-	-	-	-	-	3.33E+02	-
115	451.1036	2.85	Cinchonain Ib: [M-H]-: 0.621	0.4	C24H20O9	-	-	3.19E+03	8.31E+01	-	4.65E+01	-	-
116	599.1178	2.85	Schaftoside (Apigenin 6-*C*-hexoside-8-*C*-pentoside): [M+Cl]-: 0.592	0.8	C26H28O14	-	-	-	-	-	-	-	7.34E+02
117	563.1426	2.86	Schaftoside (Apigenin 6-*C*-hexoside-8-*C*-pentoside): [M-H]-: 0.443	3.5	C26H28O14	-	-	-	-	6.64E+00	-	2.10E+02	1.43E+05

118	447.0927	2.87	Astragalin (Kaempferol-3-*O*-hexoside): [M-H]-: 0.594;Quercitrin (Quercetin 3-*O*-rhamnoside): [M-H]-: 0.584	-1.2	C21H20O11	-	5.45E+00	2.05E+04	2.71E+04	5.78E+02	8.61E+04	9.15E+03	1.83E+04
119	895.1971	2.87	Quercitrin (Quercetin 3-*O*-rhamnoside): [2M-H]-: 0.328;Astragalin (Kaempferol-3-*O*-hexoside): [2M-H]-: 0.308	3.6	C21H20O11	-	-	1.73E+03	2.67E+03	-	2.13E+04	8.43E+01	2.99E+02
120	477.1042	2.88	Vitexin (Apigenin 8-*C*-glucoside): [M+FA]-: 0.609;Isovitexin (Apigenin 6-*C*-glucoside): [M+FA]-: 0.609;Afzelin (Kaempferol 3-*O*-L-rhamnoside): [M+FA]-: 0.609	0.6	C21H20O10	-	-	1.45E+01	7.74E+01	1.01E+01	6.18E+01	2.54E+03	5.17E+01
121	431.0965	2.89	Vitexin (Apigenin 8-*C*-glucoside): [M-H]-: 0.461;Isovitexin (Apigenin 6-*C*-glucoside): [M-H]-: 0.461;Afzelin (Kaempferol 3-*O*-L-rhamnoside): [M-H]-: 0.461	-4.4	C21H20O10	-	-	-	-	4.62E+02	-	-	-
122	477.1047	2.92	Vitexin (Apigenin 8-*C*-glucoside): [M+FA]-: 0.561;Isovitexin (Apigenin 6-*C*-glucoside): [M+FA]-: 0.561;Afzelin (Kaempferol 3-*O*-L-rhamnoside): [M+FA]-: 0.561	1.7	C21H20O10	-	-	2.19E+01	1.40E+02	-	6.64E+02	6.00E+02	2.34E+01

123	593.1529	2.95	Vicenin-2 (Apigenin 6,8-di-*C*-glucoside): [M-H]-: 0.484	2.8	C27H30O15	-	-	8.42E+00	-	-	1.29E+02	3.56E+01	5.35E+02
124	417.0813	2.96	Kaempferol-3-*O*-pentoside: [M-H]-: 0.491	-3.5	C20H18O10	-	-	5.97E+02	4.45E+03	4.66E+02	1.18E+04	3.65E+03	1.52E+04
125	907.1978	2.98	9,10-Dihydro-10-(4-hydroxyphenyl)-pyrano[2,3-h]epicatechin-8-one: [2M+Cl]-: 0.331	-3.5	C24H20O8	-	-	-	-	6.40E+02	-	-	-
126	461.1086	2.99	Pollenitin 3-*O*-glycoside: [M-H]-: 0.622	-0.6	C22H22O11	-	-	-	-	2.64E+02	-	-	3.02E+01
127	417.0813	3.00	Kaempferol-3-*O*-pentoside: [M-H]-: 0.496	-3.5	C20H18O10	-	-	1.32E+03	8.41E+02	-	1.62E+04	-	3.05E+04
128	835.1763	3.01	Kaempferol-3-*O*-pentoside: [2M-H]-: 0.310	4.3	C20H18O10	-	-	-	-	-	1.68E+01	-	4.68E+02
129	451.1022	3.04	Cinchonain Ib: [M-H]-: 0.530	-2.8	C24H20O9	-	-	2.52E+02	-	-	-	-	-
130	447.0930	3.06	Astragalin (Kaempferol-3-*O*-hexoside): [M-H]-: 0.628;Quercitrin (Quercetin 3-*O*-rhamnoside): [M-H]-: 0.618	-0.7	C21H20O11	-	-	5.95E+01	5.21E+02	2.42E+01	3.44E+02	4.17E+01	-
131	599.1178	3.06	Schaftoside (Apigenin 6-*C*-hexoside-8-*C*-pentoside): [M+Cl]-: 0.605	0.8	C26H28O14	-	-	-	-	-	-	-	9.21E+02
132	563.1412	3.06	Schaftoside (Apigenin 6-*C*-hexoside-8-*C*-pentoside): [M-H]-: 0.596	1.0	C26H28O14	-	-	-	-	1.10E+01	6.62E+01	5.54E+01	9.93E+04
133	739.1890	3.07	(epi)-afzelechin-(epi)-gallocatechin dimer-7-*O*-glucoside: [M-H]-: 0.549	1.4	C36H36O17	-	-	-	-	4.53E+02	-	-	-

134	417.0820	3.07	Kaempferol-3-*O*-pentoside: [M-H]-: 0.575	-1.6	C20H18O10	-	-	1.93E+03	1.41E+02	-	3.24E+03	4.49E+01	2.00E+02
135	447.0929	3.08	Astragalin (Kaempferol-3-*O*-hexoside): [M-H]-: 0.619;Quercitrin (Quercetin 3-*O*-rhamnoside): [M-H]-: 0.609	-0.9	C21H20O11	-	-	5.04E+01	5.73E+02	1.37E+01	8.36E+02	1.38E+03	2.14E+01
136	577.1570	3.11	Apigenin 7-*O*-rutinoside: [M-H]-: 0.590;Vitexin-2``-*O*-rhamnoside: [M-H]-: 0.590	1.2	C27H30O14	-	-	-	-	-	8.22E+00	2.77E+01	3.02E+03
137	431.0964	3.11	Vitexin (Apigenin 8-*C*-glucoside): [M-H]-: 0.458;Isovitexin (Apigenin 6-*C*-glucoside): [M-H]-: 0.458;Afzelin (Kaempferol 3-*O*-L-rhamnoside): [M-H]-: 0.458	-4.7	C21H20O10	-	-	-	1.41E+01	1.17E+03	-	-	-
138	769.2015	3.13	[(epi)-catechin-(epi)-afzelechin dimer-7-*O*-glucoside: [M+FA]-: 0.358	3.8	C36H36O16	-	-	-	-	5.75E+02	-	1.19E+01	-
139	477.1031	3.18	Vitexin (Apigenin 8-*C*-glucoside): [M+FA]-: 0.592;Isovitexin (Apigenin 6-*C*-glucoside): [M+FA]-: 0.592;Afzelin (Kaempferol 3-*O*-L-rhamnoside): [M+FA]-: 0.592	-1.5	C21H20O10	-	-	1.33E+03	1.48E+02	-	1.10E+03	1.86E+02	1.48E+02
140	285.0383	3.18	Datiscetin: [M-H]-: 0.446;Luteolin: [M-H]-: 0.446;Kaempferol: [M-H]-: 0.446	-7.6	C15H10O6	-	-	1.77E+03	1.54E+02	-	1.21E+03	1.80E+02	1.46E+02

141	863.2053	3.18	Vitexin (Apigenin 8-*C*-glucoside): [2M-H]-: 0.525;Isovitexin (Apigenin 6-*C*-glucoside): [2M-H]-: 0.525;Afzelin (Kaempferol 3-*O*-L-rhamnoside): [2M-H]-: 0.525	1.4	C21H20O10	-	-	5.15E+03	3.67E+01	-	2.21E+03	6.27E+01	2.22E+01
142	467.0744	3.18	Vitexin (Apigenin 8-*C*-glucoside): [M+Cl]-: 0.597;Isovitexin (Apigenin 6-*C*-glucoside): [M+Cl]-: 0.597;Afzelin (Kaempferol 3-*O*-L-rhamnoside): [M+Cl]-: 0.597	-1.4	C21H20O10	-	-	2.90E+02	5.06E+01	-	1.95E+02	6.72E+01	4.37E+01
143	431.0971	3.18	Vitexin (Apigenin 8-*C*-glucoside): [M-H]-: 0.538;Isovitexin (Apigenin 6-*C*-glucoside): [M-H]-: 0.538;Afzelin (Kaempferol 3-*O*-L-rhamnoside): [M-H]-: 0.538	-2.9	C21H20O10	-	-	4.76E+04	5.54E+03	2.79E+01	4.00E+04	6.79E+03	5.00E+03
144	613.1354	3.19	Apigenin 7-*O*-rutinoside: [M+Cl]-: 0.421;Vitexin-2``-*O*-rhamnoside: [M+Cl]-: 0.421	4.0	C27H30O14	-	-	5.88E+02	-	-	-	-	-
145	433.0784	3.22	Reynoutrin (Quercetin 3-*O*-beta-D-xylopyranoside): [M-H]-: 0.579;Quercetin 3-*O*-pentoside: [M-H]-: 0.559	1.9	C20H18O11	-	-	-	-	-	2.86E+02	-	-
146	451.1022	3.22	Cinchonain Ib: [M-H]-: 0.538	-2.8	C24H20O9	-	-	1.71E+03	4.21E+01	-	2.88E+02	-	-

147	431.0982	3.25	Vitexin (Apigenin 8-*C*-glucoside): [M-H]-: 0.652;Isovitexin (Apigenin 6-*C*-glucoside): [M-H]-: 0.652;Afzelin (Kaempferol 3-*O*-L-rhamnoside): [M-H]-: 0.652	-0.4	C21H20O10	-	-	2.15E+02	6.79E+02	-	2.87E+02	1.01E+02	2.73E+01
148	461.1089	3.25	Pollenitin 3-*O*-glycoside: [M-H]-: 0.660	-0.1	C22H22O11	-	-	2.74E+02	6.14E+01	-	7.93E+02	6.42E+02	1.62E+01
149	575.1199	3.25	(Epi)-Catechin dimer: [M-H]-: 0.629	0.6	C30H24O12	-	-	1.42E+02	1.82E+01	-	1.73E+03	8.39E+01	-
	575.1199	3.25	Dihydrokaempferol (Aromadendrol): [2M-H]-: 0.629	0.6	C15H12O6	-	-	1.42E+02	1.82E+01	-	1.73E+03	8.39E+01	-
150	769.2010	3.30	[(epi)-catechin-(epi)-afzelechin dimer-7-*O*-glucoside: [M+FA]-: 0.419	3.2	C36H36O16	-	-	-	-	4.79E+02	-	1.30E+01	5.25E+01
151	433.0758	3.34	Quercetin 3-*O*-pentoside: [M-H]-: 0.508;Reynoutrin (Quercetin 3-*O*-beta-D-xylopyranoside): [M-H]-: 0.488	-4.2	C20H18O11	-	-	-	-	-	2.96E+02	-	-
152	613.1357	3.37	Apigenin 7-*O*-rutinoside: [M+Cl]-: 0.398;Vitexin-2``-*O*-rhamnoside: [M+Cl]-: 0.398	4.5	C27H30O14	-	-	6.95E+02	-	-	-	-	-
153	431.0964	3.38	Vitexin (Apigenin 8-*C*-glucoside): [M-H]-: 0.480;Isovitexin (Apigenin 6-*C*-glucoside): [M-H]-: 0.480;Afzelin (Kaempferol 3-*O*-L-rhamnoside): [M-H]-:	-4.5	C21H20O10	-	-	4.93E+01	2.30E+02	-	6.63E+01	5.19E+00	-

			0.480										
154	769.2010	3.40	[(epi)-catechin-(epi)-afzelechin dimer-7-*O*-glucoside: [M+FA]-: 0.420	3.2	C36H36O16	-	-	-	-	2.88E+02	-	-	-
155	447.0929	3.41	Astragalin (Kaempferol-3-*O*-hexoside): [M-H]-: 0.640;Quercitrin (Quercetin 3-*O*-rhamnoside): [M-H]-: 0.630	-0.8	C21H20O11	-	-	-	-	-	3.77E+02	-	-
156	895.1548	3.45	Mururin A: [2M-H]-: 0.347	3.6	C24H16O9	-	-	1.84E+02	-	-	-	-	-
157	435.1081	3.47	9,10-Dihydro-10-(4-hydroxyphenyl)-pyrano[2,3-h]epicatechin-8-one: [M-H]-: 0.633	-1.0	C24H20O8	-	-	9.46E+01	5.64E+01	-	2.63E+02	-	-
158	447.0716	3.48	Mururin A: [M-H]-: 0.617	-1.3	C24H16O9	-	-	8.39E+03	5.15E+01	-	5.98E+01	-	1.99E+01
159	895.1534	3.49	Mururin A: [2M-H]-: 0.485	2.0	C24H16O9	-	-	1.12E+03	-	-	-	-	-
160	301.0340	3.60	Quercetin: [M-H]-: 0.540	-4.5	C15H10O7	-	-	1.46E+03	6.66E+02	-	5.81E+04	4.79E+02	6.66E+01
161	603.0783	3.60	Quercetin: [2M-H]-: 0.667	0.4	C15H10O7	-	-	7.20E+00	-	-	5.11E+03	-	-
162	447.0718	3.64	Mururin A: [M-H]-: 0.652	-0.7	C24H16O9	-	-	3.02E+03	1.23E+01	-	2.65E+02	2.35E+01	-
163	285.0388	4.12	Datiscetin: [M-H]-: 0.548;Luteolin: [M-H]-: 0.548;Kaempferol: [M-H]-: 0.548	-5.7	C15H10O6	-	-	4.46E+03	2.97E+02	-	2.81E+04	8.48E+02	1.46E+03
164	623.1427	4.27	Procyanidin B1: [M+FA]-: 0.499;Kaempferol 3-4''-*p*-coumarylrhamnoside:	3.4	C30H26O12	-	-	-	-	-	-	-	3.61E+02

			[M+FA]-: 0.499										
165	315.0496	4.28	Apigenin: [M+FA]-: 0.571	-4.6	C15H10O5	-	-	1.84E+01	1.46E+01	-	1.59E+03	3.84E+02	-
166	577.1348	4.35	Procyanidin B1: [M-H]-: 0.689;Kaempferol 3-4''-*p*-coumarylrhamnoside: [M-H]-: 0.689	-0.5	C30H26O12	-	-	3.67E+01	1.04E+02	8.91E+02	3.60E+02	4.86E+01	3.67E+02
167	577.1346	4.43	Procyanidin B1: [M-H]-: 0.664;Kaempferol 3-4''-*p*-coumarylrhamnoside: [M-H]-: 0.664	-1.0	C30H26O12	-	-	3.19E+02	3.33E+02	2.55E+03	7.33E+03	1.25E+03	3.00E+03
168	285.0394	4.43	Datiscetin: [M-H]-: 0.615;Luteolin: [M-H]-: 0.615;Kaempferol: [M-H]-: 0.615	-3.9	C15H10O6	-	-	3.61E+01	1.48E+01	4.13E+01	2.73E+02	4.74E+01	9.85E+01
169	577.1352	4.48	Procyanidin B1: [M-H]-: 0.714;Kaempferol 3-4''-*p*-coumarylrhamnoside: [M-H]-: 0.714	0.1	C30H26O12	-	-	1.38E+03	1.03E+03	-	1.89E+04	5.80E+03	1.34E+04
170	577.1353	4.59	Procyanidin B1: [M-H]-: 0.710;Kaempferol 3-4''-*p*-coumarylrhamnoside: [M-H]-: 0.710	0.3	C30H26O12	-	-	1.87E+02	3.50E+02	5.05E+02	1.43E+03	6.97E+02	1.88E+03
171	769.1786	5.19	Kaempferol 3-(2'',4''-di-(E)-*p*-coumarylrhamnoside): [M+FA]-: 0.643;Kaempferol 3-(3'',4''-di-*p*-coumarylrhamnoside): [M+FA]-: 0.643	1.5	C39H32O14	-	-	-	-	-	-	-	1.10E+03
172	723.1723	5.30	Kaempferol 3-(2'',4''-di-(E)-*p*-coumarylrhamnoside): [M-H]-: 0.725;Kaempferol 3-(3'',4''-di-*p*-	0.5	C39H32O14	-	-	-	8.88E+02	8.60E+02	2.47E+03	3.71E+02	6.85E+03

			coumarylrhamnoside): [M-H]-: 0.725										
173	723.1724	5.38	Kaempferol 3-(2'',4''-di-(E)-*p*-coumarylrhamnoside): [M-H]-: 0.723;Kaempferol 3-(3'',4''-di-*p*-coumarylrhamnoside): [M-H]-: 0.723	0.6	C39H32O14	-	-	8.57E+01	2.04E+03	5.36E+03	3.23E+04	2.34E+03	3.26E+04
174	759.1507	5.38	Kaempferol 3-(2'',4''-di-(E)-*p*-coumarylrhamnoside): [M+Cl]-: 0.563;Kaempferol 3-(3'',4''-di-*p*-coumarylrhamnoside): [M+Cl]-: 0.563	2.7	C39H32O14	-	-	-	2.06E+01	-	3.85E+02	3.09E+01	3.84E+02
175	769.1777	5.38	Kaempferol 3-(2'',4''-di-(E)-*p*-coumarylrhamnoside): [M+FA]-: 0.744;Kaempferol 3-(3'',4''-di-*p*-coumarylrhamnoside): [M+FA]-: 0.744	0.3	C39H32O14	-	-	-	5.81E+01	-	1.96E+03	9.54E+01	3.92E+03
176	769.1793	5.44	Kaempferol 3-(2'',4''-di-(E)-*p*-coumarylrhamnoside): [M+FA]-: 0.579;Kaempferol 3-(3'',4''-di-*p*-coumarylrhamnoside): [M+FA]-: 0.579	2.5	C39H32O14	-	-	-	1.12E+01	-	3.91E+02	1.99E+01	2.30E+02

177	723.1723	5.45	Kaempferol 3-(2'',4''-di-(E)-*p*-coumarylrhamnoside): [M-H]-: 0.731;Kaempferol 3-(3'',4''-di-*p*-coumarylrhamnoside): [M-H]-: 0.731	0.6	C39H32O14	-	-	1.27E+01	6.62E+01	8.82E+01	2.37E+03	7.96E+01	-
178	723.1723	5.55	Kaempferol 3-(2'',4''-di-(E)-*p*-coumarylrhamnoside): [M-H]-: 0.739;Kaempferol 3-(3'',4''-di-*p*-coumarylrhamnoside): [M-H]-: 0.739	0.5	C39H32O14	-	-	-	1.07E+04	8.15E+04	3.48E+04	1.61E+04	5.11E+01
179	769.1778	5.56	Kaempferol 3-(2'',4''-di-(E)-*p*-coumarylrhamnoside): [M+FA]-: 0.740;Kaempferol 3-(3'',4''-di-*p*-coumarylrhamnoside): [M+FA]-: 0.740	0.5	C39H32O14	-	-	-	9.63E+02	2.94E+03	4.83E+03	1.61E+03	4.24E+03
180	759.1488	5.56	Kaempferol 3-(2'',4''-di-(E)-*p*-coumarylrhamnoside): [M+Cl]-: 0.760;Kaempferol 3-(3'',4''-di-*p*-coumarylrhamnoside): [M+Cl]-: 0.760	0.2	C39H32O14	-	-	-	4.01E+01	-	2.67E+02	4.12E+01	1.78E+02
181	723.1727	5.57	Kaempferol 3-(2'',4''-di-(E)-*p*-coumarylrhamnoside): [M-H]-: 0.704;Kaempferol 3-(3'',4''-di-*p*-	1.0	C39H32O14	-	-	6.28E+02	1.16E+04	2.36E+04	6.05E+04	1.71E+04	6.40E+04

			coumarylrhamnoside): [M-H]-: 0.704										
182	723.1723	5.61	Kaempferol 3-(2'',4''-di-(E)-*p*-coumarylrhamnoside): [M-H]-: 0.743;Kaempferol 3-(3'',4''-di-*p*-coumarylrhamnoside): [M-H]-: 0.743	0.5	C39H32O14	-	-	-	7.20E+03	5.77E+03	1.03E+04	5.82E+03	1.43E+04
183	723.1724	5.68	Kaempferol 3-(2'',4''-di-(E)-*p*-coumarylrhamnoside): [M-H]-: 0.734;Kaempferol 3-(3'',4''-di-*p*-coumarylrhamnoside): [M-H]-: 0.734	0.7	C39H32O14	-	-	-	3.11E+03	1.38E+04	1.96E+03	1.68E+03	4.20E+03
184	769.1786	5.73	Kaempferol 3-(2'',4''-di-(E)-*p*-coumarylrhamnoside): [M+FA]-: 0.663;Kaempferol 3-(3'',4''-di-*p*-coumarylrhamnoside): [M+FA]-: 0.663	1.6	C39H32O14	-	-	1.18E+01	4.80E+02	1.07E+03	6.42E+02	4.62E+02	1.10E+03
185	723.1724	5.73	Kaempferol 3-(2'',4''-di-(E)-*p*-coumarylrhamnoside): [M-H]-: 0.742;Kaempferol 3-(3'',4''-di-*p*-coumarylrhamnoside): [M-H]-: 0.742	0.6	C39H32O14	-	-	-	5.24E+03	-	8.95E+03	4.70E+03	1.33E+04

186	723.1738	5.86	Kaempferol 3-(2'',4''-di-(E)-*p*-coumarylrhamnoside): [M-H]-: 0.603;Kaempferol 3-(3'',4''-di-*p*-coumarylrhamnoside): [M-H]-: 0.603	2.6	$C_{39}H_{32}O_{14}$	-	-	1.10E+02	7.90E+02	1.54E+03	3.37E+03	8.93E+02	1.70E+03
187	723.1724	5.99	Kaempferol 3-(2'',4''-di-(E)-*p*-coumarylrhamnoside): [M-H]-: 0.756;Kaempferol 3-(3'',4''-di-*p*-coumarylrhamnoside): [M-H]-: 0.756	0.6	$C_{39}H_{32}O_{14}$	-	-	-	8.48E+01	-	5.19E+03	1.47E+02	1.17E+03
188	723.1744	6.01	Kaempferol 3-(2'',4''-di-(E)-*p*-coumarylrhamnoside): [M-H]-: 0.549;Kaempferol 3-(3'',4''-di-*p*-coumarylrhamnoside): [M-H]-: 0.549	3.5	$C_{39}H_{32}O_{14}$	-	-	9.29E+01	8.24E+02	1.69E+03	1.51E+03	8.84E+02	3.41E+03
189	723.1753	6.14	Kaempferol 3-(2'',4''-di-(E)-*p*-coumarylrhamnoside): [M-H]-: 0.469;Kaempferol 3-(3'',4''-di-*p*-coumarylrhamnoside): [M-H]-: 0.469	4.7	$C_{39}H_{32}O_{14}$	-	-	1.98E+01	1.05E+02	-	7.84E+02	7.62E+01	4.69E+02

(-); peak area equal 0.

Table S2. Level 3 annotation based on MS^1 hits from *Br_flavDB*

N°	*m/z*	RT (min)	*Br_flavDB* annotations (name: adduct: MZmine score)	Error (ppm)	Molecular formula	MS^1 blank replicates Peak area		MS^1 *O.guianensis* Peak area	MS^1 *O.notata* Peak area	MS^1 *O.porosa* Peak area	MS^1 *O.lancifolia* Peak area	MS^1 *O.odorifera* Peak area	MS^1 *O.diospyrifolia* Peak area
1	577.1350	1.62	Procyanidin B-3: [M-H]-: 0.558;Proanthocyanidin: [M-H]-: 0.558;Kaempferol-3-*O*-?-L-(4''-E-*p*-coumaroyl)-rhamnoside: [M-H]-: 0.558;Kaempferol-3-*O*-?-L-(4''-Z-*p*-coumaroyl)-rhamnoside: [M-H]-: 0.558	-0.3	C30H26O12	-	-	6.6E+02	8.3E+02	-	-	-	-
2	323.1337	1.63	5,7-Dihydroxy-6-prenylflavanone: [M-H]-: 0.099	15.0	C20H20O4	-	-	-	-	4.5E+01	-	4.8E+01	7.4E+02
3	575.1205	1.63	(-)-Eriodictyol (2-(3,4-dihydroxyphenyl)-2,3-dihydro-5,7-dihydroxy-4H-chromen-4-one): [2M-H]-: 0.486	1.7	C15H12O6	-	-	-	1.7E+02	-	-	4.4E+02	-
4	577.1351	1.70	Procyanidin B-3: [M-H]-: 0.576;Proanthocyanidin: [M-H]-: 0.576;Kaempferol-3-*O*-?-L-(4''-E-*p*-coumaroyl)-rhamnoside: [M-H]-: 0.576;Kaempferol-3-*O*-?-L-(4''-Z-*p*-coumaroyl)-rhamnoside: [M-H]-: 0.576	-0.1	C30H26O12	-	-	7.2E+02	1.0E+03	-	5.8E+00	5.4E+00	-
5	575.1199	1.71	(-)-Eriodictyol (2-(3,4-dihydroxyphenyl)-2,3-dihydro-5,7-dihydroxy-4H-chromen-4-one): [2M-H]-: 0.551	0.6	C15H12O6	-	-	3.4E+01	2.0E+02	-	-	3.6E+02	-
6	323.1337	1.72	5,7-Dihydroxy-6-prenylflavanone: [M-H]-: 0.108	14.8	C20H20O4	-	-	-	-	2.9E+01	-	2.6E+01	3.0E+02
7	289.0706	1.79	Catechin: [M-H]-: 0.478;(-)-epicatechin: [M-H]-: 0.478	-3.9	C15H14O6	-	-	1.4E+02	3.9E+02	-	1.2E+01	-	-
8	595.1682	1.79	Vitexin 2''-*O*-glucopyranoside: [M-H]-: 0.460	2.2	C27H32O15	-	-	-	-	5.7E+01	1.4E+02	1.2E+02	8.1E+02
9	575.1200	1.81	(-)-Eriodictyol (2-(3,4-dihydroxyphenyl)-2,3-dihydro-5,7-dihydroxy-4H-chromen-4-one): [2M-H]-: 0.539	0.9	C15H12O6	-	-	3.5E+01	4.1E+02	-	-	4.5E+01	-

10	577.1351	1.82	Procyanidin B-3: [M-H]-: 0.586;Proanthocyanidin: [M-H]-: 0.586;Kaempferol-3-*O*-?-L-(4''-E-*p*-coumaroyl)-rhamnoside: [M-H]-: 0.586;Kaempferol-3-*O*-?-L-(4''-Z-*p*-coumaroyl)-rhamnoside: [M-H]-: 0.586	0.0	$C_{30}H_{26}O_{12}$	-	-	4.0E+02	2.5E+02	-	5.9E+01	2.4E+02	-
11	609.1470	1.82	Rutin (Quercetin-3-*O*-rutinoside): [M-H]-: 0.499;Quercetin-3-*O*-L-rhamnopyranosyl-(1-6)-D-galactopyranoside: [M-H]-: 0.499	1.5	$C_{27}H_{30}O_{16}$	-	-	-	-	4.4E+02	-	-	-
12	463.0870	1.84	Hyperin (Quercetin-3-*O*-D-galactoside): [M-H]-: 0.472;Isoquercitrin (Quercetin-3-*O*-D-glucoside): [M-H]-: 0.472;Myricetrin (3-*O*-rhamnosylmyricetin): [M-H]-: 0.472;Quercetin-3-*O*-allopyranoside: [M-H]-: 0.472;Myricetin-3-*O*-L-rhamnopyranoside: [M-H]-: 0.472	-2.6	$C_{21}H_{20}O_{12}$	-	-	-	-	4.6E+02	-	-	-
13	577.1345	1.85	Procyanidin B-3: [M-H]-: 0.521;Proanthocyanidin: [M-H]-: 0.521;Kaempferol-3-*O*-?-L-(4''-E-*p*-coumaroyl)-rhamnoside: [M-H]-: 0.521;Kaempferol-3-*O*-?-L-(4''-Z-*p*-coumaroyl)-rhamnoside: [M-H]-: 0.521	-1.2	$C_{30}H_{26}O_{12}$	-	-	1.0E+03	9.9E+02	-	-	1.6E+02	-
14	579.1382	1.86	Isocarlinoside (Luteolin 6-*C*-arabinoside 8-*C*-glucoside): [M-H]-: 0.325;Carlinoside (Luteolin 6-*C*-D-glucopyranoside-8-*C*-L-arabinopyranoside): [M-H]-: 0.325	4.6	$C_{26}H_{28}O_{15}$	-	-	3.8E+01	6.0E+01	7.5E+02	1.4E+01	2.9E+01	-
15	289.0716	1.87	Catechin: [M-H]-: 0.576;(-)-epicatechin: [M-H]-: 0.576	-0.6	$C_{15}H_{14}O_{6}$	-	-	2.3E+02	6.9E+02	-	4.5E+01	5.2E+00	-
16	575.1198	1.90	(-)-Eriodictyol (2-(3,4-dihydroxyphenyl)-2,3-dihydro-5,7-dihydroxy-4H-chromen-4-one): [2M-H]-: 0.566	0.5	$C_{15}H_{12}O_{6}$	-	-	2.4E+01	5.4E+02	-	-	6.8E+01	-
17	595.1662	1.91	Vitexin 2''-*O*-glucopyranoside: [M-H]-: 0.532	-1.1	$C_{27}H_{32}O_{15}$	-	-	8.5E+00	-	-	6.8E+01	-	1.9E+03

18	593.1522	1.92	Nicotiflorin (Kaempferol-3-*O*-rutinoside): [M-H]-: 0.493;Kaempferol-3-*O*-L-rhamnopyranosyl-(1-6)-D-galactopyranoside: [M-H]-: 0.493	1.7	$C_{27}H_{30}O_{15}$	-	-	-	-	4.2E+04	6.2E+00	1.9E+04	1.8E+01
19	463.0874	1.92	Hyperin (Quercetin-3-*O*-D-galactoside): [M-H]-: 0.515;Isoquercitrin (Quercetin-3-*O*-D-glucoside): [M-H]-: 0.515;Myricetrin (3-*O*-rhamnosylmyricetin): [M-H]-: 0.515;Quercetin-3-*O*-allopyranoside: [M-H]-: 0.515;Myricetin-3-*O*-L-rhamnopyranoside: [M-H]-: 0.515	-1.7	$C_{21}H_{20}O_{12}$	-	-	-	-	3.3E+02	-	-	-
20	577.1346	1.93	Procyanidin B-3: [M-H]-: 0.540;Proanthocyanidin: [M-H]-: 0.540;Kaempferol-3-*O*-?-L-(4''-E-*p*-coumaroyl)-rhamnoside: [M-H]-: 0.540;Kaempferol-3-*O*-?-L-(4''-Z-*p*-coumaroyl)-rhamnoside: [M-H]-: 0.540	-0.9	$C_{30}H_{26}O_{12}$	-	-	1.1E+03	1.4E+03	-	5.6E+01	2.0E+02	-
21	579.1370	1.96	Isocarlinoside (Luteolin 6-*C*-arabinoside 8-*C*-glucoside): [M-H]-: 0.452;Carlinoside (Luteolin 6-*C*-D-glucopyranoside-8-*C*-L-arabinopyranoside): [M-H]-: 0.452	2.5	$C_{26}H_{28}O_{15}$	-	-	4.8E+01	-	1.3E+03	1.6E+01	1.4E+02	5.2E+00
22	315.0525	1.96	Pedalitin: [M-H]-: 0.450;3-*O*-methylquercetin: [M-H]-: 0.450;Isorhamnetin: [M-H]-: 0.450	4.7	$C_{16}H_{12}O_{7}$	-	-	-	-	-	-	2.0E+02	-
23	563.1421	1.98	Schaftoside (Apigenin-6-glucoside-8-arabinoside): [M-H]-: 0.454;Isoschaftoside (Apigenin-6-arabnoside-8-glucoside): [M-H]-: 0.454	2.5	$C_{26}H_{28}O_{14}$	-	-	-	-	5.1E+02	-	3.3E+02	-
24	623.1633	1.99	Nitensoside B (Pedalitin 6-*O*-rhamnopyranosyl(1'''6'')-glucopyranoside): [M-H]-: 0.452	2.4	$C_{28}H_{32}O_{16}$	-	-	-	-	3.3E+02	-	-	-

25	593.1527	2.02	Nicotiflorin (Kaempferol-3-*O*-rutinoside): [M-H]-: 0.448;Kaempferol-3-*O*-L-rhamnopyranosyl-(1-6)-D-galactopyranoside: [M-H]-: 0.448	2.5	$C_{27}H_{30}O_{15}$	-	-	-	-	2.2E+04	2.8E+01	1.1E+04	3.1E+01
26	595.1655	2.02	Vitexin 2''-*O*-glucopyranoside: [M-H]-: 0.464	-2.3	$C_{27}H_{32}O_{15}$	-	-	-	-	-	-	-	8.9E+02
27	595.1578	2.03	Tithonine: [2M-H]-: 0.276;Afrormosin/Afromosin (7-Hydroxy-4',6-dimethoxyisoflavone) : [2M-H]-: 0.276;8-*O*-methylretusin: [2M-H]-: 0.276	-5.4	$C_{17}H_{14}O_5$	-	-	-	-	1.4E+01	-	5.5E+02	-
28	609.1470	2.03	Rutin (Quercetin-3-*O*-rutinoside): [M-H]-: 0.511;Quercetin-3-*O*-L-rhamnopyranosyl-(1-6)-D-galactopyranoside: [M-H]-: 0.511	1.5	$C_{27}H_{30}O_{16}$	-	-	-	-	7.4E+02	6.2E+00	8.7E+02	-
29	563.1420	2.04	Schaftoside (Apigenin-6-glucoside-8-arabinoside): [M-H]-: 0.461;Isoschaftoside (Apigenin-6-arabnoside-8-glucoside): [M-H]-: 0.461	2.5	$C_{26}H_{28}O_{14}$	-	-	-	-	4.6E+04	-	3.0E+02	-
30	575.1197	2.05	(-)-Eriodictyol (2-(3,4-dihydroxyphenyl)-2,3-dihydro-5,7-dihydroxy-4H-chromen-4-one): [2M-H]-: 0.580	0.4	$C_{15}H_{12}O_6$	-	-	1.1E+02	3.8E+02	-	7.2E+00	3.7E+02	-
31	393.1389	2.06	7-*O*-dimethylallyl-3'-acetate-4'-methoxyisoflavone: [M-H]-: 0.153	11.4	$C_{23}H_{22}O_6$	-	-	2.9E+01	2.8E+01	-	-	-	2.9E+02
32	579.1358	2.06	Isocarlinoside (Luteolin 6-*C*-arabinoside 8-*C*-glucoside): [M-H]-: 0.581;Carlinoside (Luteolin 6-*C*-D-glucopyranoside-8-*C*-L-arabinopyranoside): [M-H]-: 0.581	0.4	$C_{26}H_{28}O_{15}$	-	-	-	-	7.5E+02	-	1.0E+02	-
33	623.1633	2.07	Nitensoside B (Pedalitin 6-*O*-rhamnopyranosyl(1'''6'')-glucopyranoside): [M-H]-: 0.458	2.4	$C_{28}H_{32}O_{16}$	-	-	-	-	2.4E+02	-	-	-
34	289.0711	2.09	Catechin: [M-H]-: 0.539;(-)-epicatechin: [M-H]-: 0.539	-2.2	$C_{15}H_{14}O_6$	-	-	7.1E+02	1.8E+03	-	1.3E+02	1.2E+02	-
35	323.1239	2.10	5,7-Dihydroxy-6-prenylflavanone: [M-H]-:	-15.3	$C_{20}H_{20}O_4$	-	-	5.4E+02	1.3E+01	2.5E+01	8.6E+01	6.5E+01	1.9E+01

			0.109										
36	593.1525	2.11	Nicotiflorin (Kaempferol-3-*O*-rutinoside): [M-H]-: 0.474;Kaempferol-3-*O*-L-rhamnopyranosyl-(1-6)-D-galactopyranoside: [M-H]-: 0.474	2.2	C27H30O15	-	-	1.0E+01	-	1.0E+03	-	2.3E+02	-
37	563.1420	2.12	Schaftoside (Apigenin-6-glucoside-8-arabinoside): [M-H]-: 0.464;Isoschaftoside (Apigenin-6-arabnoside-8-glucoside): [M-H]-: 0.464	2.5	C26H28O14	-	-	-	-	1.1E+05	-	-	-
38	609.1470	2.12	Rutin (Quercetin-3-*O*-rutinoside): [M-H]-: 0.517;Quercetin-3-*O*-L-rhamnopyranosyl-(1-6)-D-galactopyranoside: [M-H]-: 0.517	1.5	C27H30O16	-	-	-	-	8.6E+02	8.3E+00	2.4E+03	4.9E+00
39	755.2051	2.12	Kaempferol-3-*O*-L-rhamnopyranosyl-(1-6)-L-rhamnopyranosyl-(1-4)-D-glucopyranoside: [M-H]-: 0.494	1.5	C33H40O20	-	-	-	-	7.4E+00	8.5E+00	2.1E+01	1.2E+03
40	575.1198	2.13	(-)-Eriodictyol (2-(3,4-dihydroxyphenyl)-2,3-dihydro-5,7-dihydroxy-4H-chromen-4-one): [2M-H]-: 0.574	0.6	C15H12O6	-	-	5.3E+01	2.2E+02	-	-	2.7E+02	-
41	447.0930	2.15	Orientin (Luteolin 8-*C*-glucoside): [M-H]-: 0.581;Quercitrin (3-*O*-rhamnosylquercetin): [M-H]-: 0.581;Trifolin (Kaempferol-3-*O*-D-galactopyranoside): [M-H]-: 0.581;Kaempferol-3-*O*-D-glucopyranoside: [M-H]-: 0.581;Isoorientin (Luteolin-6-glucoside): [M-H]-: 0.581;Quercetin-3-*O*-L-rhamnopyranoside: [M-H]-: 0.581	-0.6	C21H20O11	-	-	-	-	2.2E+03	-	-	-
42	593.1512	2.16	Nicotiflorin (Kaempferol-3-*O*-rutinoside): [M-H]-: 0.603;Kaempferol-3-*O*-L-rhamnopyranosyl-(1-6)-D-galactopyranoside: [M-H]-: 0.603	0.0	C27H30O15	-	-	1.2E+01	-	5.2E+02	5.1E+01	4.4E+02	5.5E+01

43	563.1420	2.18	Schaftoside (Apigenin-6-glucoside-8-arabinoside): [M-H]-: 0.467;Isoschaftoside (Apigenin-6-arabnoside-8-glucoside): [M-H]-: 0.467	2.5	C26H28O14	-	-	-	-	1.2E+05	-	2.4E+04	-
44	577.1342	2.20	Procyanidin B-3: [M-H]-: 0.516;Proanthocyanidin: [M-H]-: 0.516;Kaempferol-3-*O*-?-L-(4''-E-*p*-coumaroyl)-rhamnoside: [M-H]-: 0.516;Kaempferol-3-*O*-?-L-(4''-Z-*p*-coumaroyl)-rhamnoside: [M-H]-: 0.516	-1.6	C30H26O12	-	-	2.0E+02	3.0E+02	-	-	1.0E+01	-
45	755.2069	2.20	Kaempferol-3-*O*-L-rhamnopyranosyl-(1-6)-L-rhamnopyranosyl-(1-4)-D-glucopyranoside: [M-H]-: 0.321	3.8	C33H40O20	-	-	-	-	-	1.1E+01	5.3E+01	6.7E+02
46	373.1297	2.20	3',4',5,5',7-pentamethoxyflavanone: [M-H]-: 0.568;2',3',4',6,7-Pentametoxyisoflavanone: [M-H]-: 0.568	1.2	C20H22O7	-	-	-	-	-	-	-	8.6E+02
47	361.1292	2.20	1-(2'-Hydroxy-4'-methoxy-5'-methylphenyl)-3-(2''-hydroxy-4'',5''-methylenedioxyphenyl)-propane: [M+FA]-: 0.600;5-methoxy-2-(3-(5-methoxybenzo[d][1,3]dioxol-6-yl)propyl)phenol: [M+FA]-: 0.600;4-(3-(5-hydroxybenzo[d][1,3]dioxol-6-yl)propyl)-2,6-dimethylbenzene-1,3-diol: [M+FA]-: 0.600;6-(3-(2-hydroxy-4-methoxy-5-methylphenyl)propyl)benzo[d][1,3]dioxol-5-ol: [M+FA]-: 0.600	-0.3	C18H20O5	-	-	-	-	-	-	-	5.5E+02
48	609.1470	2.21	Rutin (Quercetin-3-*O*-rutinoside): [M-H]-: 0.523;Quercetin-3-*O*-L-rhamnopyranosyl-(1-6)-D-galactopyranoside: [M-H]-: 0.523	1.4	C27H30O16	-	-	-	-	3.6E+02	-	1.3E+03	-

49	483.0699	2.22	Orientin (Luteolin 8-*C*-glucoside): [M+Cl]-: 0.604;Quercitrin (3-*O*-rhamnosylquercetin): [M+Cl]-: 0.604;Trifolin (Kaempferol-3-*O*-D-galactopyranoside): [M+Cl]-: 0.604;Kaempferol-3-*O*-D-glucopyranoside: [M+Cl]-: 0.604;Isoorientin (Luteolin-6-glucoside): [M+Cl]-: 0.604;Quercetin-3-*O*-L-rhamnopyranoside: [M+Cl]-: 0.604;3',4',5',5,6,7,-Hexamethoxyflavanone: [M+Br]-: 0.223	-0.1	C21H20O11	-	-	-	-	3.6E+02	-	-	-
50	447.0930	2.23	Orientin (Luteolin 8-*C*-glucoside): [M-H]-: 0.585;Quercitrin (3-*O*-rhamnosylquercetin): [M-H]-: 0.585;Trifolin (Kaempferol-3-*O*-D-galactopyranoside): [M-H]-: 0.585;Kaempferol-3-*O*-D-glucopyranoside: [M-H]-: 0.585;Isoorientin (Luteolin-6-glucoside): [M-H]-: 0.585;Quercetin-3-*O*-L-rhamnopyranoside: [M-H]-: 0.585	-0.6	C21H20O11	-	-	-	-	6.1E+03	-	-	-
51	895.1988	2.23	Orientin (Luteolin 8-*C*-glucoside): [2M-H]-: 0.118;Quercitrin (3-*O*-rhamnosylquercetin): [2M-H]-: 0.118;Trifolin (Kaempferol-3-*O*-D-galactopyranoside): [2M-H]-: 0.118;Kaempferol-3-*O*-D-glucopyranoside: [2M-H]-: 0.118;Isoorientin (Luteolin-6-glucoside): [2M-H]-: 0.118;Quercetin-3-*O*-L-rhamnopyranoside: [2M-H]-: 0.118	5.5	C21H20O11	-	-	-	-	1.6E+02	-	-	-
52	563.1420	2.25	Schaftoside (Apigenin-6-glucoside-8-arabinoside): [M-H]-: 0.471;Isoschaftoside (Apigenin-6-arabnoside-8-glucoside): [M-H]-: 0.471	2.5	C26H28O14	-	-	-	-	3.3E+04	-	1.9E+04	-

53	463.0886	2.25	Hyperin (Quercetin-3-*O*-D-galactoside): [M-H]-: 0.572;Isoquercitrin (Quercetin-3-*O*-D-glucoside): [M-H]-: 0.572;Myricetrin (3-*O*-rhamnosylmyricetin): [M-H]-: 0.572;Quercetin-3-*O*-allopyranoside: [M-H]-: 0.572;Myricetin-3-*O*-L-rhamnopyranoside: [M-H]-: 0.572	0.9	C21H20O12	-	-	-	-	2.3E+02	5.8E+01	-	-
54	477.1041	2.26	Pedalin (pedalitin 6-*O*-glucopyranoside): [M-H]-: 0.587	0.6	C22H22O12	-	-	-	-	2.8E+02	-	-	-
55	595.1662	2.26	Vitexin 2''-*O*-glucopyranoside: [M-H]-: 0.543	-1.2	C27H32O15	-	-	-	-	-	-	-	4.3E+03
56	593.1538	2.26	Nicotiflorin (Kaempferol-3-*O*-rutinoside): [M-H]-: 0.353;Kaempferol-3-*O*-L-rhamnopyranosyl-(1-6)-D-galactopyranoside: [M-H]-: 0.353	4.3	C27H30O15	-	-	-	-	2.8E+03	-	-	-
57	609.1470	2.27	Rutin (Quercetin-3-*O*-rutinoside): [M-H]-: 0.527;Quercetin-3-*O*-L-rhamnopyranosyl-(1-6)-D-galactopyranoside: [M-H]-: 0.527	1.4	C27H30O16	-	-	-	-	1.3E+01	1.5E+01	1.2E+01	1.2E+03
58	361.1289	2.28	1-(2'-Hydroxy-4'-methoxy-5'-methylphenyl)-3-(2''-hydroxy-4'',5''-methylenedioxyphenyl)-propane: [M+FA]-: 0.577;5-methoxy-2-(3-(5-methoxybenzo[d][1,3]dioxol-6-yl)propyl)phenol: [M+FA]-: 0.577;4-(3-(5-hydroxybenzo[d][1,3]dioxol-6-yl)propyl)-2,6-dimethylbenzene-1,3-diol: [M+FA]-: 0.577;6-(3-(2-hydroxy-4-methoxy-5-methylphenyl)propyl)benzo[d][1,3]dioxol-5-ol: [M+FA]-: 0.577	-1.0	C18H20O5	-	-	-	-	-	-	6.1E+00	6.5E+02

59	447.0930	2.28	Orientin (Luteolin 8-*C*-glucoside): [M-H]-: 0.588;Quercitrin (3-*O*-rhamnosylquercetin): [M-H]-: 0.588;Trifolin (Kaempferol-3-*O*-D-galactopyranoside): [M-H]-: 0.588;Kaempferol-3-*O*-D-glucopyranoside: [M-H]-: 0.588;Isoorientin (Luteolin-6-glucoside): [M-H]-: 0.588;Quercetin-3-*O*-L-rhamnopyranoside: [M-H]-: 0.588	-0.6	$C_{21}H_{20}O_{11}$	-	-	-	-	5.2E+03	-	4.5E+01	-
60	373.1292	2.28	3',4',5,5',7-pentamethoxyflavanone: [M-H]-: 0.605;2',3',4',6,7-Pentametoxyisoflavanone: [M-H]-: 0.605	-0.2	$C_{20}H_{22}O_7$	-	-	1.9E+01	-	-	-	1.1E+01	1.1E+03
61	313.1077	2.29	(2R)-5-Hydroxy-7,4'-Dimethoxy-8-Methylflavanone: [M-H]-: 0.570;(2R)-5-Hydroxy-7,4'-dimethoxy-6-methylflavanone: [M-H]-: 0.570;7,2'-Dihydroxy-6,8-dimethyl-4',5'-methylenedioxyflavan: [M-H]-: 0.570;7,2'-Dihydroxy-5,8-dimethyl-4',5'-methylenedioxyflavan: [M-H]-: 0.570;(2S)-5,7-Dihydroxy-4'-methoxy-6,8-dimethylflavanone: [M-H]-: 0.570	-1.4	$C_{18}H_{18}O_5$	-	-	-	-	-	-	-	3.3E+02
62	483.0708	2.29	Orientin (Luteolin 8-*C*-glucoside): [M+Cl]-: 0.526;Quercitrin (3-*O*-rhamnosylquercetin): [M+Cl]-: 0.526;Trifolin (Kaempferol-3-*O*-D-galactopyranoside): [M+Cl]-: 0.526;Kaempferol-3-*O*-D-glucopyranoside: [M+Cl]-: 0.526;Isoorientin (Luteolin-6-glucoside): [M+Cl]-: 0.526;Quercetin-3-*O*-L-rhamnopyranoside: [M+Cl]-: 0.526;3',4',5',5,6,7,-Hexamethoxyflavanone: [M+Br]-: 0.132	1.8	$C_{21}H_{20}O_{11}$	-	-	-	-	4.1E+02	-	-	-

63	493.0990	2.30	Orientin (Luteolin 8-*C*-glucoside): [M+FA]-: 0.590;Quercitrin (3-*O*-rhamnosylquercetin): [M+FA]-: 0.590;Trifolin (Kaempferol-3-*O*-D-galactopyranoside): [M+FA]-: 0.590;Kaempferol-3-*O*-D-glucopyranoside: [M+FA]-: 0.590;Isoorientin (Luteolin-6-glucoside): [M+FA]-: 0.590;Quercetin-3-*O*-L-rhamnopyranoside: [M+FA]-: 0.590	0.5	C21H20O11	-	-	-	-	3.3E+02	9.7E+00	-	-
64	639.1569	2.31	Nicotiflorin (Kaempferol-3-*O*-rutinoside): [M+FA]-: 0.588;Kaempferol-3-*O*-L-rhamnopyranosyl-(1-6)-D-galactopyranoside: [M+FA]-: 0.588	0.4	C27H30O15	-	-	-	-	-	5.5E+00	6.9E+03	3.0E+01
65	435.2225	2.31	11-decyl-11,12-dihydro-5,10-dioxatetraphen-12-one: [M+FA]-: 0.134	11.0	C26H30O3	-	-	4.4E+01	-	4.4E+02	4.4E+01	5.8E+02	8.5E+00
66	593.1511	2.32	Nicotiflorin (Kaempferol-3-*O*-rutinoside): [M-H]-: 0.610;Kaempferol-3-*O*-L-rhamnopyranosyl-(1-6)-D-galactopyranoside: [M-H]-: 0.610	-0.1	C27H30O15	-	-	-	-	2.8E+03	-	3.9E+04	-
67	629.1296	2.33	Nicotiflorin (Kaempferol-3-*O*-rutinoside): [M+Cl]-: 0.446;Kaempferol-3-*O*-L-rhamnopyranosyl-(1-6)-D-galactopyranoside: [M+Cl]-: 0.446	2.7	C27H30O15	-	-	1.4E+01	-	2.5E+01	-	4.4E+02	-
68	563.1420	2.33	Schaftoside (Apigenin-6-glucoside-8-arabinoside): [M-H]-: 0.475;Isoschaftoside (Apigenin-6-arabnoside-8-glucoside): [M-H]-: 0.475	2.5	C26H28O14	-	-	-	-	2.4E+03	-	2.7E+03	-
69	477.1046	2.33	Pedalin (pedalitin 6-*O*-glucopyranoside): [M-H]-: 0.543	1.6	C22H22O12	-	-	-	-	7.6E+02	-	-	-
70	595.1656	2.35	Vitexin 2''-*O*-glucopyranoside: [M-H]-: 0.489	-2.1	C27H32O15	-	-	-	-	-	-	-	4.8E+03
71	639.1582	2.36	Nicotiflorin (Kaempferol-3-*O*-rutinoside): [M+FA]-: 0.459;Kaempferol-3-*O*-L-rhamnopyranosyl-(1-6)-D-galactopyranoside: [M+FA]-: 0.459	2.5	C27H30O15	-	-	-	-	3.0E+02	-	7.1E+03	9.8E+00

72	575.1205	2.36	(-)-Eriodictyol (2-(3,4-dihydroxyphenyl)-2,3-dihydro-5,7-dihydroxy-4H-chromen-4-one): [2M-H]-: 0.521	1.7	$C_{15}H_{12}O_6$	-	-	7.0E+02	1.9E+02	-	4.0E+01	7.0E+01	-
73	609.1470	2.37	Rutin (Quercetin-3-*O*-rutinoside): [M-H]-: 0.534;Quercetin-3-*O*-L-rhamnopyranosyl-(1-6)-D-galactopyranoside: [M-H]-: 0.534	1.4	$C_{27}H_{30}O_{16}$	-	-	-	-	4.2E+01	-	4.7E+02	1.6E+03
74	595.1568	2.38	Tithonine: [2M-H]-: 0.199;Afrormosin/Afromosin (7-Hydroxy-4',6-dimethoxyisoflavone) : [2M-H]-: 0.199;8-*O*-methylretusin: [2M-H]-: 0.199	-7.0	$C_{17}H_{14}O_5$	-	-	-	-	9.9E+00	-	2.2E+03	-
75	629.1293	2.38	Nicotiflorin (Kaempferol-3-*O*-rutinoside): [M+Cl]-: 0.479;Kaempferol-3-*O*-L-rhamnopyranosyl-(1-6)-D-galactopyranoside: [M+Cl]-: 0.479	2.2	$C_{27}H_{30}O_{15}$	-	-	-	-	-	-	4.3E+02	-
76	593.1524	2.38	Nicotiflorin (Kaempferol-3-*O*-rutinoside): [M-H]-: 0.499;Kaempferol-3-*O*-L-rhamnopyranosyl-(1-6)-D-galactopyranoside: [M-H]-: 0.499	2.0	$C_{27}H_{30}O_{15}$	-	-	-	-	-	2.4E+01	3.8E+04	8.0E+01
77	327.0865	2.40	3,7,4-*O*-trimethylkaempferol: [M-H]-: 0.530;5-Hydroxy-7,2',4'-trimethoxyisoflavone: [M-H]-: 0.530;4-*O*-Methyl-4',5'-O,O-methylidene-mopanol: [M-H]-: 0.530;7-hydroxy-8,3',4'-trimethoxyisoflavone: [M-H]-: 0.530	-2.7	$C_{18}H_{16}O_6$	-	-	3.4E+02	6.1E+00	1.8E+01	4.9E+01	1.8E+01	-
	327.0865	2.40	7,4'-Dimethoxyflavone: [M+FA]-	-2.7	$C_{17}H_{14}O_4$	-	-	3.4E+02	6.1E+00	1.8E+01	4.9E+01	1.8E+01	-
78	577.1578	2.40	Kaempferitrin (Kaempferol 3,7-*O*-di-rhamnoside): [M-H]-: 0.466	2.7	$C_{27}H_{30}O_{14}$	-	-	-	-	4.5E+03	-	1.5E+02	-
79	609.1470	2.40	Rutin (Quercetin-3-*O*-rutinoside): [M-H]-: 0.535;Quercetin-3-*O*-L-rhamnopyranosyl-(1-6)-D-galactopyranoside: [M-H]-: 0.535	1.4	$C_{27}H_{30}O_{16}$	-	-	-	-	6.4E+01	3.3E+04	8.2E+02	1.7E+03
80	477.1046	2.42	Pedalin (pedalitin 6-*O*-glucopyranoside): [M-H]-: 0.552	1.5	$C_{22}H_{22}O_{12}$	-	-	-	-	5.9E+02	-	-	-

81	563.1421	2.42	Schaftoside (Apigenin-6-glucoside-8-arabinoside): [M-H]-: 0.473;Isoschaftoside (Apigenin-6-arabnoside-8-glucoside): [M-H]-: 0.473	2.6	$C_{26}H_{28}O_{14}$	-	-	-	-	3.5E+02	-	1.4E+03	-
82	623.1631	2.45	Nitensoside B (Pedalitin 6-*O*-rhamnopyranosyl(1'''6'')-glucopyranoside): [M-H]-: 0.487	2.2	$C_{28}H_{32}O_{16}$	-	-	-	-	6.0E+02	5.3E+00	-	9.8E+00
83	577.1569	2.46	Kaempferitrin (Kaempferol 3,7-*O*-di-rhamnoside): [M-H]-: 0.558	1.2	$C_{27}H_{30}O_{14}$	-	-	-	-	5.0E+03	6.2E+00	2.7E+02	8.7E+00
84	595.1632	2.46	Vitexin 2''-*O*-glucopyranoside: [M-H]-: 0.260	-6.1	$C_{27}H_{32}O_{15}$	-	-	-	-	2.5E+01	-	-	1.2E+03
	595.1632	2.46	Tithonine: [2M-H]-: 0.402;Afrormosin/Afromosin (7-Hydroxy-4',6-dimethoxyisoflavone) : [2M-H]-: 0.402;8-*O*-methylretusin: [2M-H]-: 0.402	3.8	$C_{17}H_{14}O_5$	-	-	-	-	2.5E+01	-	-	1.2E+03
85	563.1420	2.47	Schaftoside (Apigenin-6-glucoside-8-arabinoside): [M-H]-: 0.485;Isoschaftoside (Apigenin-6-arabnoside-8-glucoside): [M-H]-: 0.485	2.4	$C_{26}H_{28}O_{14}$	-	-	-	-	3.0E+01	-	5.6E+02	-
86	593.1519	2.47	Nicotiflorin (Kaempferol-3-*O*-rutinoside): [M-H]-: 0.549;Kaempferol-3-*O*-L-rhamnopyranosyl-(1-6)-D-galactopyranoside: [M-H]-: 0.549	1.2	$C_{27}H_{30}O_{15}$	-	-	-	1.4E+01	9.8E+01	8.7E+00	2.1E+02	9.3E+03
87	577.1560	2.48	Kaempferitrin (Kaempferol 3,7-*O*-di-rhamnoside): [M-H]-: 0.596	-0.4	$C_{27}H_{30}O_{14}$	-	-	-	-	5.0E+02	-	1.8E+02	1.5E+01
88	609.1470	2.48	Rutin (Quercetin-3-*O*-rutinoside): [M-H]-: 0.537;Quercetin-3-*O*-L-rhamnopyranosyl-(1-6)-D-galactopyranoside: [M-H]-: 0.537	1.4	$C_{27}H_{30}O_{16}$	-	-	2.8E+01	2.0E+04	-	3.3E+04	1.3E+03	2.2E+03
89	311.0562	2.50	7-hydroxy-6-methoxy-3',4'-methylenedioxyisoflavone: [M-H]-: 0.613	0.4	$C_{17}H_{12}O_6$	-	-	-	-	7.2E+02	-	8.3E+01	-

90	623.1632	2.51	Nitensoside B (Pedalitin 6-*O*-rhamnopyranosyl(1'''6'')-glucopyranoside): [M-H]-: 0.484	2.3	$C_{28}H_{32}O_{16}$	-	-	-	-	4.1E+02	-	9.5E+00	-
91	431.0960	2.52	Afzelin (Kaempferol 3-*O*-rhamnoside): [M-H]-: 0.389	-5.5	$C_{21}H_{20}O_{10}$	-	-	-	-	1.4E+04	-	4.6E+03	-
	431.0960	2.52	3',4'-methylenedioxy-5,5',6,7-tetramethoxyflavone: [M+FA]-	-5.5	$C_{20}H_{18}O_8$	-	-	-	-	1.4E+04	-	4.6E+03	-
92	579.1371	2.53	Isocarlinoside (Luteolin 6-*C*-arabinoside 8-*C*-glucoside): [M-H]-: 0.471;Carlinoside (Luteolin 6-*C*-D-glucopyranoside-8-*C*-L-arabinopyranoside): [M-H]-: 0.471	2.7	$C_{26}H_{28}O_{15}$	-	-	-	-	-	-	3.6E+01	8.5E+03
93	595.1637	2.53	Vitexin 2''-*O*-glucopyranoside: [M-H]-: 0.310	-5.3	$C_{27}H_{32}O_{15}$	-	-	-	-	2.3E+01	-	-	1.5E+03
	595.1637	2.53	Tithonine: [2M-H]-: 0.358;Afrormosin/Afromosin (7-Hydroxy-4',6-dimethoxyisoflavone) : [2M-H]-: 0.358;8-*O*-methylretusin: [2M-H]-	4.6	$C_{17}H_{14}O_5$	-	-	-	-	2.3E+01	-	-	1.5E+03
94	463.0869	2.54	Hyperin (Quercetin-3-*O*-D-galactoside): [M-H]-: 0.494;Isoquercitrin (Quercetin-3-*O*-D-glucoside): [M-H]-: 0.494;Myricetrin (3-*O*-rhamnosylmyricetin): [M-H]-: 0.494;Quercetin-3-*O*-allopyranoside: [M-H]-: 0.494;Myricetin-3-*O*-L-rhamnopyranoside: [M-H]-: 0.494	-2.8	$C_{21}H_{20}O_{12}$	-	-	2.6E+03	3.3E+04	4.9E+02	7.4E+04	4.2E+03	1.5E+03
95	319.0809	2.54	Ourateacatechin (4'-Methyl-epigallocatechin): [M-H]-: 0.489	-4.3	$C_{16}H_{16}O_7$	-	-	4.2E+01	2.3E+02	-	6.2E+02	-	-
96	577.1579	2.54	Kaempferitrin (Kaempferol 3,7-*O*-di-rhamnoside): [M-H]-: 0.462	2.9	$C_{27}H_{30}O_{14}$	-	-	-	-	3.6E+02	-	7.8E+01	-
97	301.0327	2.55	Quercetin: [M-H]-: 0.362	-8.8	$C_{15}H_{10}O_7$	-	-	4.1E+01	1.5E+03	-	5.6E+02	5.7E+01	1.9E+01

98	927.1850	2.56	Hyperin (Quercetin-3-*O*-D-galactoside): [2M-H]-: 0.498;Isoquercitrin (Quercetin-3-*O*-D-glucoside): [2M-H]-: 0.498;Myricetrin (3-*O*-rhamnosylmyricetin): [2M-H]-: 0.498;Quercetin-3-*O*-allopyranoside: [2M-H]-: 0.498;Myricetin-3-*O*-L-rhamnopyranoside: [2M-H]-: 0.498	1.4	$C_{21}H_{20}O_{12}$	-	-	8.6E+00	1.2E+03	-	7.7E+03	1.7E+01	-
99	461.1095	2.56	Sorbifolin 6-*O*-glucopyranoside: [M-H]-: 0.569;7-*O*-Methoxyquercitrin: [M-H]-: 0.569;Quercetin-3'-*O*-methyl-3-*O*-D-rhamnopyranoside: [M-H]-: 0.569;Tectoridin: [M-H]-: 0.569	1.3	$C_{22}H_{22}O_{11}$	-	-	-	-	6.5E+02	-	-	-
	461.1095	2.56	3,5,7-triacetylcatechin: [M+FA]- 0.569	1.3	$C_{21}H_{20}O_{9}$	-	-	-	-	6.5E+02	-	-	-
100	593.1514	2.58	Nicotiflorin (Kaempferol-3-*O*-rutinoside): [M-H]-: 0.602;Kaempferol-3-*O*-L-rhamnopyranosyl-(1-6)-D-galactopyranoside: [M-H]-: 0.602	0.4	$C_{27}H_{30}O_{15}$	-	-	4.5E+01	-	1.0E+03	1.6E+01	7.3E+03	1.0E+04
101	463.0867	2.59	Hyperin (Quercetin-3-*O*-D-galactoside): [M-H]-: 0.478;Isoquercitrin (Quercetin-3-*O*-D-glucoside): [M-H]-: 0.478;Myricetrin (3-*O*-rhamnosylmyricetin): [M-H]-: 0.478;Quercetin-3-*O*-allopyranoside: [M-H]-: 0.478;Myricetin-3-*O*-L-rhamnopyranoside: [M-H]-: 0.478	-3.2	$C_{21}H_{20}O_{12}$	-	-	1.2E+03	1.1E+04	-	1.5E+04	6.2E+02	3.1E+03
102	447.0930	2.60	Orientin (Luteolin 8-*C*-glucoside): [M-H]-: 0.597;Quercitrin (3-*O*-rhamnosylquercetin): [M-H]-: 0.597;Trifolin (Kaempferol-3-*O*-D-galactopyranoside): [M-H]-: 0.597;Kaempferol-3-*O*-D-glucopyranoside: [M-H]-: 0.597;Isoorientin (Luteolin-6-glucoside): [M-H]-: 0.597;Quercetin-3-*O*-L-rhamnopyranoside: [M-H]-: 0.597	-0.7	$C_{21}H_{20}O_{11}$	-	-	-	7.4E+00	6.4E+02	5.8E+01	-	-

103	575.1193	2.61	(-)-Eriodictyol (2-(3,4-dihydroxyphenyl)-2,3-dihydro-5,7-dihydroxy-4H-chromen-4-one): [2M-H]-: 0.614	-0.3	C15H12O6	-	-	4.5E+02	2.0E+02	-	4.4E+02	1.1E+03	-
104	461.1081	2.62	Sorbifolin 6-*O*-glucopyranoside: [M-H]-: 0.547;7-*O*-Methoxyquercitrin: [M-H]-: 0.547;Quercetin-3'-*O*-methyl-3-*O*-D-rhamnopyranoside: [M-H]-: 0.547;Tectoridin: [M-H]-: 0.547	-1.8	C22H22O11	-	-	-	-	6.2E+02	-	-	-
105	579.1376	2.63	Isocarlinoside (Luteolin 6-*C*-arabinoside 8-*C*-glucoside): [M-H]-: 0.422;Carlinoside (Luteolin 6-*C*-D-glucopyranoside-8-*C*-L-arabinopyranoside): [M-H]-: 0.422	3.6	C26H28O15	-	-	-	-	-	7.7E+01	2.4E+01	1.6E+04
106	579.1376	2.65	Isocarlinoside (Luteolin 6-*C*-arabinoside 8-*C*-glucoside): [M-H]-: 0.423;Carlinoside (Luteolin 6-*C*-D-glucopyranoside-8-*C*-L-arabinopyranoside): [M-H]-: 0.423	3.6	C26H28O15	-	-	-	-	-	8.7E+01	2.4E+01	8.6E+03
107	593.1517	2.65	Nicotiflorin (Kaempferol-3-*O*-rutinoside): [M-H]-: 0.580;Kaempferol-3-*O*-L-rhamnopyranosyl-(1-6)-D-galactopyranoside: [M-H]-: 0.580	0.9	C27H30O15	-	-	7.5E+01	3.6E+02	2.6E+02	7.1E+03	2.0E+04	1.4E+04
108	595.1578	2.65	Tithonine: [2M-H]-: 0.310;Afrormosin/Afromosin (7-Hydroxy-4',6-dimethoxyisoflavone) : [2M-H]-: 0.310;8-*O*-methylretusin: [2M-H]-: 0.310	-5.4	C17H14O5	-	-	-	2.2E+01	-	1.9E+02	8.8E+02	4.9E+02
109	461.1082	2.66	Sorbifolin 6-*O*-glucopyranoside: [M-H]-: 0.563;7-*O*-Methoxyquercitrin: [M-H]-: 0.563;Quercetin-3'-*O*-methyl-3-*O*-D-rhamnopyranoside: [M-H]-: 0.563;Tectoridin: [M-H]-: 0.563	-1.5	C22H22O11	-	-	-	-	8.6E+02	-	-	-
110	609.1828	2.67	Hesperidin (Hesperetin 7-*O*-rutinoside): [M-H]-: 0.600	0.5	C28H34O15	-	-	-	-	-	-	4.0E+02	-

111	609.1481	2.70	Rutin (Quercetin-3-*O*-rutinoside): [M-H]-: 0.435;Quercetin-3-*O*-L-rhamnopyranosyl-(1-6)-D-galactopyranoside: [M-H]-: 0.435	3.3	C27H30O16	-	-	-	5.5E+00	-	1.4E+02	-	2.5E+02
112	433.0762	2.71	Reynoutrin (Quercetin-3-*O*-D-xylopyranoside): [M-H]-: 0.495;Quercetin-3-*O*-L-arabinopyranoside; guiajaverin: [M-H]-: 0.495	-3.2	C20H18O11	-	-	1.6E+03	3.5E+03	4.5E+02	2.6E+04	-	6.9E+03
113	595.1567	2.72	Tithonine: [2M-H]-: 0.206;Afrormosin/Afromosin (7-Hydroxy-4',6-dimethoxyisoflavone) : [2M-H]-: 0.206;8-*O*-methylretusin: [2M-H]-: 0.206	-7.2	C17H14O5	-	-	-	1.9E+02	-	4.2E+02	1.5E+02	1.0E+03
114	593.1514	2.72	Nicotiflorin (Kaempferol-3-*O*-rutinoside): [M-H]-: 0.609;Kaempferol-3-*O*-L-rhamnopyranosyl-(1-6)-D-galactopyranoside: [M-H]-: 0.609	0.4	C27H30O15	-	-	1.2E+01	4.2E+03	-	8.5E+03	2.9E+03	1.7E+04
115	579.1356	2.75	Isocarlinoside (Luteolin 6-*C*-arabinoside 8-*C*-glucoside): [M-H]-: 0.631;Carlinoside (Luteolin 6-*C*-D-glucopyranoside-8-*C*-L-arabinopyranoside): [M-H]-: 0.631	0.1	C26H28O15	-	-	-	7.3E+00	-	1.3E+02	-	1.1E+04
116	867.1640	2.75	Reynoutrin (Quercetin-3-*O*-D-xylopyranoside): [2M-H]-: 0.500;Quercetin-3-*O*-L-arabinopyranoside; guiajaverin: [2M-H]-: 0.500	1.6	C20H18O11	-	-	-	2.1E+02	-	8.1E+03	1.6E+01	3.2E+01
117	447.0930	2.76	Orientin (Luteolin 8-*C*-glucoside): [M-H]-: 0.611;Quercitrin (3-*O*-rhamnosylquercetin): [M-H]-: 0.611;Trifolin (Kaempferol-3-*O*-D-galactopyranoside): [M-H]-: 0.611;Kaempferol-3-*O*-D-glucopyranoside: [M-H]-: 0.611;Isoorientin (Luteolin-6-glucoside): [M-H]-: 0.611;Quercetin-3-*O*-L-rhamnopyranoside: [M-H]-: 0.611	-0.6	C21H20O11	-	-	4.1E+03	6.3E+03	-	7.4E+03	3.2E+03	-

118	433.0764	2.76	Reynoutrin (Quercetin-3-*O*-D-xylopyranoside): [M-H]-: 0.509;Quercetin-3-*O*-L-arabinopyranoside; guiajaverin: [M-H]-: 0.509	-2.9	C20H18O11	-	-	2.9E+03	9.8E+03	-	7.6E+04	2.8E+03	3.6E+03
119	623.1631	2.77	Nitensoside B (Pedalitin 6-*O*-rhamnopyranosyl(1'''6'')-glucopyranoside): [M-H]-: 0.503	2.2	C28H32O16	-	-	-	7.7E+01	-	-	2.0E+04	2.0E+01
120	575.1195	2.79	(-)-Eriodictyol (2-(3,4-dihydroxyphenyl)-2,3-dihydro-5,7-dihydroxy-4H-chromen-4-one): [2M-H]-: 0.635	0.1	C15H12O6	-	-	1.8E+02	5.2E+01	-	2.7E+02	4.2E+02	-
121	593.1522	2.80	Nicotiflorin (Kaempferol-3-*O*-rutinoside): [M-H]-: 0.537;Kaempferol-3-*O*-L-rhamnopyranosyl-(1-6)-D-galactopyranoside: [M-H]-: 0.537	1.7	C27H30O15	-	-	-	-	2.1E+01	4.9E+01	-	1.1E+03
122	579.1363	2.82	Isocarlinoside (Luteolin 6-*C*-arabinoside 8-*C*-glucoside): [M-H]-: 0.568;Carlinoside (Luteolin 6-*C*-D-glucopyranoside-8-*C*-L-arabinopyranoside): [M-H]-: 0.568	1.3	C26H28O15	-	-	8.5E+00	-	-	3.5E+02	2.8E+01	1.1E+04
123	593.1524	2.83	Nicotiflorin (Kaempferol-3-*O*-rutinoside): [M-H]-: 0.515;Kaempferol-3-*O*-L-rhamnopyranosyl-(1-6)-D-galactopyranoside: [M-H]-: 0.515	2.1	C27H30O15	-	-	-	-	9.6E+00	5.6E+01	8.1E+02	1.7E+03
124	269.0443	2.84	Apigenin: [M-H]-: 0.520;3',4',7-Trihydroxyflavone; 2-(3,4-dihydroxyphenyl)-7-hydroxy-4H-chromen-4-one: [M-H]-: 0.520;Sulfuretin [(2Z)-2-(3,4-dihydroxybenzylidene)-6-hydroxy-1-benzofuran-3(2H)-one]: [M-H]-: 0.520;3,7,4'-trihydroxyflavone: [M-H]-: 0.520	-4.5	C15H10O5	-	-	-	-	-	-	3.3E+02	-
125	375.0744	2.84	Apuleisin: [M-H]-: 0.421	5.9	C18H16O9	-	-	-	-	2.7E+02	-	-	-
126	341.0653	2.85	5-Hydroxy-3',4'-methylenedioxy-6,7-dimethoxyflavone: [M-H]-: 0.508	-4.0	C18H14O7	-	-	5.7E+02	1.1E+01	-	7.6E+01	-	-

127	497.1672	2.85	11-dodecyl-11,12-dihydro-5,10-dioxatetraphen-12-one: [M+Br]-: 0.395	-5.0	C28H34O3	-	-	-	-	5.6E+02	-	-	-
128	599.1178	2.85	Schaftoside (Apigenin-6-glucoside-8-arabinoside): [M+Cl]-: 0.591;Isoschaftoside (Apigenin-6-arabnoside-8-glucoside): [M+Cl]-: 0.591	0.8	C26H28O14	-	-	-	-	-	-	-	7.3E+02
129	563.1426	2.86	Schaftoside (Apigenin-6-glucoside-8-arabinoside): [M-H]-: 0.442;Isoschaftoside (Apigenin-6-arabnoside-8-glucoside): [M-H]-: 0.442	3.5	C26H28O14	-	-	-	-	6.6E+00	-	2.1E+02	1.4E+05
130	447.0927	2.87	Orientin (Luteolin 8-*C*-glucoside): [M-H]-: 0.588;Quercitrin (3-*O*-rhamnosylquercetin): [M-H]-: 0.588;Trifolin (Kaempferol-3-*O*-D-galactopyranoside): [M-H]-: 0.588;Kaempferol-3-*O*-D-glucopyranoside: [M-H]-: 0.588;Isoorientin (Luteolin-6-glucoside): [M-H]-: 0.588;Quercetin-3-*O*-L-rhamnopyranoside: [M-H]-: 0.588	-1.2	C21H20O11	-	5.5E+00	2.0E+04	2.7E+04	5.8E+02	8.6E+04	9.1E+03	1.8E+04
131	895.1971	2.87	Orientin (Luteolin 8-*C*-glucoside): [2M-H]-: 0.320;Quercitrin (3-*O*-rhamnosylquercetin): [2M-H]-: 0.320;Trifolin (Kaempferol-3-*O*-D-galactopyranoside): [2M-H]-: 0.320;Kaempferol-3-*O*-D-glucopyranoside: [2M-H]-: 0.320;Isoorientin (Luteolin-6-glucoside): [2M-H]-: 0.320;Quercetin-3-*O*-L-rhamnopyranoside: [2M-H]-: 0.320	3.6	C21H20O11	-	-	1.7E+03	2.7E+03	-	2.1E+04	8.4E+01	3.0E+02
132	477.1042	2.88	Pedalin (pedalitin 6-*O*-glucopyranoside): [M-H]-: 0.616	0.6	C22H22O12	-	-	1.5E+01	7.7E+01	1.0E+01	6.2E+01	2.5E+03	5.2E+01
133	449.0987	2.88	(2R)-7,4'-Diacetate-5-Hydroxy-3'-Methoxy-6,8-Dimethylflavanone: [M+Cl]-: 0.431	-4.7	C22H22O8	-	-	7.9E+02	8.3E+02	-	2.8E+03	2.3E+02	7.9E+01

134	431.0965	2.89	Afzelin (Kaempferol 3-*O*-rhamnoside): [M-H]-: 0.456	-4.4	C21H20O10	-	-	-	-	4.6E+02	-	-	-
	431.0965	2.89	3',4'-methylenedioxy-5,5',6,7-tetramethoxyflavone: [M+FA]-: 0.458	-4.3	C20H18O8	-	-	-	-	4.6E+02	-	-	-
135	377.0752	2.90	Tetra-Omethylscutellarein (4',5,6,7-tetramethoxyflavone): [M+Cl]-: 0.192;4',5,7,8-tetramethoxyflavone: [M+Cl]-: 0.192;(5aR,11aR)-3,8,9-trimethoxy-5a,10,11a,12-tetrahydro-5,11-dioxatetraphen-12-one: [M+Cl]-: 0.192;3',4',6,7-Tetramethoxyisoflavone: [M+Cl]-: 0.192	-12.0	C19H18O6	-	-	-	-	-	-	3.8E+02	-
136	375.0738	2.91	Apuleisin: [M-H]-: 0.486	4.3	C18H16O9	-	-	-	-	-	2.1E+01	8.9E+03	-
137	477.1047	2.92	Pedalin (pedalitin 6-*O*-glucopyranoside): [M-H]-: 0.568	1.7	C22H22O12	-	-	2.2E+01	1.4E+02	-	6.6E+02	6.0E+02	2.3E+01
138	593.1529	2.95	Nicotiflorin (Kaempferol-3-*O*-rutinoside): [M-H]-: 0.479;Kaempferol-3-*O*-L-rhamnopyranosyl-(1-6)-D-galactopyranoside: [M-H]-: 0.479	2.8	C27H30O15	-	-	8.4E+00	-	-	1.3E+02	3.6E+01	5.3E+02
139	405.0849	2.97	Oxyayanin-A: [M+FA]-: 0.429;Apuleidin: [M+FA]-: 0.429;Oxyayanin-B: [M+FA]-: 0.429;4'-*O*-Demethylgardenin D (5,3',4'-trihydroxy-6,7,8-trimethoxyflavone): [M+FA]-: 0.429	5.5	C18H16O8	-	-	-	-	-	-	2.6E+02	-
140	581.2607	2.98	11-octadecyl-11,12-dihydro-5,10-dioxatetraphen-12-one: [M+Br]-: 0.359	-5.0	C34H46O3	-	-	-	-	3.4E+02	-	1.3E+01	-
141	461.1086	2.99	Sorbifolin 6-*O*-glucopyranoside: [M-H]-: 0.621;7-*O*-Methoxyquercitrin: [M-H]-: 0.621;Quercetin-3'-*O*-methyl-3-*O*-D-rhamnopyranoside: [M-H]-: 0.621;Tectoridin: [M-H]-: 0.621	-0.6	C22H22O11	-	-	-	-	2.6E+02	-	-	3.0E+01

142	317.0652	3.03	(-)-Butin (2-(3,4-dihydroxyphenyl)-2,3-dihydro-7-hydroxy-4H-chromen-4-one): [M+FA]-: 0.506;Naringenin: [M+FA]-: 0.506	-4.6	$C_{15}H_{12}O_5$	-	-	9.4E+01	4.3E+01	-	5.1E+02	7.7E+01	-
143	341.0655	3.04	5-Hydroxy-3',4'-methylenedioxy-6,7-dimethoxyflavone: [M-H]-: 0.533	-3.5	$C_{18}H_{14}O_7$	-	-	2.1E+02	1.3E+01	-	4.6E+02	1.7E+01	-
144	447.0930	3.06	Orientin (Luteolin 8-*C*-glucoside): [M-H]-: 0.622;Quercitrin (3-*O*-rhamnosylquercetin): [M-H]-: 0.622;Trifolin (Kaempferol-3-*O*-D-galactopyranoside): [M-H]-: 0.622;Kaempferol-3-*O*-D-glucopyranoside: [M-H]-: 0.622;Isoorientin (Luteolin-6-glucoside): [M-H]-: 0.622;Quercetin-3-*O*-L-rhamnopyranoside: [M-H]-: 0.622	-0.7	$C_{21}H_{20}O_{11}$	-	-	5.9E+01	5.2E+02	2.4E+01	3.4E+02	4.2E+01	-
145	599.1178	3.06	Schaftoside (Apigenin-6-glucoside-8-arabinoside): [M+Cl]-: 0.604;Isoschaftoside (Apigenin-6-arabnoside-8-glucoside): [M+Cl]-: 0.604	0.8	$C_{26}H_{28}O_{14}$	-	-	-	-	-	-	-	9.2E+02
146	563.1412	3.06	Schaftoside (Apigenin-6-glucoside-8-arabinoside): [M-H]-: 0.595;Isoschaftoside (Apigenin-6-arabnoside-8-glucoside): [M-H]-: 0.595	1.0	$C_{26}H_{28}O_{14}$	-	-	-	-	1.1E+01	6.6E+01	5.5E+01	9.9E+04
147	447.0929	3.08	Orientin (Luteolin 8-*C*-glucoside): [M-H]-: 0.613;Quercitrin (3-*O*-rhamnosylquercetin): [M-H]-: 0.613;Trifolin (Kaempferol-3-*O*-D-galactopyranoside): [M-H]-: 0.613;Kaempferol-3-*O*-D-glucopyranoside: [M-H]-: 0.613;Isoorientin (Luteolin-6-glucoside): [M-H]-: 0.613;Quercetin-3-*O*-L-rhamnopyranoside: [M-H]-: 0.613	-0.9	$C_{21}H_{20}O_{11}$	-	-	5.0E+01	5.7E+02	1.4E+01	8.4E+02	1.4E+03	2.1E+01
148	405.1673	3.09	Lupinifolin: [M-H]-: 0.307;Erythrisenegalone: [M-H]-: 0.307	-8.5	$C_{25}H_{26}O_5$	-	-	2.3E+02	6.2E+00	-	2.0E+02	-	-

149	577.1570	3.11	Kaempferitrin (Kaempferol 3,7-*O*-di-rhamnoside): [M-H]-: 0.587	1.2	C27H30O14	-	-	-	-	-	8.2E+00	2.8E+01	3.0E+03
150	431.0964	3.11	Afzelin (Kaempferol 3-*O*-rhamnoside): [M-H]-: 0.453	-4.7	C21H20O10	-	-	-	1.4E+01	1.2E+03	-	-	-
	431.0964	3.11	3',4'-methylenedioxy-5,5',6,7-tetramethoxyflavone: [M+FA]-: 0.454;	-4.7	C20H18O8	-	-	-	1.4E+01	1.2E+03	-	-	-
151	405.0850	3.12	Oxyayanin-A: [M+FA]-: 0.431;Apuleidin: [M+FA]-: 0.431;Oxyayanin-B: [M+FA]-: 0.431;4'-*O*-Demethylgardenin D (5,3',4'-trihydroxy-6,7,8-trimethoxyflavone): [M+FA]-: 0.431	5.6	C18H16O8	-	-	-	-	-	-	5.0E+03	-
152	407.0858	3.12	Tangeretin (4',5,6,7,8-pentamethoxyflavone): [M+Cl]-: 0.209;Sinensetin (3',4',5,6,7-pentamethoxyflavone): [M+Cl]-: 0.209;5-demethoxynobiletin (3',4',6,7,8-pentamethoxyflavone): [M+Cl]-: 0.209;3',4',5',5,7-pentamethoxyflavone: [M+Cl]-: 0.209;3',4',5,7,8-pentamethoxyflavone: [M+Cl]-: 0.209;2',3',4',6,7-Pentamethoxyisoflavone: [M+Cl]-: 0.209	-11.0	C20H20O7	-	-	-	-	-	-	2.5E+02	-
153	651.1944	3.17	Isoswertisin-4'-methyl-ether-2''-L-rhamnoside: [M+FA]-: 0.529	2.0	C29H34O14	-	-	-	-	4.4E+02	-	-	3.6E+01
154	477.1031	3.18	Pedalin (pedalitin 6-*O*-glucopyranoside): [M-H]-: 0.585	-1.5	C22H22O12	-	-	1.3E+03	1.5E+02	-	1.1E+03	1.9E+02	1.5E+02
	477.1031	3.18	Afzelin (Kaempferol 3-*O*-rhamnoside): [M+FA]-: 0.587	-1.5	C21H20O10	-	-	1.3E+03	1.5E+02	-	1.1E+03	1.9E+02	1.5E+02
155	285.0383	3.18	Kaempferol: [M-H]-: 0.443;Luteolin: [M-H]-: 0.443	-7.6	C15H10O6	-	-	1.8E+03	1.5E+02	-	1.2E+03	1.8E+02	1.5E+02
156	863.2053	3.18	Afzelin (Kaempferol 3-*O*-rhamnoside): [2M-H]-: 0.535	1.4	C21H20O10	-	-	5.1E+03	3.7E+01	-	2.2E+03	6.3E+01	2.2E+01

157	467.0744	3.18	Afzelin (Kaempferol 3-*O*-rhamnoside): [M+Cl]-: 0.592;(+)-Pinitol: [2M+Br]-: 0.401	-1.4	C21H20O10	-	-	2.9E+02	5.1E+01	-	1.9E+02	6.7E+01	4.4E+01
158	431.0971	3.18	Afzelin (Kaempferol 3-*O*-rhamnoside): [M-H]-: 0.533	-2.9	C21H20O10	-	-	4.8E+04	5.5E+03	2.8E+01	4.0E+04	6.8E+03	5.0E+03
	431.0971	3.18	3',4'-methylenedioxy-5,5',6,7-tetramethoxyflavone: [M+FA]-: 0.534	-2.9	C20H18O8	-	-	4.8E+04	5.5E+03	2.8E+01	4.0E+04	6.8E+03	5.0E+03
159	613.1354	3.19	Farnisine: [2M+FA]-: 0.637;Acacetin: [2M+FA]-: 0.637;Biochanin A; 5,7-dihydroxy-4-methoxyisoflavone: [2M+FA]-: 0.637;3'-Hydroxyformononetin: [2M+FA]-: 0.637;Izalpinin (3,5-dihydroxy-7-methoxyflavone): [2M+FA]-: 0.637;Texasin (6,7-dihydroxy-4'-methoxyisoflavone): [2M+FA]-: 0.637;(+)-Maackiain [(6aS,11aS)-3-hydroxy-8,9-methylenedioxypterocarpan]: [2M+FA]-: 0.637;(-)-Maackiain [(6aR,11aR)-3-hydroxy-8,9-methylenedioxypterocarpan]: [2M+FA]-: 0.637;Kaempferitrin (Kaempferol 3,7-*O*-di-rhamnoside): [M+Cl]-: 0.418	0.4	C16H12O5	-	-	5.9E+02	-	-	-	-	-
160	433.0784	3.22	Reynoutrin (Quercetin-3-*O*-D-xylopyranoside): [M-H]-: 0.582;Quercetin-3-*O*-L-arabinopyranoside; guiajaverin: [M-H]-: 0.582	1.9	C20H18O11	-	-	-	-	-	2.9E+02	-	-
161	431.0982	3.25	Afzelin (Kaempferol 3-*O*-rhamnoside): [M-H]-: 0.647	-0.4	C21H20O10	-	-	2.1E+02	6.8E+02	-	2.9E+02	1.0E+02	2.7E+01
	431.0982	3.25	3',4'-methylenedioxy-5,5',6,7-tetramethoxyflavone: [M+FA]-: 0.648	-0.4	C20H18O8	-	-	2.1E+02	6.8E+02	-	2.9E+02	1.0E+02	2.7E+01

162	461.1089	3.25	Sorbifolin 6-*O*-glucopyranoside: [M-H]-: 0.659;7-*O*-Methoxyquercitrin: [M-H]-: 0.659;Quercetin-3'-*O*-methyl-3-*O*-D-rhamnopyranoside: [M-H]-: 0.659;Tectoridin: [M-H]-: 0.659	-0.1	C22H22O11	-	-	2.7E+02	6.1E+01	-	7.9E+02	6.4E+02	1.6E+01
163	575.1199	3.25	(-)-Eriodictyol (2-(3,4-dihydroxyphenyl)-2,3-dihydro-5,7-dihydroxy-4H-chromen-4-one): [2M-H]-: 0.627	0.6	C15H12O6	-	-	1.4E+02	1.8E+01	-	1.7E+03	8.4E+01	-
164	757.1777	3.27	3',4'-methylenedioxy-5,6,7-trimethoxyflavone: [2M+FA]-: 0.635;3',4'-methylenedioxy-5,7,8-trimethoxyflavone: [2M+FA]-: 0.635;2',6,7-Trimethoxy-4',5'-Methylenedioxyisoflavone: [2M+FA]-: 0.635	0.4	C19H16O7	-	-	6.1E+02	-	-	-	-	-
165	505.1346	3.28	5-*O*-Acetyl-2',3',6-Tri-*O*-Methylapuleisin: [M+FA]-: 0.607	-1.1	C23H24O10	-	-	-	-	8.0E+02	-	-	-
166	405.1184	3.29	(6aR,11aR)-8-Hydroxy-3,4,9,10-tetramehtoxypterocarpan: [M+FA]-: 0.589	-1.8	C19H20O7	-	-	-	1.5E+01	-	7.0E+01	4.7E+02	6.3E+00
167	403.1398	3.31	3',4',5',5,6,7,-Hexamethoxyflavanone: [M-H]-: 0.662	-0.1	C21H24O8	-	-	8.1E+01	-	-	3.8E+02	6.1E+01	2.7E+01
	403.1398	3.31	(2R)-5-Hydroxy-7,3',4'-Trimethoxy-6,8-Dimethylflavanone: [M+FA]-: 0.664;(2R)-7-Ethoxy-5,4'-Dihydroxy-3'-Methoxy-6,8-Dimethylflavanone: [M+FA]-: 0.664	-0.1	C20H22O6	-	-	8.1E+01	-	-	3.8E+02	6.1E+01	2.7E+01
168	757.1797	3.32	3',4'-methylenedioxy-5,6,7-trimethoxyflavone: [2M+FA]-: 0.440;3',4'-methylenedioxy-5,7,8-trimethoxyflavone: [2M+FA]-: 0.440;2',6,7-Trimethoxy-4',5'-Methylenedioxyisoflavone: [2M+FA]-: 0.440	3.0	C19H16O7	-	-	3.6E+02	-	-	-	-	-

169	433.0758	3.34	Reynoutrin (Quercetin-3-*O*-D-xylopyranoside): [M-H]-: 0.485;Quercetin-3-*O*-L-arabinopyranoside; guiajaverin: [M-H]-: 0.485	-4.2	$C_{20}H_{18}O_{11}$	-	-	-	-	-	3.0E+02	-	-
170	875.2069	3.37	Alpinumisoflavone diacetate: [2M+Cl]-: 0.231	-5.0	$C_{24}H_{20}O_{7}$	-	-	-	-	4.3E+02	-	-	-
171	613.1357	3.37	Farnisine: [2M+FA]-: 0.614;Acacetin: [2M+FA]-: 0.614;Biochanin A; 5,7-dihydroxy-4-methoxyisoflavone: [2M+FA]-: 0.614;3'-Hydroxyformononetin: [2M+FA]-: 0.614;Izalpinin (3,5-dihydroxy-7-methoxyflavone): [2M+FA]-: 0.614;Texasin (6,7-dihydroxy-4'-methoxyisoflavone): [2M+FA]-: 0.614;(+)-Maackiain [(6aS,11aS)-3-hydroxy-8,9-methylenedioxypterocarpan]: [2M+FA]-: 0.614;(-)-Maackiain [(6aR,11aR)-3-hydroxy-8,9-methylenedioxypterocarpan]: [2M+FA]-: 0.614;Kaempferitrin (Kaempferol 3,7-*O*-di-rhamnoside): [M+Cl]-: 0.395	0.9	$C_{16}H_{12}O_{5}$	-	-	7.0E+02	-	-	-	-	-
172	431.0964	3.38	Afzelin (Kaempferol 3-*O*-rhamnoside): [M-H]-: 0.475	-4.5	$C_{21}H_{20}O_{10}$	-	-	4.9E+01	2.3E+02	-	6.6E+01	5.2E+00	-
	431.0964	3.38	3',4'-methylenedioxy-5,5',6,7-tetramethoxyflavone: [M+FA]-: 0.476	-4.5	$C_{20}H_{18}O_{8}$	-	-	4.9E+01	2.3E+02	-	6.6E+01	5.2E+00	-

173	447.0929	3.41	Orientin (Luteolin 8-*C*-glucoside): [M-H]-: 0.634;Quercitrin (3-*O*-rhamnosylquercetin): [M-H]-: 0.634;Trifolin (Kaempferol-3-*O*-D-galactopyranoside): [M-H]-: 0.634;Kaempferol-3-*O*-D-glucopyranoside: [M-H]-: 0.634;Isoorientin (Luteolin-6-glucoside): [M-H]-: 0.634;Quercetin-3-*O*-L-rhamnopyranoside: [M-H]-: 0.634	-0.8	C21H20O11	-	-	-	-	-	3.8E+02	-	-
174	253.0484	3.47	7,4'-Dihydroxyflavone: [M-H]-: 0.449;Chrysin: [M-H]-: 0.449;3,7-dihydroxyflavone: [M-H]-: 0.449	-8.9	C15H10O4	-	-	-	-	-	2.1E+02	-	-
175	301.0340	3.60	Quercetin: [M-H]-: 0.544	-4.5	C15H10O7	-	-	1.5E+03	6.7E+02	-	5.8E+04	4.8E+02	6.7E+01
176	603.0783	3.60	Quercetin: [2M-H]-: 0.659	0.4	C15H10O7	-	-	7.2E+00	-	-	5.1E+03	-	-
177	317.0297	3.61	Myricetin: [M-H]-: 0.616	-2.0	C15H10O8	-	-	6.6E+00	-	-	8.9E+02	-	-
178	465.1440	4.00	Catechin-3-*O*-rhamnopyranoside: [M+FA]-: 0.323	8.1	C21H24O9	-	-	2.7E+01	-	-	-	2.2E+03	-
179	285.0388	4.12	Kaempferol: [M-H]-: 0.545;Luteolin: [M-H]-: 0.545	-5.7	C15H10O6	-	-	4.5E+03	3.0E+02	-	2.8E+04	8.5E+02	1.5E+03
180	357.1332	4.21	(2R)-5-Hydroxy-7,3',4'-Trimethoxy-6,8-Dimethylflavanone: [M-H]-: 0.594;(2R)-7-Ethoxy-5,4'-Dihydroxy-3'-Methoxy-6,8-Dimethylflavanone: [M-H]-: 0.594	-3.3	C20H22O6	-	-	1.4E+03	-	8.3E+00	-	1.5E+02	-
181	623.1427	4.27	Procyanidin B-3: [M+FA]-: 0.505;Proanthocyanidin: [M+FA]-: 0.505;Kaempferol-3-*O*-?-L-(4''-E-*p*-coumaroyl)-rhamnoside: [M+FA]-: 0.505;Kaempferol-3-*O*-?-L-(4''-Z-*p*-coumaroyl)-rhamnoside: [M+FA]-: 0.505	3.4	C30H26O12	-	-	-	-	-	-	-	3.6E+02
182	315.0496	4.28	Pedalitin: [M-H]-: 0.567;3-*O*-methylquercetin: [M-H]-: 0.567;Isorhamnetin: [M-H]-: 0.567	-4.6	C16H12O7	-	-	1.8E+01	1.5E+01	-	1.6E+03	3.8E+02	-

	315.0496	4.28	Apigenin: [M+FA]-: 0.568;3',4',7-Trihydroxyflavone; 2-(3,4-dihydroxyphenyl)-7-hydroxy-4H-chromen-4-one: [M+FA]-: 0.568;Sulfuretin [(2Z)-2-(3,4-dihydroxybenzylidene)-6-hydroxy-1-benzofuran-3(2H)-one]: [M+FA]-: 0.568;3,7,4'-trihydroxyflavone: [M+FA]-: 0.568	-4.6	C15H10O5	-	-	1.8E+01	1.5E+01	-	1.6E+03	3.8E+02	-
183	253.0483	4.34	7,4'-Dihydroxyflavone: [M-H]-: 0.484;Chrysin: [M-H]-: 0.484;3,7-dihydroxyflavone: [M-H]-: 0.484	-9.2	C15H10O4	-	-	-	-	-	1.2E+03	-	-
184	577.1348	4.35	Procyanidin B-3: [M-H]-: 0.683;Proanthocyanidin: [M-H]-: 0.683;Kaempferol-3-*O*-?-L-(4''-E-*p*-coumaroyl)-rhamnoside: [M-H]-: 0.683;Kaempferol-3-*O*-?-L-(4''-Z-*p*-coumaroyl)-rhamnoside: [M-H]-: 0.683	-0.5	C30H26O12	-	-	3.7E+01	1.0E+02	8.9E+02	3.6E+02	4.9E+01	3.7E+02
185	577.1346	4.43	Procyanidin B-3: [M-H]-: 0.658;Proanthocyanidin: [M-H]-: 0.658;Kaempferol-3-*O*-?-L-(4''-E-*p*-coumaroyl)-rhamnoside: [M-H]-: 0.658;Kaempferol-3-*O*-?-L-(4''-Z-*p*-coumaroyl)-rhamnoside: [M-H]-: 0.658	-1.0	C30H26O12	-	-	3.2E+02	3.3E+02	2.6E+03	7.3E+03	1.3E+03	3.0E+03
186	285.0394	4.43	Kaempferol: [M-H]-: 0.612;Luteolin: [M-H]-: 0.612	-3.9	C15H10O6	-	-	3.6E+01	1.5E+01	4.1E+01	2.7E+02	4.7E+01	9.9E+01
187	577.1352	4.48	Procyanidin B-3: [M-H]-: 0.720;Proanthocyanidin: [M-H]-: 0.720;Kaempferol-3-*O*-?-L-(4''-E-*p*-coumaroyl)-rhamnoside: [M-H]-: 0.720;Kaempferol-3-*O*-?-L-(4''-Z-*p*-coumaroyl)-rhamnoside: [M-H]-: 0.720	0.1	C30H26O12	-	-	1.4E+03	1.0E+03	-	1.9E+04	5.8E+03	1.3E+04
188	577.1353	4.59	Procyanidin B-3: [M-H]-: 0.716;Proanthocyanidin: [M-H]-: 0.716;Kaempferol-3-*O*-?-L-(4''-E-*p*-coumaroyl)-rhamnoside: [M-H]-: 0.716;Kaempferol-3-*O*-?-L-(4''-Z-*p*-coumaroyl)-rhamnoside: [M-H]-: 0.716	0.3	C30H26O12	-	-	1.9E+02	3.5E+02	5.1E+02	1.4E+03	7.0E+02	1.9E+03

189	315.1217	5.45	1-(2'-Hydroxy-4'-methoxy-5'-methylphenyl)-3-(2''-hydroxy-4'',5''-methylenedioxyphenyl)-propane: [M-H]-: 0.559;5-methoxy-2-(3-(5-methoxybenzo[d][1,3]dioxol-6-yl)propyl)phenol: [M-H]-: 0.559;4-(3-(5-hydroxybenzo[d][1,3]dioxol-6-yl)propyl)-2,6-dimethylbenzene-1,3-diol: [M-H]-: 0.559;6-(3-(2-hydroxy-4-methoxy-5-methylphenyl)propyl)benzo[d][1,3]dioxol-5-ol: [M-H]-: 0.559	-6.8	C18H20O5	-	-	-	-	1.3E+03	-	-	-
190	431.1709	5.55	(2R)-7,4'-Diethoxy-5-Hydroxy-3'-Methoxy-6,8-Dimethylflavanone: [M+FA]-: 0.752	-0.6	C22H26O6	-	-	2.6E+02	9.2E+01	-	3.3E+02	1.9E+01	9.7E+01
191	731.1721	5.67	3',4'-methylenedioxy-7,8-(2'',2''-dimethylpyrano)-flavone: [2M+Cl]-: 0.464	4.3	C21H16O5	-	-	-	-	-	6.3E+02	-	5.9E+01
192	447.1337	5.68	Nobiletin (3',4',5,6,7,8-hexamethoxyflavone): [M+FA]-: 0.378;5,6,7,3',4',5'-hexamethoxyflavone: [M+FA]-: 0.378;3',4',5',5,7,8-hexamethoxyflavone: [M+FA]-: 0.378;3,5,7,8,3',4'-hexamethoxyflavone: [M+FA]-: 0.378;Oxyayanin-A Trimethyl Ether: [M+FA]-: 0.378	9.1	C21H22O8	5.4E+00	1.5E+01	9.5E+01	1.4E+02	-	7.0E+01	6.4E+03	1.6E+02
193	671.2683	9.34	6-methoxy-6'',6''-dimethylchromeno-[2'',3'':7,8]-flavanone: [2M-H]-: 0.644	4.8	C21H20O4	6.3E+00	9.8E+00	4.9E+01	6.1E+01	5.0E+01	9.2E+01	5.6E+02	5.6E+02

(-); peak area equal 0.

Table S3. Common sugar types and neutral fragments

Monossacharides				Neutral losses (Da)			
**Sugar type**	**Common examples**	**MF**	**Monoisotopic Mass (Da)**	**Heterolytic *O*-cleavage**	**Homolytic *O*-cleavage**	***C*-cleavage 1**	***C*-cleavage 2**
Pentose	Arabinose, xylose	C5H10O5	150.0528	132.0423	133.0580	60.0211	90.0317
Deoxyhexose	Rhamnose, fucose	C6H12O5	164.0785	146.0579	147.0657	74.0367	104.0473
Aminosugar	Glucosamine, galactosamine	C6H13NO5	179.0794	161.0688	162.07663	89.0476	119.0582
Hexose	Glucose, galactose, mannose	C6H12O6	180.0634	162.0528	163.0606	90.0316	120.0422
Uronic acid	Glucuronic acid, galacturonic acid	C6H10O7	194.0427	176.0321	177.0399	104.0109	134.0215
N-Acetylaminosugar	N-acetylglucosamine, N-acetylgalactosamine	C8H15NO6	221.0899	203.0794	204.0872	131.0582	161.0688
**Dissacharides**							
Pentosyl-pentose		C10H18O9	282.0951	264.0845	265.0923	192.0633	222.0739

Deoxyhexosyl-pentose		C11H20O9	296.1107	278.1002	279.1080	206.0790	236.0896
Deoxyhexosyl-deoxyhexose		C12H22O9	310.1264	292.1158	293.1236	220.0946	250.1052
Hexosyl-pentoside		C11H20O10	312.1056	294.0951	295.1029	222.0739	252.0845
Hexosyl-deoxyhexose		C12H22O10	326.1213	308.1107	309.1186	236.0895	266.1001
Hexosyl-hexose		C12H22O11	342.1162	324.1056	325.1135	252.0844	282.0950
**Derivatives**							
Coumaroyl-pentose		C14H16O7	296.0896	278.0790	279.0869	206.0578	236.0684
Coumaroyl-deoxyhexose		C15H18O7	310.1052	292.0947	293.1025	220.0735	250.0841
Coumaroyl-hexose		C15H18O8	326.1002	308.0896	309.09743	236.0684	266.0790
Coumaroyl-pentosyl-pentose		C19H24O11	428.1318	410.1213	411.1291	338.1001	368.1107
Coumaroyl-deoxyhexosyl-pentose		C20H26O11	442.1475	424.1369	425.1447	352.1157	382.1263
Coumaroyl-deoxyhexosyl-deoxyhexose		C21H28O11	456.1632	438.1526	439.1604	366.1314	396.1420
Coumaroyl-hexosyl-pentose		C20H26O12	458.1424	440.1319	441.1397	368.1107	398.1213
Coumaroyl-hexosyl-deoxyhexose		C21H28O12	472.1581	454.1475	445.1553	382.1263	412.1369
Coumaroyl-hexosyl-hexose		C21H28O13	488.1530	470.1424	471.1502	398.1212	428.1318
Di-coumaroyl-pentose		C23H22O9	442.1264	424.1158	425.1236	352.0946	382.1052

Di-coumaroyl-deoxyhexose		C24H24O9	456.1420	438.13147	439.1393	366.1103	396.1209
Di-coumaroyl-hexose		C24H24O10	472.1369	454.1264	455.1342	382.1052	412.1158
Hydroxybenzoylcoumaroyl-pentose		C21H20O9	416.1107	398.1002	399.1080	326.0790	356.0896
Hydroxybenzoylcoumaroyl-deoxyhexose		C22H22O13	430.1264	412.1158	413.1236	340.0946	370.1052
Hydroxybenzoylcoumaroyl-hexose		C22H22O10	446.1213	428.1107	429.1186	356.0895	386.1001
Di-acetylcoumaroyl-pentoside		C18H20O9	380.1107	362.1002	363.1180	290.0790	320.0896
Di-acetylcoumaroyl-deoxyhexoside		C19H22O9	394.1264	376.1158	377.1236	304.0946	334.1052
Di-acetylcoumaroyl-hexoside		C19H22O10	410.1213	392.1108	393.1186	320.0896	350.1002

Table S4. Aglycone annotation based on MS[2] hits from *FlavAgly_DB*

N°	*m/z*	RT (min)	Annotated aglycone (name)	Error (ppm)	MS² blank replicates Peak area		MS² *O.notata* Peak area	MS² *O.guianensis* Peak area	MS² *O.porosa* Peak area	MS² *O.lancifolia* Peak area	MS² *O.diospyrifolia* Peak area	MS² *O.odorifera* Peak area
1	289.0710	1.63	Catechin/Epicatechin [M-H]-: 0.502	-2.6	-	-	2.4E+02	3.0E+02	3.0E+01	3.4E+01	-	2.4E+02
2	289.0711	1.70	Catechin/Epicatechin [M-H]-: 0.518	-2.3	-	-	5.2E+02	3.9E+02	5.9E+01	7.2E+01	-	2.9E+02
3	355.0821	1.80	Narigenin-di-etenol[M-H]-: 0.566	-0.6	-	-	-	-	1.5E+01	4.6E+01	2.1E+02	3.7E+01
4	385.0928	1.80	Narigenin-propenodiol + etenol [M-H]-: 0.581	-0.2	-	-	-	-	9.1E+00	3.8E+01	1.8E+02	2.2E+01
5	369.0612	1.87	Kaempferol / Luteolin / Datiscetin -di-etenol [M-H]-: 0.555	-1.1	-	-	-	-	2.9E+02	-	-	2.0E+01
6	269.0447	1.91	Apigenin [M-H]-: 0.506	-3.2	-	-	1.3E+01	2.7E+01	2.0E+01	2.8E+01	4.5E+02	1.2E+01
7	353.0672	1.91	Apigenin-di-etenol[M-H]-: 0.540	1.5	-	-	-	-	7.1E+03	-	7.3E+00	5.3E+03
8	355.0828	1.91	Narigenin-di-etenol[M-H]-: 0.548	1.3	-	-	-	-	-	2.3E+01	8.6E+01	-
9	383.0777	1.91	Apigenin-propenodiol + etenol [M-H]-: 0.549	1.2	-	-	-	-	4.1E+03	-	-	2.8E+03
10	385.0910	1.91	Narigenin-propenodiol + etenol [M-H]-: 0.407	-4.9	-	-	-	-	8.4E+00	1.5E+01	7.8E+01	1.7E+01
11	311.0555	1.92	Apigenin-etenol [M-H]-: 0.536	-2.0	-	-	-	1.4E+01	3.1E+02	-	2.6E+01	2.1E+02
12	413.0871	1.92	Apigenin-di-propenodiol [M-H]-: 0.529	-1.7	-	-	6.9E+01	5.4E+01	4.0E+02	-	-	3.4E+02
13	327.0501	1.94	Kaempferol / Luteolin / Datiscetin -etenol [M-H]-: 0.502	-2.8	-	-	-	4.2E+01	2.6E+02	1.8E+01	-	1.6E+01
14	285.0386	1.96	Kaempferol / Luteolin / Datiscetin [M-H]-: 0.415	-6.5	-	-	6.5E+02	1.0E+02	2.8E+01	6.7E+01	6.3E+01	5.7E+02
15	289.0706	1.96	Catechin/Epicatechin [M-H]-: 0.477	-4.0	-	-	2.7E+03	6.1E+02	5.1E+01	1.8E+02	-	-
16	399.0717	1.96	Kaempferol / Luteolin / Datiscetin-propenodiol + etenol [M-H]-: 0.552	-1.2	-	-	-	-	2.9E+02	-	-	4.3E+01

17	369.0604	1.97	Kaempferol / Luteolin / Datiscetin -di-etenol [M-H]-: 0.481	-3.2	-	-	-	-	3.6E+02	6.1E+00	-	5.9E+01
18	353.0670	1.98	Apigenin-di-etenol[M-H]-: 0.567	0.9	-	-	-	-	5.0E+03	-	-	-
19	413.0873	2.00	Apigenin-di-propenodiol [M-H]-: 0.548	-1.2	-	-	4.3E+01	7.0E+01	3.9E+02	-	-	-
20	289.0713	2.01	Catechin/Epicatechin [M-H]-: 0.556	-1.6	-	-	2.6E+03	6.1E+02	-	4.8E+02	-	5.8E+03
21	311.0553	2.01	Apigenin-etenol [M-H]-: 0.524	-2.6	-	-	1.5E+01	1.5E+01	3.1E+02	-	2.2E+01	1.7E+02
22	383.0778	2.01	Apigenin-propenodiol + etenol [M-H]-: 0.546	1.5	-	-	1.3E+01	1.5E+01	3.2E+03	-	-	1.7E+03
23	285.0390	2.02	Kaempferol / Luteolin / Datiscetin [M-H]-: 0.452	-5.1	-	-	1.2E+03	1.4E+02	7.9E+01	1.4E+02	1.1E+02	2.0E+03
24	269.0441	2.03	Apigenin [M-H]-: 0.455	-5.4	-	-	5.5E+01	2.2E+01	7.5E+00	2.1E+01	2.2E+02	-
25	353.0683	2.03	Apigenin-di-etenol[M-H]-: 0.439	4.6	-	-	-	-	5.8E+03	4.8E+00	5.4E+00	3.1E+03
26	289.0711	2.09	Catechin/Epicatechin [M-H]-: 0.539	-2.3	-	-	1.6E+03	7.1E+02	1.9E+01	2.8E+02	-	4.6E+03
27	327.0506	2.09	Kaempferol / Luteolin / Datiscetin -etenol [M-H]-: 0.562	-1.3	-	-	-	3.9E+01	3.7E+02	1.2E+01	-	1.2E+02
28	285.0389	2.10	Kaempferol / Luteolin / Datiscetin [M-H]-: 0.444	-5.5	-	-	5.1E+02	7.4E+01	7.5E+01	1.0E+02	8.1E+01	2.3E+03
29	413.0873	2.12	Apigenin-di-propenodiol [M-H]-: 0.553	-1.2	-	-	4.2E+01	7.7E+01	1.4E+03	-	-	3.0E+02
30	300.0264	2.13	Quercetin [M-H].-: 0.492	-3.8	-	-	2.6E+01	-	-	6.5E+01	7.0E+02	-
31	429.0830	2.13	Kaempferol / Luteolin / Datiscetin-di-propenodiol [M-H]-: 0.581;Myricetin-propenodiol + etenol [M-H]-: 0.581	0.7	-	-	-	-	4.1E+01	-	-	3.4E+02
32	353.0673	2.14	Apigenin-di-etenol[M-H]-: 0.547	1.8	-	-	-	-	1.3E+04	-	-	3.3E+03
33	383.0761	2.14	Apigenin-propenodiol + etenol [M-H]-: 0.497	-3.0	-	-	-	-	1.5E+04	-	-	3.5E+03
34	327.0513	2.15	Kaempferol / Luteolin / Datiscetin -etenol [M-H]-: 0.581	0.8	-	-	-	4.0E+01	3.8E+02	1.4E+01	-	8.0E+02

35	357.0606	2.15	Kaempferol / Luteolin / Datiscetin-propenodiol [M-H]-: 0.505	-2.8	-	-	-	-	4.8E+02	-	-	4.2E+02
36	447.0931	2.16	Taxifolin-di-propenodiol [M-H]-: 0.593	-0.4	-	-	-	-	2.9E+02	4.1E+01	1.2E+01	-
37	311.0555	2.18	Apigenin-etenol [M-H]-: 0.548	-2.0	-	-	-	3.1E+01	2.5E+03	-	-	3.2E+02
38	353.0673	2.18	Apigenin-di-etenol[M-H]-: 0.547	1.8	-	-	-	-	1.3E+04	-	-	4.7E+03
39	413.0866	2.18	Apigenin-di-propenodiol [M-H]-: 0.488	-2.9	-	-	1.2E+02	3.3E+01	1.0E+03	-	-	4.0E+02
40	429.0830	2.21	Kaempferol / Luteolin / Datiscetin-di-propenodiol [M-H]-: 0.582;Myricetin-propenodiol + etenol [M-H]-: 0.582	0.7	-	-	-	-	5.7E+01	-	-	2.3E+02
41	300.0269	2.22	Quercetin [M-H].-: 0.550	-2.2	-	-	1.6E+01	-	7.5E+01	3.2E+02	5.3E+02	2.2E+01
42	383.0776	2.22	Apigenin-propenodiol + etenol [M-H]-: 0.571	0.9	-	-	-	6.2E+00	9.8E+03	-	-	2.6E+03
43	327.0504	2.23	Kaempferol / Luteolin / Datiscetin -etenol [M-H]-: 0.552	-1.9	-	-	-	2.5E+01	2.8E+03	2.7E+01	-	5.2E+02
44	357.0606	2.23	Kaempferol / Luteolin / Datiscetin-propenodiol [M-H]-: 0.511	-2.8	-	-	-	-	1.1E+03	-	-	3.0E+02
45	447.0921	2.23	Taxifolin-di-propenodiol [M-H]-: 0.494	-2.7	-	-	-	-	9.9E+02	1.5E+01	-	-
46	301.0342	2.24	Quercetin [M-H]-: 0.492	-3.9	-	-	1.2E+02	1.2E+01	1.0E+02	3.0E+02	-	2.8E+01
47	353.0669	2.24	Apigenin-di-etenol[M-H]-: 0.587	0.6	-	-	-	-	1.3E+03	-	-	3.7E+03
48	300.0272	2.26	Quercetin [M-H].-: 0.582	-1.2	-	-	3.1E+01	-	8.6E+01	4.2E+02	7.5E+02	1.8E+01
49	355.0788	2.28	Narigenin-di-etenol[M-H]-: 0.259	-9.9	-	-	-	-	1.9E+02	-	-	5.7E+01
50	447.0930	2.28	Taxifolin-di-propenodiol [M-H]-: 0.589	-0.6	-	-	-	-	8.4E+02	3.6E+01	-	-
51	269.0445	2.29	Apigenin [M-H]-: 0.504	-3.9	-	-	2.3E+01	5.5E+01	9.5E+01	3.3E+01	2.5E+03	2.9E+02
52	341.0661	2.29	Apigenin-propenodiol [M-H]-: 0.557	-1.7	-	-	3.0E+01	1.1E+02	1.5E+02	-	-	4.2E+02
53	353.0675	2.29	Apigenin-di-etenol[M-H]-: 0.529	2.3	-	-	-	-	4.3E+03	-	-	7.3E+02
54	357.0609	2.29	Kaempferol / Luteolin / Datiscetin-propenodiol [M-H]-: 0.550	-1.9	-	-	-	-	7.4E+02	8.6E+00	-	2.7E+01
55	383.0783	2.29	Apigenin-propenodiol + etenol [M-H]-:	2.8	-	-	-	1.6E+01	2.9E+03	-	-	5.9E+02

			0.507									
56	413.0874	2.29	Apigenin-di-propenodiol [M-H]-: 0.569	-1.0	-	-	1.1E+02	3.9E+01	1.1E+03	-	-	1.7E+04
57	284.0319	2.30	Kaempferol / Luteolin / Datiscetin [M-H].-: 0.447	5.8	-	-	-	-	2.3E+02	3.5E+01	2.0E+03	2.0E+02
58	311.0560	2.30	Apigenin-etenol [M-H]-: 0.604	-0.4	-	-	-	-	3.2E+02	-	1.2E+01	2.0E+03
59	327.0505	2.32	Kaempferol / Luteolin / Datiscetin -etenol [M-H]-: 0.563	-1.6	-	-	2.8E+01	4.3E+01	3.2E+03	-	4.6E+01	3.2E+01
60	413.0870	2.34	Apigenin-di-propenodiol [M-H]-: 0.540	-2.0	-	-	1.1E+02	6.3E+01	1.7E+03	-	-	1.5E+04
61	353.0668	2.35	Apigenin-di-etenol[M-H]-: 0.603	0.3	-	-	-	-	4.3E+02	-	-	3.3E+02
62	269.0446	2.36	Apigenin [M-H]-: 0.524	-3.5	-	-	3.9E+01	3.5E+01	8.9E+01	5.2E+01	2.6E+03	2.6E+02
63	289.0708	2.37	Catechin/Epicatechin [M-H]-: 0.520	-3.3	-	-	4.3E+02	5.9E+03	2.7E+01	1.6E+02	-	1.4E+02
64	311.0555	2.37	Apigenin-etenol [M-H]-: 0.554	-2.0	-	-	-	2.6E+01	4.1E+02	-	2.1E+01	1.8E+03
65	341.0665	2.37	Apigenin-propenodiol [M-H]-: 0.600	-0.5	-	-	-	5.7E+01	1.7E+02	-	-	3.3E+02
66	300.0268	2.40	Quercetin [M-H].-: 0.543	-2.5	-	-	1.7E+01	9.3E+00	5.2E+01	1.7E+04	2.3E+03	4.1E+02
67	343.0462	2.40	Quercetin-etenol [M-H]-: 0.598	0.8	-	-	-	-	-	2.3E+02	6.0E+00	1.2E+01
68	413.0879	2.44	Apigenin-di-propenodiol [M-H]-: 0.616	0.2	-	-	9.1E+01	1.5E+01	9.1E+02	-	-	2.5E+02
69	269.0444	2.46	Apigenin [M-H]-: 0.508	-4.3	-	-	3.6E+01	2.7E+01	1.4E+02	3.5E+01	8.6E+02	6.1E+01
70	284.0306	2.46	Kaempferol / Luteolin / Datiscetin [M-H].-: 0.587	1.2	-	-	8.1E+00	9.3E+00	-	4.9E+01	4.6E+03	-
71	383.0777	2.47	Apigenin-propenodiol + etenol [M-H]-: 0.580	1.2	-	-	-	7.4E+00	2.3E+02	-	-	-
72	300.0273	2.48	Quercetin [M-H].-: 0.599	-0.8	-	-	8.1E+03	4.3E+01	6.8E+01	2.1E+04	1.4E+03	6.5E+02
73	343.0445	2.48	Quercetin-etenol [M-H]-: 0.480	-4.2	-	-	6.9E+01	-	-	2.2E+02	6.6E+00	-
74	353.0676	2.49	Apigenin-di-etenol[M-H]-: 0.533	2.6	-	-	-	-	3.3E+02	-	-	-
75	311.0553	2.51	Apigenin-etenol [M-H]-: 0.544	-2.6	-	-	-	3.6E+01	4.7E+03	-	-	1.5E+03
76	269.0445	2.53	Apigenin [M-H]-: 0.524	-3.9	-	-	2.1E+01	4.3E+01	3.2E+02	3.7E+01	6.8E+02	9.6E+01
77	341.0657	2.53	Apigenin-propenodiol [M-H]-: 0.530	-2.9	-	-	9.7E+00	2.5E+02	7.8E+02	1.0E+01	-	1.1E+02
78	300.0272	2.54	Quercetin [M-H].-: 0.589	-1.2	-	-	1.1E+04	1.1E+03	3.5E+02	2.8E+04	7.7E+03	1.7E+03

79	353.0669	2.54	Apigenin-di-etenol[M-H]-: 0.608	0.6	-	-	-	-	3.5E+02	-	-	6.1E+01
80	429.0829	2.54	Kaempferol / Luteolin / Datiscetin-di-propenodiol [M-H]-: 0.607;Myricetin-propenodiol + etenol [M-H]-: 0.607	0.4	-	-	-	-	1.1E+01	-	1.1E+02	-
81	301.0319	2.55	Quercetin [M-H]-: 0.280	-11.6	-	-	1.2E+04	4.0E+02	1.6E+01	8.8E+03	1.1E+03	5.6E+02
82	383.0778	2.55	Apigenin-propenodiol + etenol [M-H]-: 0.576	1.5	-	-	-	-	2.0E+02	-	-	4.7E+01
83	461.0724	2.55	Myricetin-di-propenodiol [M-H]-: 0.609	-0.3	-	-	7.2E+01	-	-	3.2E+02	-	-
84	284.0306	2.56	Kaempferol / Luteolin / Datiscetin [M-H].-: 0.597	1.2	-	-	7.2E+01	2.3E+01	2.2E+02	1.0E+02	5.2E+03	1.8E+03
85	341.0657	2.58	Apigenin-propenodiol [M-H]-: 0.529	-2.9	-	-	1.6E+01	9.1E+02	1.2E+02	1.5E+01	-	7.8E+01
86	285.0384	2.60	Kaempferol / Luteolin / Datiscetin [M-H]-: 0.423	-7.2	-	-	2.5E+02	2.7E+02	6.0E+02	1.5E+02	-	2.0E+03
87	300.0269	2.60	Quercetin [M-H].-: 0.562	-2.2	-	-	3.5E+03	9.2E+01	5.4E+00	7.6E+03	1.7E+04	2.5E+02
88	447.0941	2.60	Taxifolin-di-propenodiol [M-H]-: 0.553	1.8	-	-	-	-	3.3E+02	8.5E+01	-	-
89	269.0444	2.62	Apigenin [M-H]-: 0.518	-4.3	-	-	1.0E+01	6.2E+01	4.7E+01	3.9E+01	1.6E+02	6.1E+01
90	289.0711	2.62	Catechin/Epicatechin [M-H]-: 0.569	-2.3	-	-	6.3E+02	5.5E+02	1.4E+01	2.1E+02	-	3.0E+02
91	327.0506	2.63	Kaempferol / Luteolin / Datiscetin -etenol [M-H]-: 0.588	-1.3	-	-	1.4E+01	5.5E+01	7.2E+01	5.6E+01	7.1E+01	3.7E+02
92	284.0320	2.64	Kaempferol / Luteolin / Datiscetin [M-H].-: 0.454	6.1	-	-	2.0E+02	5.2E+01	4.7E+02	1.8E+03	4.9E+03	7.7E+03
93	341.0656	2.64	Apigenin-propenodiol [M-H]-: 0.525	-3.2	-	-	6.4E+00	1.7E+02	5.1E+02	-	-	3.0E+01
94	285.0385	2.65	Kaempferol / Luteolin / Datiscetin [M-H]-: 0.432	-6.9	-	-	1.9E+02	9.9E+01	1.4E+02	3.1E+03	6.5E+03	2.2E+03
95	391.0702	2.65	Myricetin-propenodiol [M-H]-: 0.319	8.0	-	-	-	-	-	-	-	1.1E+03
96	300.0266	2.71	Quercetin [M-H].-: 0.538	-3.2	-	-	1.5E+03	5.4E+02	2.3E+02	1.2E+04	2.2E+03	-
97	301.0322	2.71	Quercetin [M-H]-: 0.313	-10.6	-	-	7.1E+02	2.6E+02	5.5E+01	4.4E+03	8.2E+02	-

98	285.0382	2.72	Kaempferol / Luteolin / Datiscetin [M-H]-: 0.410	-7.9	-	-	1.8E+03	1.1E+02	6.6E+01	4.6E+03	8.8E+03	2.3E+03
99	327.0500	2.72	Kaempferol / Luteolin / Datiscetin - etenol [M-H]-: 0.532	-3.1	-	-	4.8E+01	3.8E+01	6.3E+01	1.1E+02	1.0E+02	7.6E+01
100	284.0316	2.73	Kaempferol / Luteolin / Datiscetin [M-H].-: 0.502	4.7	-	-	2.4E+03	-	5.8E+01	4.0E+03	7.0E+03	1.7E+03
101	300.0266	2.74	Quercetin [M-H].-: 0.542	-3.2	-	-	3.8E+03	5.4E+02	2.4E+02	3.1E+04	7.2E+03	2.2E+03
102	447.0928	2.76	Taxifolin-di-propenodiol [M-H]-: 0.591	-1.1	-	-	8.5E+02	5.7E+02	9.2E+01	1.1E+03	1.4E+01	5.8E+02
103	285.0381	2.77	Kaempferol / Luteolin / Datiscetin [M-H]-: 0.403	-8.3	-	-	6.3E+02	3.9E+02	3.0E+01	5.6E+02	-	6.8E+02
104	284.0317	2.78	Kaempferol / Luteolin / Datiscetin [M-H].-: 0.491	5.1	-	-	1.4E+03	9.2E+02	6.1E+01	1.1E+03	1.7E+04	6.4E+02
105	289.0709	2.79	Catechin/Epicatechin [M-H]-: 0.550	-3.0	-	-	2.1E+02	4.0E+02	2.1E+01	1.4E+02	-	7.9E+02
106	383.0789	2.79	Apigenin-propenodiol + etenol [M-H]-: 0.471	4.3	-	-	-	-	7.6E+02	7.7E+00	-	-
107	300.0265	2.81	Quercetin [M-H].-: 0.536	-3.5	-	-	2.8E+02	8.5E+02	1.2E+02	4.2E+03	1.5E+04	-
108	311.0557	2.81	Apigenin-etenol [M-H]-: 0.598	-1.3	-	-	-	7.8E+00	7.5E+01	-	8.2E+00	2.8E+02
109	353.0664	2.81	Apigenin-di-etenol[M-H]-: 0.609	-0.8	-	-	-	-	9.7E+02	-	-	8.3E+00
110	413.0872	2.81	Apigenin-di-propenodiol [M-H]-: 0.577	-1.5	-	-	3.7E+01	1.5E+01	7.3E+01	-	-	3.1E+02
111	461.0713	2.82	Myricetin-di-propenodiol [M-H]-: 0.520	-2.7	-	-	2.6E+02	-	-	-	-	3.1E+01
112	269.0443	2.83	Apigenin [M-H]-: 0.521	-4.6	-	-	2.4E+01	2.6E+01	6.5E+01	5.7E+01	7.3E+01	2.4E+03
113	341.0649	2.83	Apigenin-propenodiol [M-H]-: 0.460	-5.2	-	-	2.6E+01	1.1E+03	-	-	-	2.4E+01
114	284.0321	2.84	Kaempferol / Luteolin / Datiscetin [M-H].-: 0.457	6.5	-	-	1.2E+03	1.6E+02	-	8.4E+02	7.2E+04	4.0E+02
115	327.0505	2.84	Kaempferol / Luteolin / Datiscetin - etenol [M-H]-: 0.592	-1.6	-	-	3.9E+01	-	6.3E+01	-	2.3E+02	2.0E+01
116	399.0706	2.86	Kaempferol / Luteolin / Datiscetin-propenodiol + etenol [M-H]-: 0.485	-3.9	-	-	-	-	-	-	1.1E+03	-

117	417.0817	2.86	Taxifolin-propenodiol + etenol [M-H]-: 0.541	-2.4	-	-	-	-	-	-	2.5E+02	-
118	300.0272	2.87	Quercetin [M-H].-: 0.607	-1.2	-	-	8.4E+03	6.5E+03	1.4E+02	3.0E+04	2.4E+03	2.4E+03
119	447.0930	2.88	Taxifolin-di-propenodiol [M-H]-: 0.616	-0.6	-	-	6.8E+03	5.2E+03	4.2E+01	2.9E+04	4.1E+03	1.6E+03
120	268.0365	2.89	Apigenin [M-H].-: 0.520	-4.6	-	-	-	-	2.8E+02	1.0E+01	7.0E+00	6.2E+00
121	375.0744	2.89	Taxifolin-propenodiol [M-H]-: 0.417	6.0	-	-	-	-	-	-	-	2.4E+03
122	445.0779	2.89	Quercetin-di-propenodiol [M-H]-: 0.616	0.6	-	-	2.4E+01	2.1E+01	-	2.6E+02	8.7E+00	-
123	269.0440	2.91	Apigenin [M-H]-: 0.488	-5.8	-	-	7.3E+00	9.7E+01	1.3E+02	6.0E+01	3.5E+01	2.8E+01
124	284.0319	2.96	Kaempferol / Luteolin / Datiscetin [M-H].-: 0.484	5.8	-	-	1.1E+03	1.9E+02	1.1E+02	3.3E+03	4.6E+03	8.7E+02
125	417.0814	2.96	Taxifolin-propenodiol + etenol [M-H]-: 0.519	-3.2	-	-	6.6E+02	7.1E+01	-	1.9E+03	2.9E+03	5.9E+02
126	284.0310	3.01	Kaempferol / Luteolin / Datiscetin [M-H].-: 0.573	2.6	-	-	2.7E+02	4.7E+02	4.3E+01	8.9E+02	2.1E+04	-
127	417.0822	3.01	Taxifolin-propenodiol + etenol [M-H]-: 0.601	-1.2	-	-	1.7E+02	2.0E+02	-	3.5E+02	5.0E+03	9.3E+00
128	300.0265	3.04	Quercetin [M-H].-: 0.547	-3.5	-	-	6.6E+02	-	-	1.2E+03	8.0E+01	4.0E+01
129	341.0656	3.05	Apigenin-propenodiol [M-H]-: 0.544	-3.2	-	-	8.0E+00	6.0E+02	5.0E+01	8.9E+01	-	2.1E+01
130	399.0706	3.06	Kaempferol / Luteolin / Datiscetin-propenodiol + etenol [M-H]-: 0.496	-3.9	-	-	-	-	-	-	8.3E+02	-
131	284.0316	3.07	Kaempferol / Luteolin / Datiscetin [M-H].-: 0.523	4.7	-	-	8.7E+01	3.9E+02	6.2E+00	6.3E+02	5.5E+04	9.6E+01
132	417.0819	3.07	Taxifolin-propenodiol + etenol [M-H]-: 0.569	-2.0	-	-	2.8E+01	2.4E+02	-	3.6E+02	1.3E+02	8.3E+00
133	285.0383	3.11	Kaempferol / Luteolin / Datiscetin [M-H]-: 0.443	-7.6	-	-	1.0E+02	1.6E+02	3.5E+02	-	5.4E+02	-
134	353.0674	3.11	Apigenin-di-etenol[M-H]-: 0.582	2.0	-	-	-	-	6.6E+02	-	-	-
135	447.0933	3.11	Taxifolin-di-propenodiol [M-H]-: 0.651	0.0	-	-	2.9E+01	-	1.1E+02	1.6E+02	1.9E+01	2.3E+02

136	284.0311	3.12	Kaempferol / Luteolin / Datiscetin [M-H].-: 0.571	3.0	-	-	1.7E+01	7.1E+01	2.4E+02	-	2.1E+03	3.0E+01
137	383.0768	3.13	Apigenin-propenodiol + etenol [M-H]-: 0.617	-1.1	-	-	-	1.3E+01	3.7E+02	1.4E+01	-	-
138	341.0656	3.16	Apigenin-propenodiol [M-H]-: 0.550	-3.2	-	-	9.6E+00	2.9E+02	3.4E+01	-	-	1.1E+01
139	284.0311	3.17	Kaempferol / Luteolin / Datiscetin [M-H].-: 0.570	3.0	-	-	1.2E+03	1.3E+04	-	9.4E+03	1.0E+03	1.5E+03
140	285.0385	3.17	Kaempferol / Luteolin / Datiscetin [M-H]-: 0.462	-6.9	-	-	1.7E+03	1.5E+04	2.6E+01	1.3E+04	1.8E+03	2.1E+03
141	341.0656	3.22	Apigenin-propenodiol [M-H]-: 0.555	-3.2	-	-	1.7E+01	6.7E+02	1.8E+01	7.1E+01	-	6.9E+00
142	284.0316	3.25	Kaempferol / Luteolin / Datiscetin [M-H].-: 0.532	4.7	-	-	2.4E+02	3.6E+02	-	2.5E+02	2.2E+02	5.2E+01
143	285.0386	3.25	Kaempferol / Luteolin / Datiscetin [M-H]-: 0.480	-6.5	-	-	2.0E+02	5.7E+02	4.1E+01	5.4E+02	1.8E+02	1.8E+02
144	447.0932	3.25	Taxifolin-di-propenodiol [M-H]-: 0.649	-0.2	-	-	7.9E+00	3.5E+02	4.6E+01	1.2E+02	-	-
145	300.0261	3.30	Quercetin [M-H].-: 0.523	-4.8	-	-	3.2E+02	2.8E+01	3.0E+01	4.3E+02	1.7E+01	8.7E+00
146	447.0932	3.30	Taxifolin-di-propenodiol [M-H]-: 0.659	-0.2	-	-	5.3E+00	2.2E+02	3.5E+01	4.7E+01	5.2E+00	-
147	284.0317	3.31	Kaempferol / Luteolin / Datiscetin [M-H].-: 0.519	5.1	-	-	2.2E+01	1.7E+02	-	8.4E+01	2.8E+02	1.5E+01
148	285.0389	3.31	Kaempferol / Luteolin / Datiscetin [M-H]-: 0.505	-5.5	-	-	9.2E+01	2.9E+02	6.0E+01	2.0E+02	2.2E+02	7.0E+01
149	301.0336	3.31	Quercetin [M-H]-: 0.485	-5.9	-	-	1.8E+02	2.2E+01	-	3.6E+02	6.6E+00	-
150	300.0264	3.35	Quercetin [M-H].-: 0.548	-3.8	-	-	1.6E+03	2.4E+01	-	1.8E+03	6.2E+01	7.4E+00
151	341.0658	3.36	Apigenin-propenodiol [M-H]-: 0.583	-2.6	-	-	6.2E+00	2.8E+02	3.1E+01	3.1E+01	-	-
152	301.0327	3.37	Quercetin [M-H]-: 0.399	-8.9	-	-	8.4E+02	3.2E+01	-	9.6E+02	1.9E+01	-
153	301.0325	3.43	Quercetin [M-H]-: 0.384	-9.6	-	-	2.1E+02	1.7E+01	-	6.3E+02	6.8E+00	-
154	300.0255	3.44	Quercetin [M-H].-: 0.466	-6.8	-	-	4.2E+02	7.9E+00	-	5.2E+02	3.2E+01	-
155	268.0345	3.48	Apigenin [M-H].-: 0.349	-12.0	-	-	-	2.5E+02	-	8.3E+00	1.7E+01	-

156	300.0258	3.49	Quercetin [M-H].-: 0.503	-5.8	-	-	7.8E+02	8.4E+00	-	5.0E+02	2.0E+01	-
157	284.0307	3.53	Kaempferol / Luteolin / Datiscetin [M-H].-: 0.636	1.5	-	-	6.2E+02	1.7E+03	1.9E+01	5.0E+02	1.5E+02	6.6E+00
158	285.0384	3.54	Kaempferol / Luteolin / Datiscetin [M-H]-: 0.468	-7.2	-	-	7.2E+02	1.8E+03	5.5E+01	6.8E+02	2.1E+02	8.1E+01
159	301.0337	3.60	Quercetin [M-H]-: 0.514	-5.6	-	-	1.3E+02	2.0E+02	-	9.6E+03	2.1E+01	8.2E+01
160	285.0387	3.67	Kaempferol / Luteolin / Datiscetin [M-H]-: 0.508	-6.2	-	-	2.1E+02	8.5E+02	1.1E+02	2.1E+02	1.9E+02	1.4E+02
161	284.0313	3.69	Kaempferol / Luteolin / Datiscetin [M-H].-: 0.576	3.7	-	-	2.2E+02	7.7E+02	4.6E+01	8.7E+01	2.9E+01	2.6E+01
162	284.0309	3.85	Kaempferol / Luteolin / Datiscetin [M-H].-: 0.632	2.3	-	-	1.1E+01	1.6E+02	-	1.3E+02	6.1E+01	7.7E+00
163	284.0312	3.99	Kaempferol / Luteolin / Datiscetin [M-H].-: 0.604	3.3	-	-	1.6E+01	1.9E+02	3.6E+01	6.9E+01	9.4E+01	5.0E+00
164	285.0384	4.03	Kaempferol / Luteolin / Datiscetin [M-H]-: 0.496	-7.2	-	-	1.0E+02	2.1E+03	1.4E+02	4.6E+02	2.4E+02	3.8E+02
165	284.0307	4.04	Kaempferol / Luteolin / Datiscetin [M-H].-: 0.663	1.5	-	-	2.3E+01	1.0E+03	1.5E+01	1.4E+02	1.1E+02	8.3E+01
166	285.0385	4.11	Kaempferol / Luteolin / Datiscetin [M-H]-: 0.509	-6.9	-	-	8.6E+01	8.1E+02	7.6E+01	4.1E+03	5.9E+02	3.8E+02
167	300.0262	4.14	Quercetin [M-H].-: 0.572	-4.5	-	-	4.4E+02	4.4E+01	-	1.1E+03	2.1E+02	8.2E+01
168	301.0336	4.14	Quercetin [M-H]-: 0.527	-5.9	-	-	3.2E+02	4.8E+01	6.6E+00	1.0E+03	2.2E+02	7.6E+01
169	447.0928	4.14	Taxifolin-di-propenodiol [M-H]-: 0.658	-1.1	-	-	5.5E+01	8.1E+00	-	2.0E+02	4.8E+01	2.6E+01
170	284.0311	4.23	Kaempferol / Luteolin / Datiscetin [M-H].-: 0.625	3.0	-	-	1.5E+01	3.5E+03	9.1E+00	1.1E+02	7.9E+01	4.0E+01
171	285.0389	4.23	Kaempferol / Luteolin / Datiscetin [M-H]-: 0.552	-5.5	-	-	6.8E+01	3.4E+03	9.5E+01	3.8E+02	-	1.7E+02

172	447.0919	4.24	Taxifolin-di-propenodiol [M-H]-: 0.577	-3.1	-	-	1.9E+01	1.4E+02	-	6.5E+01	1.2E+01	-
173	300.0258	4.27	Quercetin [M-H].-: 0.538	-5.8	-	-	2.7E+01	5.6E+00	-	2.7E+02	1.6E+02	6.2E+01
174	285.0383	4.42	Kaempferol / Luteolin / Datiscetin [M-H]-: 0.504	-7.6	-	-	3.4E+02	2.7E+03	9.0E+02	5.3E+03	2.7E+03	9.5E+02
175	284.0314	4.46	Kaempferol / Luteolin / Datiscetin [M-H].-: 0.612	4.0	-	-	1.7E+02	2.6E+03	4.8E+02	4.4E+03	2.2E+03	7.1E+02
176	285.0383	4.47	Kaempferol / Luteolin / Datiscetin [M-H]-: 0.512	-7.6	-	-	8.2E+02	7.9E+02	1.3E+03	1.1E+04	5.9E+03	2.0E+03
177	284.0317	4.54	Kaempferol / Luteolin / Datiscetin [M-H].-: 0.578	5.1	-	-	-	1.1E+03	1.5E+02	5.7E+01	-	1.5E+01
178	285.0387	4.56	Kaempferol / Luteolin / Datiscetin [M-H]-: 0.549	-6.2	-	-	1.8E+02	1.2E+03	2.6E+02	2.1E+02	1.2E+03	3.4E+02
179	284.0314	4.60	Kaempferol / Luteolin / Datiscetin [M-H].-: 0.619	4.0	-	-	1.0E+02	2.9E+02	-	3.8E+02	1.9E+03	1.7E+02
180	285.0387	4.60	Kaempferol / Luteolin / Datiscetin [M-H]-: 0.556	-6.2	-	-	2.1E+02	2.8E+02	9.9E+01	8.6E+02	4.3E+03	3.7E+02
181	284.0316	4.81	Kaempferol / Luteolin / Datiscetin [M-H].-: 0.611	4.7	-	-	4.1E+01	5.5E+02	7.2E+01	1.3E+02	1.8E+02	5.2E+01
182	285.0391	4.81	Kaempferol / Luteolin / Datiscetin [M-H]-: 0.603	-4.8	-	-	8.4E+01	7.4E+02	1.3E+02	3.4E+02	3.5E+02	1.6E+02
183	301.0328	5.07	Quercetin [M-H]-: 0.491	-8.6	-	-	1.2E+02	-	-	7.5E+02	3.1E+02	4.1E+01
184	447.0915	5.07	Taxifolin-di-propenodiol [M-H]-: 0.579	-4.0	-	-	1.6E+01	-	-	1.2E+02	4.6E+01	5.0E+00
185	300.0264	5.08	Quercetin [M-H].-: 0.637	-3.8	-	-	2.2E+02	-	-	1.3E+03	4.3E+02	5.3E+01
186	300.0266	5.18	Quercetin [M-H].-: 0.664	-3.2	-	-	1.1E+01	-	-	8.0E+01	3.3E+02	-
187	447.0932	5.25	Taxifolin-di-propenodiol [M-H]-: 0.758	-0.2	-	-	1.2E+02	-	2.9E+01	1.8E+02	8.6E+01	2.4E+01
188	300.0262	5.26	Quercetin [M-H].-: 0.626	-4.5	-	-	1.9E+03	9.2E+00	7.3E+02	3.1E+03	1.3E+03	4.2E+02
189	301.0326	5.26	Quercetin [M-H]-: 0.480	-9.2	-	-	1.6E+03	1.2E+01	6.0E+02	2.7E+03	1.0E+03	3.6E+02

190	284.0312	5.30	Kaempferol / Luteolin / Datiscetin [M-H].-: 0.670	3.3	-	-	2.2E+02	2.4E+01	2.3E+02	6.6E+02	1.5E+03	9.9E+01
191	300.0269	5.37	Quercetin [M-H].-: 0.702	-2.2	-	-	2.2E+02	-	1.8E+02	1.4E+02	5.8E+02	-
192	284.0308	5.38	Kaempferol / Luteolin / Datiscetin [M-H].-: 0.717	1.9	-	-	4.7E+02	4.9E+01	1.1E+03	7.9E+03	7.4E+03	5.0E+02
193	285.0383	5.38	Kaempferol / Luteolin / Datiscetin [M-H]-: 0.552	-7.6	-	-	6.2E+02	9.0E+01	1.8E+03	1.2E+04	1.3E+04	7.2E+02
194	413.0874	5.38	Apigenin-di-propenodiol [M-H]-: 0.728	-1.0	-	-	-	-	-	2.3E+02	2.2E+02	1.6E+01
195	285.0385	5.43	Kaempferol / Luteolin / Datiscetin [M-H]-: 0.578	-6.9	-	-	5.9E+01	6.3E+01	4.5E+02	2.4E+03	2.7E+03	2.8E+02
196	300.0270	5.43	Quercetin [M-H].-: 0.720	-1.8	-	-	4.6E+02	-	1.7E+02	3.6E+02	2.0E+02	8.8E+01
197	301.0339	5.43	Quercetin [M-H]-: 0.623	-4.9	-	-	3.8E+02	-	1.5E+02	2.7E+02	1.5E+02	8.4E+01
198	284.0309	5.50	Kaempferol / Luteolin / Datiscetin [M-H].-: 0.709	2.3	-	-	3.0E+01	1.7E+01	4.5E+03	-	4.1E+03	2.7E+01
199	285.0386	5.50	Kaempferol / Luteolin / Datiscetin [M-H]-: 0.585	-6.5	-	-	7.4E+01	2.7E+01	2.1E+04	-	7.0E+03	5.7E+01
200	300.0256	5.52	Quercetin [M-H].-: 0.583	-6.5	-	-	4.0E+01	-	7.1E+00	1.4E+02	2.4E+02	-
201	413.0869	5.56	Apigenin-di-propenodiol [M-H]-: 0.689	-2.2	-	-	7.6E+01	-	2.3E+02	3.8E+02	4.3E+02	1.1E+02
202	284.0305	5.57	Kaempferol / Luteolin / Datiscetin [M-H].-: 0.755	0.8	-	-	1.2E+03	8.9E+01	1.8E+03	8.0E+03	1.0E+04	2.2E+03
203	285.0383	5.57	Kaempferol / Luteolin / Datiscetin [M-H]-: 0.557	-7.6	-	-	4.4E+03	2.8E+02	7.5E+03	2.3E+04	2.8E+04	8.0E+03
204	284.0307	5.58	Kaempferol / Luteolin / Datiscetin [M-H].-: 0.737	1.5	-	-	6.6E+02	5.9E+01	1.5E+03	4.1E+03	-	5.9E+02
205	285.0383	5.60	Kaempferol / Luteolin / Datiscetin [M-H]-: 0.567	-7.6	-	-	2.5E+03	2.1E+02	6.6E+03	4.3E+03	7.6E+03	1.9E+03

206	284.0316	5.66	Kaempferol / Luteolin / Datiscetin [M-H].-: 0.649	4.7	-	-	4.2E+02	2.6E+01	7.3E+02	5.4E+02	1.2E+03	2.3E+02
207	285.0382	5.66	Kaempferol / Luteolin / Datiscetin [M-H]-: 0.556	-7.9	-	-	1.3E+03	7.3E+01	2.7E+03	1.3E+03	-	6.1E+02
208	413.0880	5.70	Apigenin-di-propenodiol [M-H]-: 0.768	0.5	-	-	3.3E+01	-	9.1E+01	2.3E+01	3.4E+02	3.1E+01
209	284.0309	5.72	Kaempferol / Luteolin / Datiscetin [M-H].-: 0.721	2.3	-	-	5.7E+02	4.1E+01	1.7E+03	2.3E+03	6.2E+03	5.7E+02
210	285.0381	5.72	Kaempferol / Luteolin / Datiscetin [M-H]-: 0.553	-8.3	-	-	2.1E+03	1.0E+02	6.2E+03	4.3E+03	2.2E+04	1.8E+03
211	284.0307	5.86	Kaempferol / Luteolin / Datiscetin [M-H].-: 0.746	1.5	-	-	2.3E+02	5.1E+01	6.6E+02	1.4E+03	2.9E+03	2.8E+02
212	285.0383	5.86	Kaempferol / Luteolin / Datiscetin [M-H]-: 0.577	-7.6	-	-	2.7E+02	6.8E+01	8.0E+02	2.4E+03	1.0E+04	3.1E+02
213	285.0386	5.94	Kaempferol / Luteolin / Datiscetin [M-H]-: 0.616	-6.5	-	-	1.3E+02	-	1.4E+03	1.2E+03	1.8E+03	1.6E+02
214	285.0383	5.99	Kaempferol / Luteolin / Datiscetin [M-H]-: 0.586	-7.6	-	-	2.7E+02	2.4E+01	5.5E+02	3.4E+03	2.5E+03	3.3E+02
215	284.0314	6.02	Kaempferol / Luteolin / Datiscetin [M-H].-: 0.689	4.0	-	-	1.3E+02	3.9E+01	7.3E+02	8.8E+02	9.7E+02	1.2E+02
216	285.0383	6.04	Kaempferol / Luteolin / Datiscetin [M-H]-: 0.586	-7.6	-	-	1.7E+02	3.6E+01	6.0E+02	6.6E+02	1.9E+03	1.8E+02

(-); peak area equal 0.

Table S4. Annotation numbers for MS^1- and MS^2-spectra level of *Ocotea* species

	O. diospyrifolia	*O. lancifolia*	*O. notata*	*O. odorifera*	*O. porosa*	*O. guianensis*
MS^1 annotations (precursors)	66	67	51	72	80	47
MS^2 annotations (aglycones)	59	56	55	73	87	51
